# Genetic disruption of the circadian gene *Bmal1* in the intestinal epithelium reduces colonic inflammation

**DOI:** 10.1038/s44319-025-00464-y

**Published:** 2025-04-30

**Authors:** Shan Hua, Ze Zhang, Zhe Zhang, Liansheng Liu, Shicheng Yu, Yanhui Xiao, Yuan Liu, Siting Wei, Ying Xu, Ye-Guang Chen

**Affiliations:** 1 Guangzhou National Laboratory, Guangzhou, 510005 China; 2 The State Key Laboratory of Membrane Biology, Tsinghua-Peking Center for Life Sciences, School of Life Sciences, Tsinghua University, Beijing, 100084 China; 3 Cambridge-Su Genomic Resource Center, Soochow University, Suzhou, Jiangsu 215123 China; 4 The MOE Basic Research and Innovation Center for the Targeted Therapeutics of Solid Tumors, School of Basic Medical Sciences, Institute of Biomedical Innovation, Jiangxi Medical College, Nanchang University, Nanchang, 330031 China

**Keywords:** Circadian Rhythm, Bmal1, Intestinal Homeostasis, Colitis, Chromatin, Transcription & Genomics, Immunology, Molecular Biology of Disease

## Abstract

Disruption of the circadian clock is associated with the development of inflammatory bowel disease (IBD), but the underlying mechanisms remain unclear. Here, we observe that mice in the early active phase (Zeitgeber time 12, ZT12) of the circadian clock are more tolerant to dextran sodium sulfate (DSS)-induced colitis, compared to those in the early resting phase (ZT0). The expression of the circadian gene *Bmal1* peaks in the early resting phase and declines in the early active phase. *Bmal1* knockout in the intestinal epithelium reduces DSS-induced inflammatory symptoms. Mechanistically, BMAL1 promotes apoptosis by binding to apoptosis-related genes, including *Bax, p53,* and *Bak1*, and promotes their expression. Intriguingly, we observe circadian apoptotic rhythms in the homeostatic intestinal epithelium, while *Bmal1* deletion reduces cell apoptosis. Consistently, reducing *Bmal1* expression by the REV-ERBα agonist SR9009 has the best therapeutic efficacy against DSS-induced colitis at ZT0. Collectively, our data demonstrate that the *Bmal1*-centered circadian clock is involved in intestinal injury repair.

## Synopsis

Disruption of the circadian clock is associated with the development of inflammatory bowel disease. Mice in the active phase are more resistant to DSS-induced colitis, while the circadian regulator BMAL1 activates apoptotic genes to promote colitis.

- *Bmal1* deficiency in the intestinal epithelium reduces DSS-induced colitis symptoms.
- Apoptosis shows a *Bmal1*-dependent circadian rhythm pattern in the intestinal epithelium
- Inhibition of *Bmal1* expression at ZT0 has the best efficacy in DSS-induced colitis.
- Targeting BMAL1 may be a promising approach to treat inflammation-related gastrointestinal tract diseases.

## Abstract (web summary)

Disruption of the circadian clock is associated with the development of inflammatory bowel disease. Mice in the active phase are more resistant to DSS-induced colitis, while the circadian regulator BMAL1 activates apoptotic genes to promote colitis.

## Graphical abstract

**Figure:**
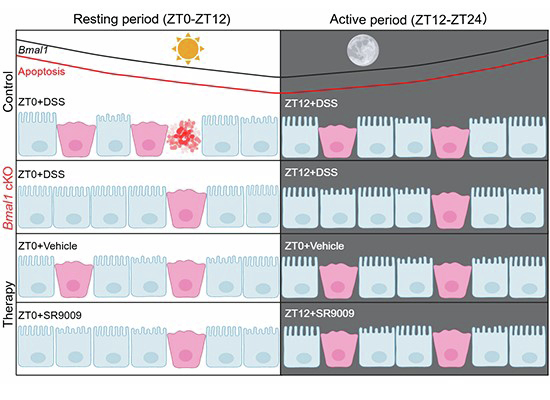

**Figure:**
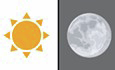

## Introduction

Circadian rhythms, a nearly ubiquitous feature of eukaryotic life, is intrinsic approximately 24-h cyclic oscillations of biochemical, physiological, and behavioral functions in organisms (Sancar et al, 2015). In mammals, the central pacemaker situated in the suprachiasmatic nucleus (SCN) of the brain serves as the coordinator to align the clocks distributed among other brain regions and peripheral tissues (Mohawk et al, 2012). The mammalian circadian rhythm is composed of time-delayed transcription-translation feedback loops (TTFL) at the molecular level (Hastings et al, 2003; Partch et al, 2014; Patke et al, 2020; Reppert and Weaver, 2002). The transcription factors BMAL1 and CLOCK form the activation arm, and the cryptochromes (CRY1 and CRY2) and PERs (PER1, PER2, and possibly PER3) form the repression arm of this TTFL (Ye et al, 2014). The transcription factors BMAL1 and CLOCK form a heterodimer that promotes the transcription of clock control genes (CCGs) including PERs and CRYs (Shearman et al, 2000). Once PERs and CRYs are accumulated, they in turn repress the transcriptional activity of the BMAL1/CLOCK complex and thus reduce the expression of CCGs (King and Takahashi, 2000). This intricate, time-delayed feedback mechanism results in a roughly 24-h oscillation in gene expression, encompassing 43% of protein-coding genes (Zhang et al, 2014). The circadian regulation of hormonal, inflammatory, and metabolic processes is crucial for maintaining mammalian organic health (Bass and Lazar, 2016; Janich et al, 2014). Disturbance of circadian rhythms has been associated with various diseases, including cancers and metabolic disorders (Kettner et al, 2016; Papagiannakopoulos et al, 2016).

The intestine plays critical roles in food digestion, nutrient absorption, waste elimination, and defense against foreign pathogens (Clevers, 2013; Qi and Chen, 2015; Wang and Chen, 2018; Zhu et al, 2021). Disruption of its homeostasis is frequently associated with the onset of intestinal diseases, including inflammatory bowel disease (IBD), irritable bowel syndrome, and colorectal cancer (Yang et al, 2025). IBD, including Crohn’s disease (CD) and ulcerative colitis (UC), represents a chronic, recurrent inflammatory condition in the gastrointestinal tract, characterized by persistent pain, diarrhea, and bloody stools (Kaser et al, 2010; McGuckin et al, 2009). Although the existing etiology of UC is largely uncertain, hyperactivation of the mucosal immune system and the subsequent production of pathologic cytokines were shown to contribute to this disease (Baumgart and Carding, 2007; Engel and Neurath, 2010; Salim and Soderholm, 2011). Circadian rhythm disruption may serve as a predisposing factor for the exacerbation of IBD (Swanson et al, 2011). Shift workers, whose circadian rhythms are disrupted, have been shown to have a higher incidence of IBD (Sonnenberg, 1990). Furthermore, the disruption of the circadian clock by sleep disturbance increases the risk of IBD (Ananthakrishnan et al, 2014) and disease severity in patients (Graff et al, 2011; Keefer et al, 2006). However, the underlying mechanisms that circadian rhythm regulates the progression of IBD remain largely elusive.

*Bmal1* is a core circadian gene and functions as a transcriptional activator, and its deletion leads to disrupted expression of other circadian genes (e.g., *Clock*, *Per1*, *Cry1*) and dysregulation of the rhythm in mice (Yu et al, 2021). *Bmal1*-deleted macrophages alter the cytoskeletal structure and enhance overall phagocytic function (Kitchen et al, 2020). *Bmal1* also regulates mitochondrial autophagy in cardiomyocytes and affects the level of oxidative phosphorylation by inducing the expression of the mitochondrial autophagy gene *Bnip3* (Li et al, 2020). In the intestinal epithelium, *Bmal1* promotes dietary fat absorption by activating *Dgat2* expression through direct binding to its promoter (Yu et al, 2021). Additionally, *Bmal1* participates in the development of colon cancer by regulating Wnt and Hippo signaling pathways (Chun et al, 2022; Stokes et al, 2021). Despite these, its precise role in intestinal inflammation remains elusive.

To investigate the functional relevance of the intestinal clock in intestinal inflammation, we generated intestinal epithelium-specific *Bmal1* knockout mice and examined its effect on dextran sodium sulfate (DSS)-induced colitis. We found that mice in the active phase were more resistant to DSS-induced colitis. *Bmal1-*deletion rendered intestinal epithelium more tolerant to DSS-induced acute colitis and reduced cell death. RNA-seq and CUT&Tag-seq analyses revealed that *Bmal1* transcriptionally activated the expression of apoptosis-related genes, such as *p53*, *Bax,* and *Bak1*. Surprisingly, we found that cell apoptosis also exhibited a circadian rhythm in homeostatic intestinal epithelium. Moreover, reducing *Bmal1* expression by SR9009 at ZT0 had the best efficacy in alleviating DSS-induced colitis. Analysis of public datasets of human patients with inflammatory bowel disease (IBD) showed that BMAL1 was downregulated in IBD patients, suggesting a correlation between BMAL1 and IBD. Overall, our study highlights the pivotal role of the circadian gene *Bmal1* in the regulation of colitis and provides a potential drug target for colitis treatment.

## Results

### Mice in the active circadian phase are more resistant to DSS-induced colitis

**Figure 1:**
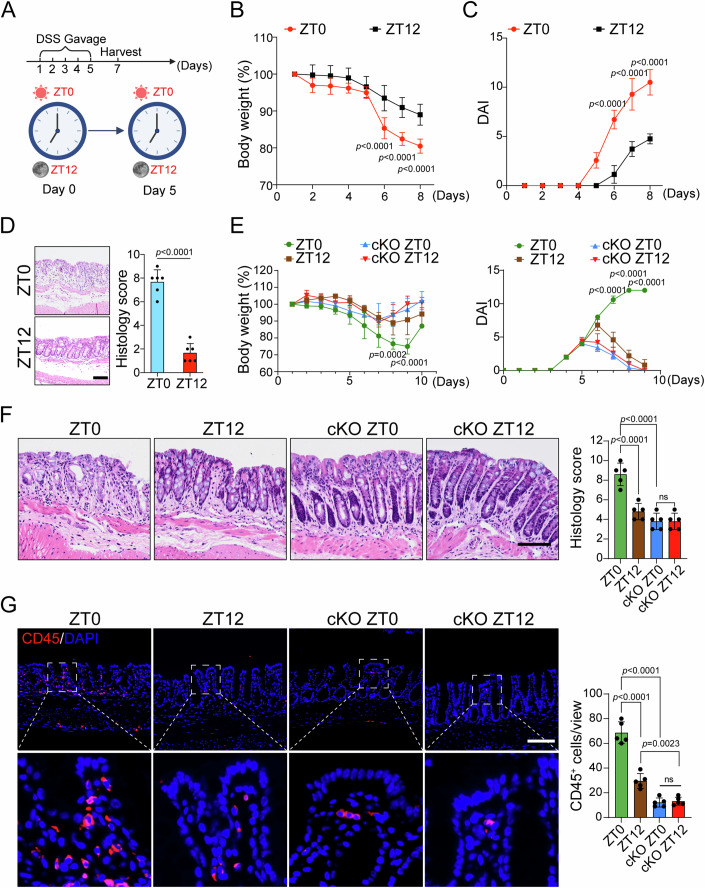
Mice in the active phase are resistant to DSS-induced colitis. (**A**) DSS gavage treatment to induce colitis in control mice and *Villin-CreERT2; Bmal1*^*fl/fl*^ mice (*Bmal1* cKO mice) during early resting (ZT0) and early active (ZT12) periods, respectively. (**B**, **C**) Body weight change and disease active index (DAI) scores of control mice after DSS gavage treatment at ZT0 or ZT12. *n* = 7 mice for each group. (**D**) Representative H&E stanning images of the distal colon sections, and histological scores were obtained for control mice after DSS gavage treatment at ZT0 or ZT12. *n* = 6 mice for each group at each time point. Scale bar: 100 µm. (**E**) Body weight change (left) and DAI scores (right) of control mice and *Bmal1* cKO mice after DSS gavage treatment at ZT0 or ZT12. *n* = 5 mice for each group. (**F**) Representative H&E stanning images of the distal colon sections, and histological scores were obtained for control mice and *Bmal1* cKO mice after DSS gavage treatment at ZT0 or ZT12. *n* = 5 mice for each group at each time point. Scale bar: 100 µm. (**G**) IF staining for CD45 (left) and quantification of CD45^+^ cells (right) in the distal colon sections from control mice and *Bmal1* cKO mice after DSS gavage treatment at ZT0 or ZT12. *n* = 5 mice for each group at each time point. Scale bar: 100 µm. Data information: Data are presented as mean ± SD. The data were analyzed by two-tailed Student’s t-test (**D**), one-way ANOVA with Tukey’s multiple comparisons test (**F**, **G**) and two-way ANOVA with Tukey’s multiple comparisons test (**B**, **C**, **E**). The exact *P* values are displayed.

Circadian rhythms are intrinsically approximate 24-h cyclic oscillations. To evaluate the effect of the circadian clock on intestinal inflammation, we generated a mouse model of acute colitis in the active (zeitgeber time [ZT]12 ~ ZT24) or resting (ZT0 ~ ZT12) periods. Wild-type mice were treated with DSS by gavage at the early resting time-point (ZT0) and the early active time-point (ZT12) for five consecutive days, respectively (Fig. 1A). We observed that compared to mice treated with DSS at the early active time-point, mice with DSS treatment at the early resting time displayed a more severe colitis, as evidenced by increased body weight loss (Fig. 1B), higher clinical disease activity index (DAI) scores (Fig. 1C), more severe disruption of colonic mucosal barrier, and higher histological scores (Fig. 1D). These results suggest that mice in the active phase are more resistant to DSS-induced gut colitis.

### *Bmal1* deficiency in the intestinal epithelium reduces DSS-induced colitis

To investigate the mechanism of how mice in the active phase are resistant to colitis, we assessed the expression pattern of the core circadian genes (*Bmal1*, *Clock*, *Per1*, *Per2*, and *Per3*) in control colonic crypts and found that most those genes exhibited an oscillatory expression pattern throughout the day (Fig. EV1A). Notably, *Bmal1* displayed its peak expression at the early resting time-point (ZT0) and reached its minimum at the early active time-point (ZT12), indicating that the dynamic expression of *Bmal1* might be involved in the resistance to colitis in the active phase.

**Figure EV1:**

Dynamic expression of circadian clock genes in the colonic epithelium. (**A**) RT-qPCR analysis of mRNA levels of circadian genes in the colonic crypts of control and *Bmal1* cKO mice. The colonic epithelia were collected at 3-h intervals over a 24-h period in a day. n = 3 mice for each group. Shaded areas represent mice in the dark. (**B**) After consecutive daily tamoxifen injection for 5 days to induce specific knockout of *Bma1* in the mouse intestinal crypts, tissues were harvested 7 days later for immunoblotting to examine BMAL1 and GAPDH protein levels. (**C**) Immunohistochemical analysis of BMAL1 in intestine sections from control and *Bmal1* cKO mice. Scale bar: 100 µm. Data information: Data are presented as mean ± SD. The rhythmicity of the oscillating pattern was measured by the JTK cycle through the MetaCycle R package. With the settings of Period = 24 h and adj.*p* < 0.05, expression patterns were then defined as rhythmic.

We then investigated the role of *Bmal1* in colitis by generating inducible intestinal epithelium-specific *Bmal1* knockout (cKO) mice (*Villin-CreERT2;Bmal1*^*fl/fl*^*)*, by intraperitoneal injection of tamoxifen for five consecutive days. *Bmal1* expression in intestinal epithelium was totally abolished in the knockout mice, which is confirmed by immunoblot analysis and immunohistochemistry (Fig. EV1A–C). Interestingly, other circadian genes (*Per1* and *Per2*) lost their oscillatory expression upon *Bmal1* depletion (Fig. EV1A), indicating a core role of *Bmal1* in modulating circadian rhythms in the intestinal epithelium. However, H&E (hematoxylin and eosin) staining showed that *Bmal1* depletion didn’t disrupt intestinal barrier structure (Fig. EV2A). Interestingly, the villus length of the proximal small intestine and the crypt length of the distal large intestine were increased in *Bmal1* cKO mice (Fig. EV2B). We then assessed whether *Bmal1* deletion affected the cell composition. Immunofluorescence staining showed no significant alterations in the distribution and quantity of KI67^+^ proliferating cells, OLFM4^+^ stem cells, LYZ^+^ Paneth cells, goblet cells, and CHGA^+^ enteroendocrine cells (Fig. EV2C–G). These results suggest that *Bmal1* deficiency has no effect on the self-renewal and differentiation of intestinal epithelial cells.

**Figure EV2:**

Ablation of *Bmal1* in the colonic epithelium does not affect the renewal and differentiation of colonic epithelial cells. (**A**) Histological images of the small intestine (SI) and large intestine (LI) from control and *Bmal1* cKO mice. Scale bar: 100 µm. (**B**) Histological images and quantification of villus length (top, left) and crypt length of the proximal small intestine (top, right) and distal large intestine (bottom) from control and *Bmal1* cKO mice. n = 6 mice for each group. Scale bar: 100 µm. (**C**) Immunofluorescence staining (left) of KI67^+^ TA cells in the SI and LI from control and *Bmal1* cKO mice. Quantification (right) of KI67^+^ TA cells in the SI (top) and LI (bottom) from control and *Bmal1* cKO mice. Epithelial cells were stained by E-cadherin. n = 6 mice for each group. Scale bar: 100 µm. (**D**, **E**) IF staining for OLFM4^+^ ISCs and LYZ^+^ Paneth cells (left) and quantification results of OLFM4^+^ and LYZ^+^ cells (right) in the small intestinal from control and *Bmal1* cKO mice. n = 6 mice for each group. Scale bar: 100 µm. (**F**) Alcain blue staining of goblet cells in the SI and LI from control and *Bmal1* cKO mice. Quantification (right) of goblet cells in the SI (top) and LI (bottom) from control and *Bmal1* cKO mice. n = 6 mice for each group. Scale bar: 100 µm. (**G**) IF staining (left) of CHGA^+^ enteroendocrine cells in the SI and LI from control and *Bmal1* cKO mice. Quantification (right) of CHGA^+^ cells in the SI (top) and LI (bottom) from control and *Bmal1* cKO mice. Epithelial cells were stained by E-cadherin. n = 6 mice for each group. Scale bar: 100 µm. Data information: Data are presented as mean ± SD. The data were analyzed by two-tailed Student’s t-test (**B**–**G**). The exact *P* values are displayed. ns, no significance. Source data are available online for this figure.

To investigate the role of *Bmal1* in intestinal inflammation, both control and *Bmal1* cKO mice were treated with DSS at ZT0 or ZT12, respectively (Fig. 1A). Surprisingly, different from control mice, *Bmal1* cKO mice developed less severity of colitis even treated with DSS at the resting time ZT0 (Fig. 1E–G). These mice showed no significant differences in body weight loss, DAI scores, and histological scores, compared with the animals (control or *Bmal1* cKO) treated at the active phase (Fig. 1E,F). Immunofluorescence staining of CD45^+^ immune cells showed reduced immune cell infiltration in *Bmal1* cKO mice compared to the control mice, no matter in ZT0-treated mice or ZT12-treated mice (Fig. 1G). These results suggest that *Bmal1* deficiency renders mice resistant to DSS-induced colitis.

**Figure 2:**
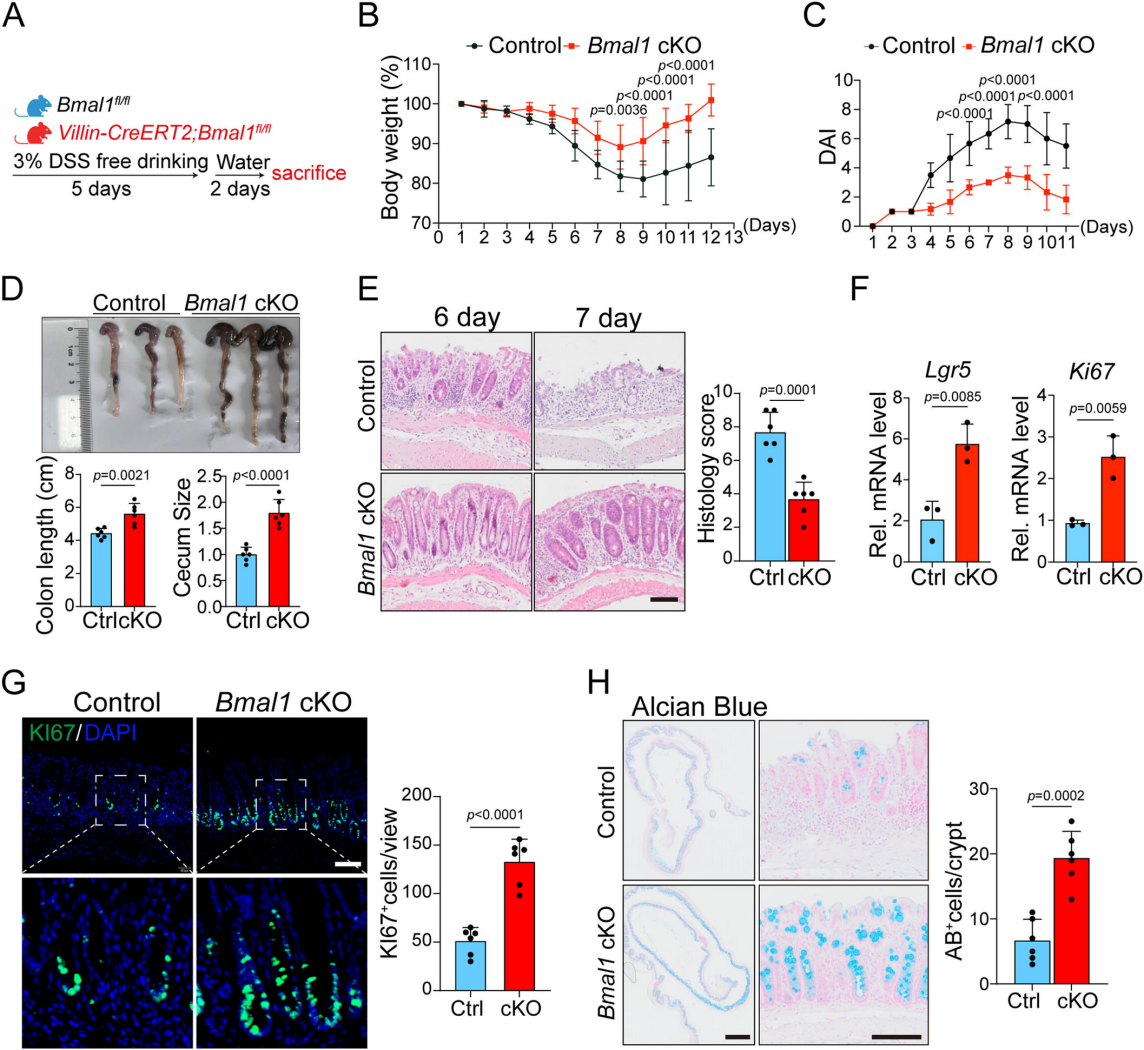
*Bmal1* deficiency in the intestinal epithelium attenuates DSS-induced colitis. (**A**) Schematic diagram showing the DSS-induced colitis model in control and *Bmal1* cKO mice. (**B**) Body weight change of control and *Bmal1* cKO mice after DSS free drinking treatment, with 7 mice for each group at each time point. *n* = 7/8 mice for each group. (**C**) DAI scores of control and *Bmal1* cKO mice after DSS free drinking treatment. *n* = 6 mice for each group. (**D**) Colon length and cecum size of control and *Bmal1* cKO mice were assessed after 3% DSS drinking for 5 days, followed by a switch to normal water drinking for 2 days. *n* = 6 mice for each group. (**E**) Representative H&E stanning images of the distal colon sections, and histological scores were obtained for control and *Bmal1* cKO mice at day 7 following DSS treatment. *n* = 6 mice for each group. Scale bar: 100 µm. (**F**) RT-qPCR analysis for the mRNA expression of *Lgr5* and *Ki67* in the colonic crypts of control and *Bmal1* cKO mice at day 7 following DSS treatment. *n* = 3 mice for each group. (**G**) IF stanning (left) and quantification (right) of KI67^+^ TA cells in the colonic epithelium from control and *Bmal1* cKO mice at day 7 following DSS treatment. *n* = 6 mice for each group. Scale bar: 100 µm. (**H**) Alcian blue (AB) stanning (left) of goblet cells quantification (right) of AB^+^ cells from control and *Bmal1* cKO mice at day 7 following DSS treatment. *n* = 6 mice for each group. Scale bar: 2 mm (left); 100 µm (right). Data information: Data are presented as mean ± SD. The data were analyzed by two-tailed Student’s t-test (**D**–**H**) and two-way ANOVA with Tukey’s multiple comparisons test (**B**, **C**). The exact *P* values are displayed.

To better assess the role of *Bmal1* in intestinal inflammation, we introduced the experimental colitis in control or *Bmal1-*cKO mice through unrestricted access to 3% DSS solution (Fig. 2A). Consistently, we observed a much milder colitis phenotype in cKO mice, characterized by significantly lower decrease in body weight, longer colon length, larger cecum volume and smaller DAI scores, compared to the control group (Fig. 2B–D). Histological analysis also revealed that the colonic mucosal barrier in cKO mice retained a more intact structure and exhibited lower histological scores (Fig. 2E). Furthermore, increased expression of the stem cell marker *Lgr5* and proliferative marker gene *Ki67* was also observed in *Bmal1-*depleted colonic crypts (Fig. 2F). Moreover, immunofluorescence staining of KI67^+^ proliferating cells and Alcian blue staining of goblet cells showed an increase in *Bmal1*-defficency colonic epithelium (Fig. 2G,H), indicating that a functional integrity of the intestinal epithelium was reserved in *Bmal1*-cKO mice.

### *Bmal1* transcriptionally activates apoptotic genes in DSS-induced colitis

**Figure 3:**
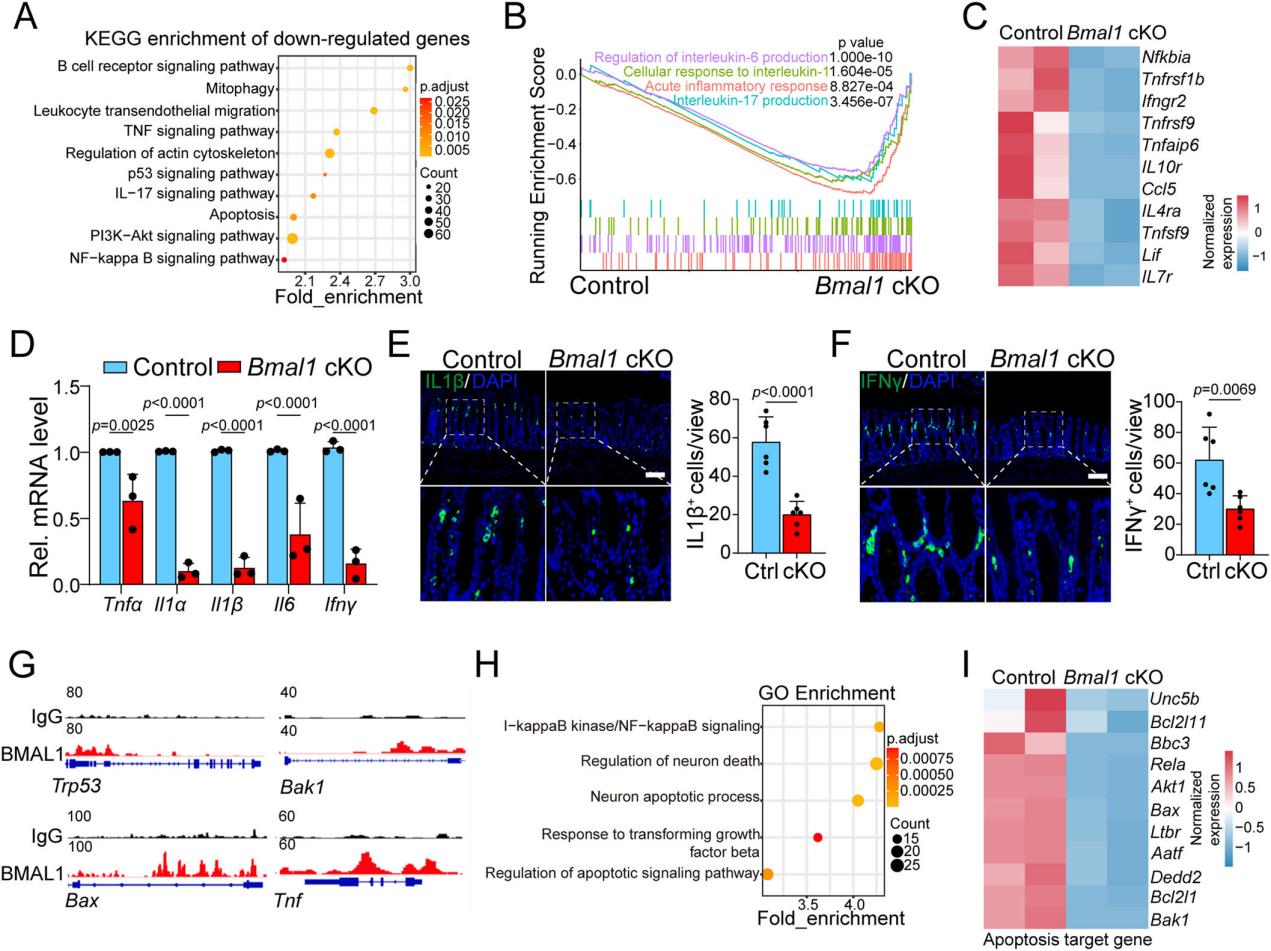
*Bmal1* transcriptionally activates apoptotic genes in DSS-induced colitis. (**A**) KEGG enrichment of downregulated genes in the colonic crypts of *Bmal1* cKO mice. The tissues were collected from control and *Bmal1* cKO mice at day 7 following DSS treatment. *n* = 2 mice for each group. (**B**) The inflammatory response and immune-regulating related genes were analyzed using GSEA in DSS-treated control and *Bmal1* cKO mice at day 7 following DSS treatment. n = 2 mice for each group. (**C**) Heatmap showing the expression of the indicated inflammatory cytokines in the colonic crypts from control and *Bmal1* cKO mice at day 7 following DSS treatment. *n* = 2 mice for each group. (**D**) RT-qPCR analysis of the mRNA expression of pro-inflammatory cytokines in the colonic crypts from control and *Bmal1* cKO mice at day 7 following DSS treatment. *n* = 3 mice for each group. (**E**, **F**) IF staining for IL1β^+^ and INFγ^+^ cells and quantification results in the distal colon sections from control and *Bmal1* cKO mice at day 7 following DSS treatment. *n* = 6 mice for each group. Scale bar: 100 µm. (**G**) Genomic views of BMAL1 CUT&Tag-seq assay enrichment at the promoters of the apoptosis-related genes in colonic organoids from control mice. (**H**) GO enrichment of overlapped genes between CUT&Tag-seq assay and downregulated genes in RNA-seq in colonic crypts from control and *Bmal1* cKO mice at day 7 following DSS treatment. (**I**) Heatmap showing apoptosis-related gene expression after downregulation of genes in RNA-seq from *Bmal1* cKO mice at day 7 following DSS treatment overlapped with CUT&Tag-seq genes from control colonic organoids. Data information: Data are presented as mean ± SD. The data were analyzed by two-tailed Student’s t-test (**E**, **F**) and two-way ANOVA with Tukey’s multiple comparisons test (**D**). GO and KEGG pathway enrichment analysis in the hypergeometric test and GSEA used the random permutation test. The exact *P* values are displayed.

To further reveal the underlying mechanism by which *Bmal1* governs the progression of colitis, we performed bulk RNA sequencing in colonic epithelial cells isolated from control and *Bmal1* cKO mice at 7 days post DSS treatment (Dataset EV1). Gene Ontology (GO) enrichment and Kyoto Encyclopedia of Genes and Genomes (KEGG) pathway enrichment analyses showed that the downregulated genes from *Bmal1* cKO were enriched in a series of cellular processing, including inflammatory responses, apoptosis and p53 signaling (Figs. 3A and EV3A). Conversely, the upregulated genes were associated with autophagy, protein regulation, and metabolism-related processes (Fig. EV3A,B). Gene set enrichment analysis (GSEA) showed a significant repression of acute inflammation response, cellular response to interlukin-1, interlukin-17 production, and regulation of interlukin-6 production in *Bmal1* cKO group (Fig. 3B). As expected, the expression of genes encoding pro-inflammatory cytokines and chemokines, such as *Tnfα*, *Il1α*, *Il1β*, *Il6*, and *Ifnγ*, exhibited a remarkable decrease in *Bmal1* cKO mice following DSS treatment (Fig. 3C,D). Moreover, the proportion of inflamed cytokines (IL-1β), infiltrating immune cells (CD45^+^ cells) and macrophages (F4/80^+^ cells), and IFNγ expression in the inflamed area was decreased (Figs. 3E,F and EV3C,D). Collectively, these results indicate that *Bmal1* cKO mice exhibited less inflammatory and immune response in colon, thus more resistant to colitis.

**Figure EV3:**

Ablation of *Bmal1* in the intestinal epithelium is accompanied by reduced inflammation and immune response. (**A**, **B**) GO enrichment (**A**) of upregulated genes and downregulated genes in the colonic crypts of *Bmal1* cKO mice and KEGG enrichment (**B**) of upregulated genes in the colonic crypts. The tissues were collected from control and *Bmal1* cKO mice, respectively, after 3% DSS drinking for 5 days, followed by a switch to normal water drinking for 2 days. (**C**, **D**) IF staining for F4/80 (**C**) and CD45 (**D**) and quantification of F4/80^+^ and CD45^+^ cells (right) in the distal colon sections from control and *Bmal1* cKO mice. n = 6 mice for each group. Scale bar: 100 µm. (**E**) Genomic views of BMAL1 CUT&Tag-seq assay enrichment at the promoters of the apoptosis-related genes in colonic organoids from control mice. (**F**) Venn diagram shows the overlapped genes between anti-BMAL1 CUT&Tag-seq assay and downregulated genes by RNA-seq in *Bmal1* cKO mice. The colonic organoids from wild-type mice were collected for CUT&Tag-seq assay. The tissues were collected from *Bmal1* cKO mice after 3% DSS drinking for 5 days, followed by a switch to normal water drinking for 2 days for RNA-seq. (**G**) GSEA of apoptosis gene sets enriched with decreased genes in colonic crypts from *Bmal1* cKO mice. Data information: Data are presented as mean ± SD. The data were analyzed by two-tailed Student’s t-test (**C**, **D**). The exact *P* values are displayed.

Next, we identified the downstream genes transcriptionally regulated by BMAL1. We performed CUT&Tag-seq assay in colonic organoids from wild-type mice to identify BMAL1-bound genes (Dataset EV2). BMAL1 directly bound to the promoters of several apoptosis-related genes, including *p53*, *Bax* and *Bak1* (Figs. 3G and EV3E). By overlapping with the downregulated genes in bulk RNA-seq data, we identified 495 genes as the potential *Bmal1*-regulated genes (Fig. EV3F). GO analysis revealed that the majority of these genes were enriched in cell death and the apoptotic pathway (Fig. 3H). Consistently, genes involved in apoptotic signaling were greatly downregulated upon the absence of *Bmal1* (Figs. 3I and EV3G). These results indicate that BMAL1 can transcriptionally activate the expression of apoptosis-related genes in the DSS-treated intestine.

### *Bmal1* cKO reduces cell apoptosis in the intestinal epithelium

**Figure 4:**
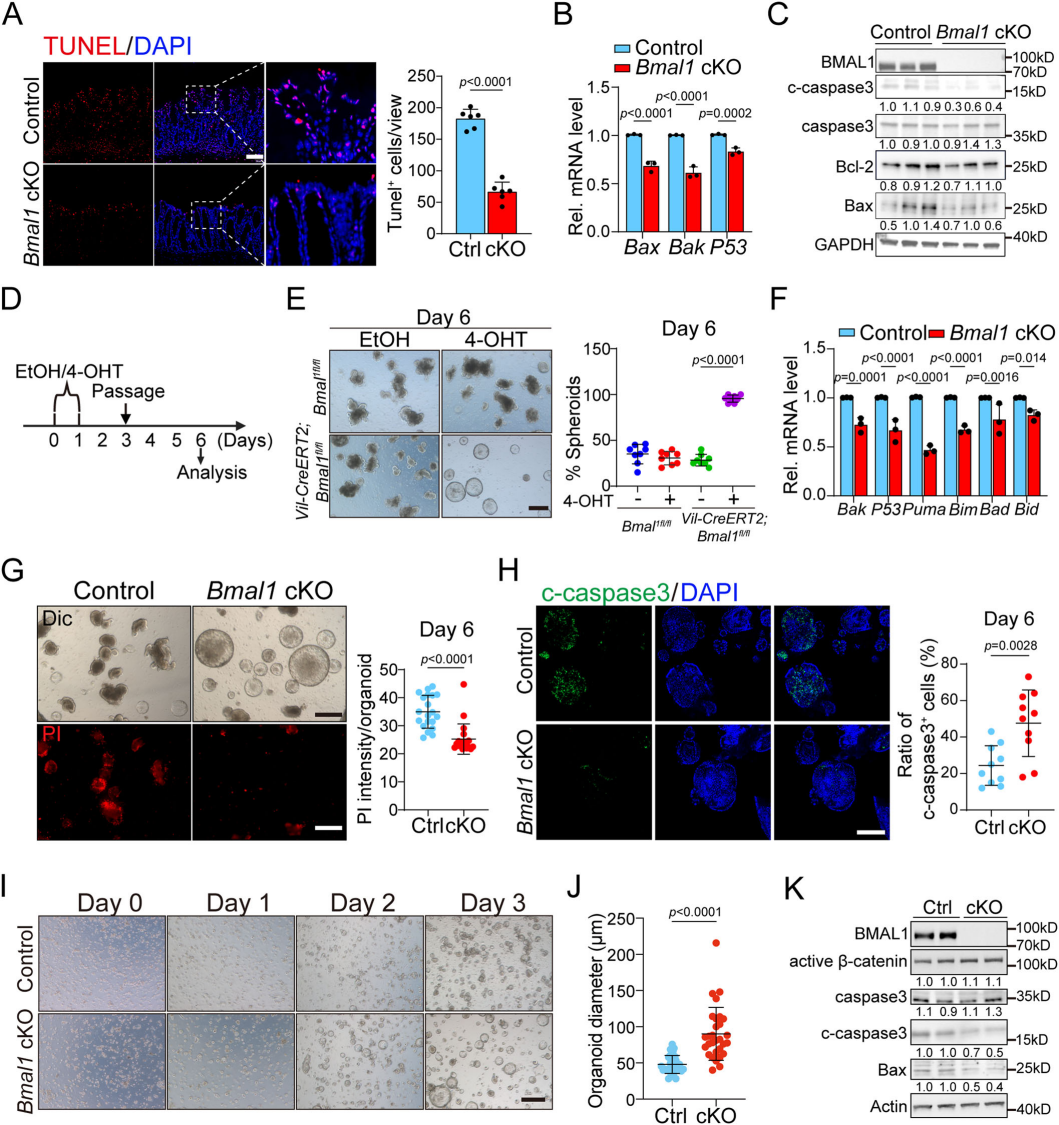
Cell apoptosis is attenuated in *Bmal1*-deficient intestinal epithelium. (**A**) TUNEL staining was performed (left), and the TUNEL^+^ cells were quantified (right) in colonic sections obtained from control and *Bmal1* cKO mice after DSS treatment. *n* = 6 mice for each group. Scale bar: 100 µm. (**B**) RT-qPCR analysis of the mRNA expression of *Bak1*, *Bax,* and *p53* in the colonic crypts from control and *Bmal1* cKO mice. *n* = 3 mice for each group. (**C**) Western blotting for BMAL1, c-caspase 3, caspase 3, Bcl-2, Bax in the colonic crypts of control and *Bmal1* cKO mice. GAPDH was used as loading control. *n* = 3 mice for each group. (**D**) Schematic showing *Bmal1* cKO in colonic organoids by adding 4-OHT for 24 h. Organoids were passaged at day 3 and harvested or photographed on day 6 for subsequent analysis. (**E**) Organoids derived from the crypts of *Bmal1*^*fl/fl*^ and *Villin-CreERT2; Bmal1*^*fl/fl*^ mice were treated with Et-OH or 4-OHT for 24 h. Images were taken at day 6, and the spheroid organoid ratio (right) was counted from 8 fields of view from three independent experiments. Scale bar: 400 μm. (**F**) RT-qPCR analysis of the mRNA expression of *Bak*, *P53, Puma, Bim, Bad,* and *Bid* from control or *Bmal1* cKO organoids at day 6. *n* = 3 biological replicates. (**G**) Representative brightfield (top), PI staining (bottom) and PI intensity quantification (right) from control and *Bmal1* cKO colonic organoids at day 6. *n* = 20 organoids for each group. Scale bar: 400 µm. (**H**) IF staining of c-caspase 3 from control and *Bmal1* cKO colonic organoids at day 6. *n* = 10 organoids for each group. Scale bar: 200 µm. (**I**) Representative morphology of control and *Bmal1* cKO organoids starting at single cells at indicated times. Scale bar: 400 μm. (**J**) Organoid diameter of control and *Bmal1* cKO organoids at day 3 starting at single cells. *n* = 30 organoids for each group. (**K**) Immunoblots of BMAL1, active β-catenin, caspase 3, c-caspase 3, and Bax protein expression in organoids derived from control and *Bmal1* cKO organoids at day 3 starting at single cells. Actin was used as loading control. Data information: Data are presented as mean ± SD. The data were analyzed by two-tailed Student’s t-test (**A**, **G**, **H**, **J**), one-way ANOVA with Tukey’s multiple comparisons test (**E**) and two-way ANOVA with Tukey’s multiple comparisons test (**B**, **F**). The exact *P* values are displayed.

**Figure EV4:**
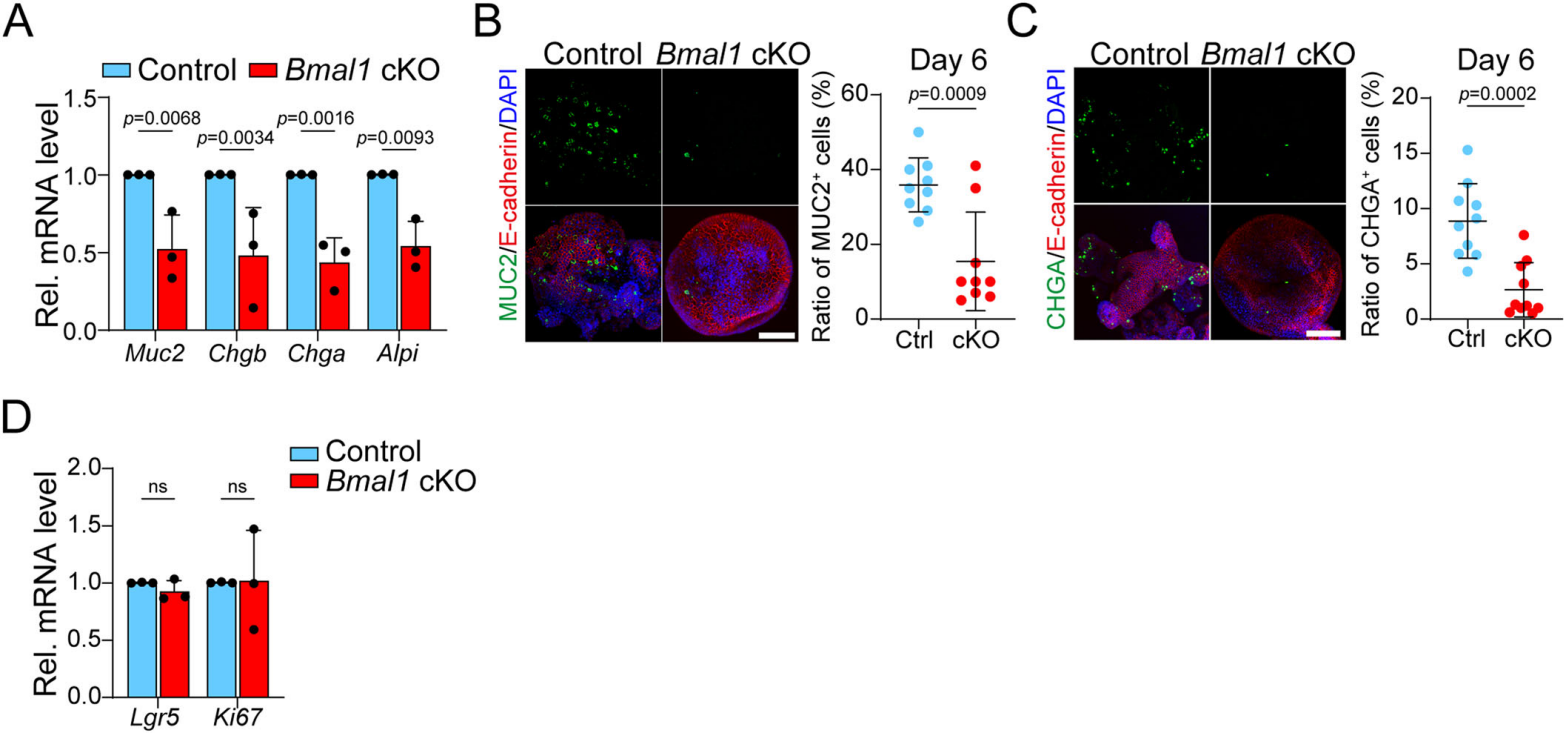
Ablation of *Bmal1* suppresses differentiation but does not affect stemness and proliferation in vitro. (**A**) RT-qPCR analysis of the mRNA expression of *Muc2*, *Chgb, Chga* and *Alpi* from control or *Bmal1* cKO organoids on day 6. n = 3 biological replicates. (**B**, **C**) Immunofluorescence staining for MUC2 (**B**) and CHGA (**C**) and quantification of MUC2^+^ and CHGA^+^ cells (right) from control or *Bmal1* cKO organoids. n = 9/10 organoids for each group. Scale bar: 100 µm. (**D**) RT-qPCR analysis for the mRNA expression of *Lgr5* and *Ki67* in control and *Bmal1* cKO organoid*s* at day 3 starting at single cells. n = 3 biological replicates. Data information: Data are presented as mean ± SD with statistical analyses determined by two-tailed Student’s t-test (**B**, **C**) and two-way ANOVA with Tukey’s multiple comparisons test (**A**, **D**). The exact *P* values are displayed. ns, no significance.

We further assessed the effect of *Bmal1* on cell apoptosis and found that TUNEL^+^ cells were decreased upon *Bmal1* depletion (Fig. 4A). In agreement with this, the expression of core apoptotic genes, *p53*, *Bax,* and *Bak1*, as well as the level of cleaved-caspase 3 (c-caspase 3), were downregulated upon *Bmal1* ablation in the colonic epithelium (Fig. 4B,C). These results reveal that *Bmal1* deficiency reduces cell apoptosis in the DSS-treated intestinal epithelium. These observations were confirmed in ex vivo colonic organoids derived from *Villin-CreERT2;Bmal1*^*fl/fl*^ mice. Deletion of *Bmal1* in organoids was achieved by treatment of 4-hydroxytamoxifen (4-OHT) (Fig. 4D). *Bmal1* cKO organoids exhibited spheroid morphology (Fig. 4E), potentially attributable to the suppression of cell differentiation, including MUC2^+^ goblet cells and CHGA^+^ enteroendocrine cells (Fig. EV4A–C). We also found the expression of apoptotic genes, such as *Bak*, *P53*, *Puma*, *Bim*, *Bad* and *Bid*, exhibited a remarkable decrease in *Bmal1* cKO organoids (Fig. 4F). Consistently, dead cells, shown by propidium iodide (PI) staining, and c-caspase 3^+^ cells were decreased upon *Bmal1* cKO in colonic organoids (Fig. 4G,H). To further assess the impact of *Bmal1* on organoid growth, we performed single cell cultures digested from the control or *Bmal1* cKO organoids and found that *Bmal1*-depleted organoids grew faster (Fig. 4I,J). Of note, the improved organoid growth might attribute to decreased cell death and increased Wnt signaling but not cell proliferation, as indicated by reduced c-caspase 3 and Bax, increased active β-catenin levels, unchanged *Lgr5* or *Ki67* expression (Figs. 4K and EV4D). These results indicate the role of *Bmal1* in colitis-associated cell apoptosis in the intestinal epithelium.

### Cell apoptosis exhibits a circadian rhythm in the intestinal epithelium

**Figure 5:**
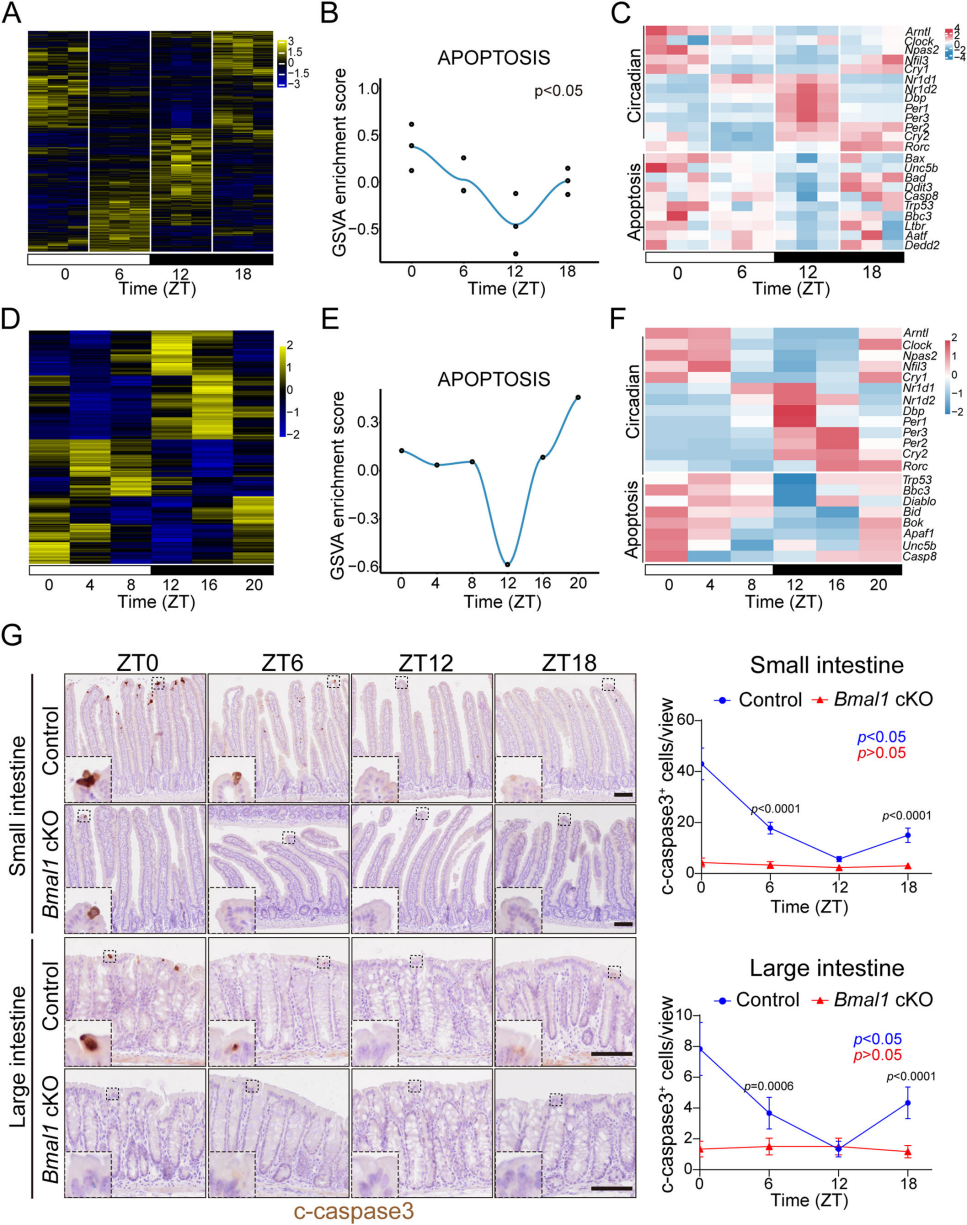
Cell apoptosis occurs in a circadian rhythm-dependent manner in the intestinal epithelium. (**A**) Heatmap showing gene expression ordered by MetaCycle phase to depict significant circadian genes in colonic crypts from wild-type mice. *n* = 3 mice for each group. (**B**) GSVA score for apoptosis gene sets enriched in colonic crypts obtained from the wild-type mice at indicated time point. Individual points represent the enrichment score for each sample. *n* = 3 mice for each group. (**C**) Heatmap showing the expression of circadian genes and apoptosis-related genes in the colonic crypts from wild-type mice at indicated time point. *n* = 3 mice for each group. (**D**) Heatmap showing gene expression ordered by MetaCycle phase to depict significant circadian genes in the small intestinal epithelium from wild-type mice using the dataset GSE100339 (Wang et al, 2017; Data ref: Wang et al, 2017). (**E**, **F**) GSVA score for apoptosis gene sets enriched (**E**) and heatmap (**F**) showing the expression of circadian genes and apoptosis-related genes in small intestinal epithelium from wild-type mice at indicated time point using the dataset GSE100339. (**G**) Immunohistochemical analysis of c-caspase 3 (left) and quantification (right) in proximal small intestine (top) and distal large (bottom) intestine sections from control mice and *Bmal1* cKO mice at ZT0, ZT6, ZT12, and ZT18. Scale bar: 100 µm. *n* = 6 mice for each group at indicated time point. Data information: Data are presented as mean ± SD. The rhythmicity of the oscillating pattern was measured by the JTK cycle through the MetaCycle R package. With the settings of Period = 24 h and adj.*p* < 0.05, expression patterns were then defined as rhythmic. Two-way ANOVA with Tukey’s multiple comparisons test was also used (**G**). The exact *P* values are displayed.

The results above prompted us to speculate that cell apoptosis might be rhythmic in the homeostatic intestinal epithelium. The colonic crypts were collected from wild-type mice sacrificed at ZT0, ZT6, ZT12, and ZT18 for RNA-sequencing and transcriptomes were analyzed by MetaCycle (De Los Santos et al, 2020; Wu et al, 2016) (Dataset EV3). Heatmap showed that a portion of genes exhibited a rhythmic expression pattern (Fig. 5A). Gene Set Variation Analysis (GSVA) showed that the transcript levels of the apoptosis-related genes exhibited rhythmic oscillations, with the higher level at ZT0-ZT6 and lower level at ZT12 (Fig. 5B). The expression of the core apoptotic genes, such as *Bax* and *p53*, had a similar pattern (Fig. 5C). Analysis of the public transcriptome data (Wang et al, 2017; Data ref: Wang et al, 2017) in the small intestinal epithelium also revealed that the expression of a portion of genes, especially apoptosis-related genes, exhibited a rhythmic pattern (Fig. 5D–F). Consistently, the c-caspase 3 signals also had a periodic pattern with the highest level at ZT0 and lowest at ZT12 in the proximal small intestine and distal large intestine (Fig. 5G). Furthermore, *Bmal1* cKO led to reduced c-caspase 3 signals in the intestinal epithelium (Fig. 5G). These results indicate that cell apoptosis exhibits a *Bmal1*-dependent circadian rhythm pattern in the intestinal epithelium.

### SR9009 treatment at ZT0 alleviates DSS-induced colitis

**Figure 6:**
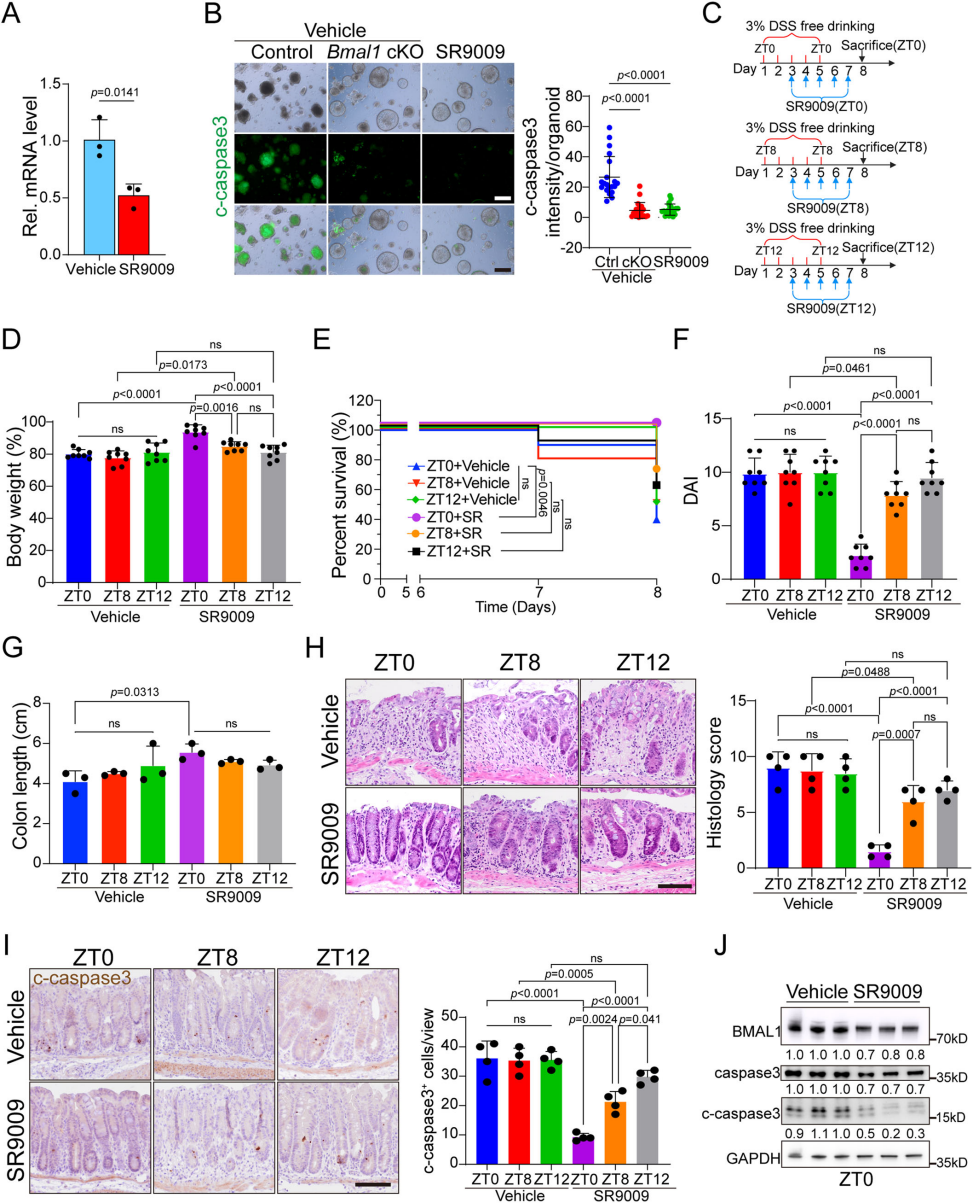
Inhibition of *Bmal1* expression at ZT0 has the best efficacy in DSS-induced colitis. (**A**) RT-qPCR analysis of the mRNA expression of *Bmal1* from control colonic organoids and SR9009-treated organoids. *n* = 3 biological replicates. (**B**) Representative brightfield (top), c-caspase 3 staining (bottom) and its intensity quantification (right) from control, *Bmal1* cKO and SR9009-treated colonic organoids. Scale bar: 400 µm. *n* = 20 organoids for each group. (**C**) Schematic diagram showing SR9009 administration during colitis induction. Wild-type mice were given DSS solution ad libitum for five days at ZT0, ZT8, and ZT12, respectively, and switched to water at ZT0, ZT8, and ZT12 on the fifth day to ensure that each group of mice consumed DSS drinking water for five days. SR9009 (50 mg/kg) was administered intraperitoneally at single injection daily for 5 consecutive days at ZT0, ZT8, and ZT12, respectively, starting on the third day of DSS-induced colitis in mice. Colon tissues were subsequently collected at day 8. Control group intraperitoneal injection of vehicle. (**D**–**F**) Body weight change (**D**), survival curve (**E**), and DAI scores (**F**) of wild-type mice after DSS treatment. SR9009 (50 mg/kg) was administered intraperitoneally once daily for 5 consecutive days at ZT0, ZT8, and ZT12, respectively, starting on the third day of DSS-induced colitis in mice. Mice were injected intraperitoneally with the vehicle as a control group at ZT0, ZT8 and ZT12. *n* = 8/10 mice for each group. (**G**) Colon length quantification from six groups of mice. Colon tissues were collected at day 8. *n* = 3 mice for each group. Scale bar: 100 µm. (**H**) Representative H&E stanning images of the distal colon sections, and histological scores were obtained from 6 groups of mice. Colon tissues were collected at day 8. *n* = 4 mice for each group Scale bar: 100 µm. (**I**) Immunohistochemical analysis of c-caspase 3 (left) and quantification (right) in distal colon sections from 6 groups. Colon tissues were collected at day 8. Scale bar: 100 µm. *n* = 4 mice for each group. (**J**) Western blotting for BMAL1, c-caspase 3, caspase 3 in the colonic crypts of control and SR9009-treated mice after DSS treatment at ZT0. GAPDH was used as loading control. *n* = 3 mice for each group. Data information: Data are presented as mean ± SD, analyzed by two-tailed Student’s t-test (**A**), one-way ANOVA with Tukey’s multiple comparisons test (**B**, **D**, **F**–**I**) and one-sided log-rank (**E**). The exact *P* values are displayed.

**Figure EV5:**
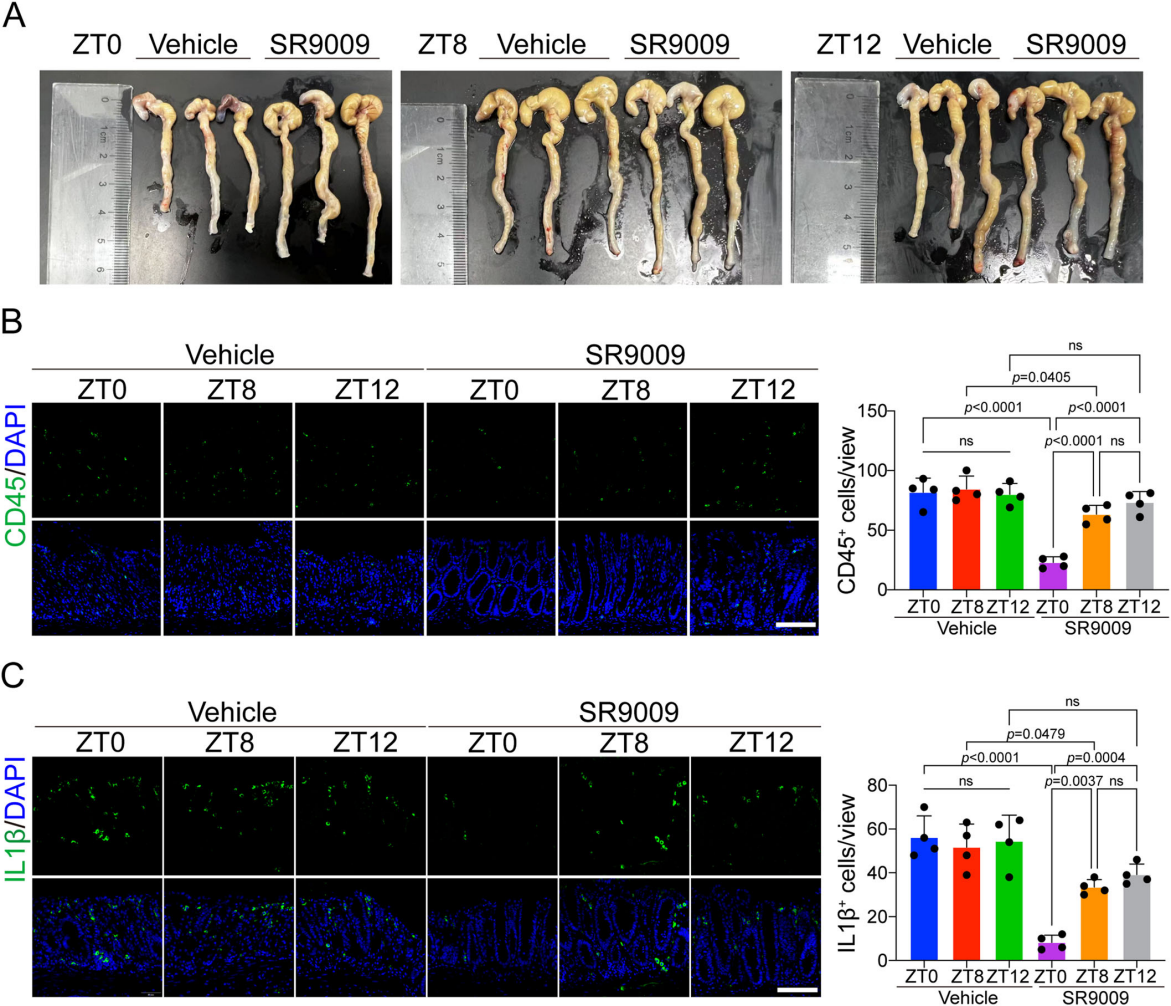
Mice treated with SR9009 at ZT0 have less immune cell infiltration. (**A**) Colon length from six groups of mice. (**B**, **C**) Immunofluorescence staining for CD45 (**B**) and IL1β (**C**) and quantification of CD45^+^ and IL1β^+^ cells (right) in the distal colon sections from six group of mice. n = 4 mice for each group. Scale bar: 100 µm. Data information: Data are presented as mean ± SD, analyzed by one-way ANOVA with Tukey’s multiple comparisons test (**B**, **C**). The exact *P* values are displayed. ns, no significance.

The small molecule SR9009 is an agonist of REV-ERBα/β that can reduce *Bmal1* expression (Zhuang et al, 2019). It has been shown to be effective in alleviating DSS-induced colitis and high-fat diet induced obesity in mice (Wang et al, 2018; Yu et al, 2021). We first investigated the effects of SR9009 in vitro by treating organoids with the compound and observed a decrease of *Bmal1* expression (Fig. 6A). C-caspase 3 staining revealed that both SR9009-treatment and *Bmal1* cKO decreased cell apoptosis (Fig. 6B). As DSS-induced colitis and cell apoptosis exhibited a *Bmal1*-dependent circadian rhythm pattern, we investigated the optimal timing of SR9009 administration in colitis treatment. Wild-type mice were given DSS solution ad libitum for 5 days at ZT0, ZT8, and ZT12, respectively, and switched to water at ZT0, ZT8, and ZT12 on the fifth day to ensure that each group of mice consumed DSS drinking water for 5 days (Fig. 6C). Starting at day 3, SR9009 was administered via intraperitoneal injection to DSS-treated mice at ZT0, ZT8, and ZT12 for 5 consecutive days (Fig. 6C). We found mice treated with SR9009 at ZT0 exhibited superior therapeutic outcomes, evidenced by less body weight loss, lower DAI scores, higher survival rates and longer colon lengths, compared to those treated at other time points, while treatment at ZT12 being the least effective (Figs. 6D–G and EV5A). Histochemical staining showed that SR9009 treatment at ZT0 had the lowest histological scores (Fig. 6H). Furthermore, fewer apoptotic cells and decreased BMAL1 and c-caspase 3 levels were detected in SR9009-treated mice at ZT0 (Fig. 6I,J). Consistently, mice treated at ZT0 had significantly reduced infiltration of immune CD45^+^ cells and lower expression levels of inflammatory protein IL-1β (Fig. EV5B,C). Collectively, these results indicate that SR9009 treatment at ZT0 has the best beneficial efficacy in DSS-induced colitis.

### BMAL1 expression is reduced in clinical UC tissue samples

**Figure EV6:**
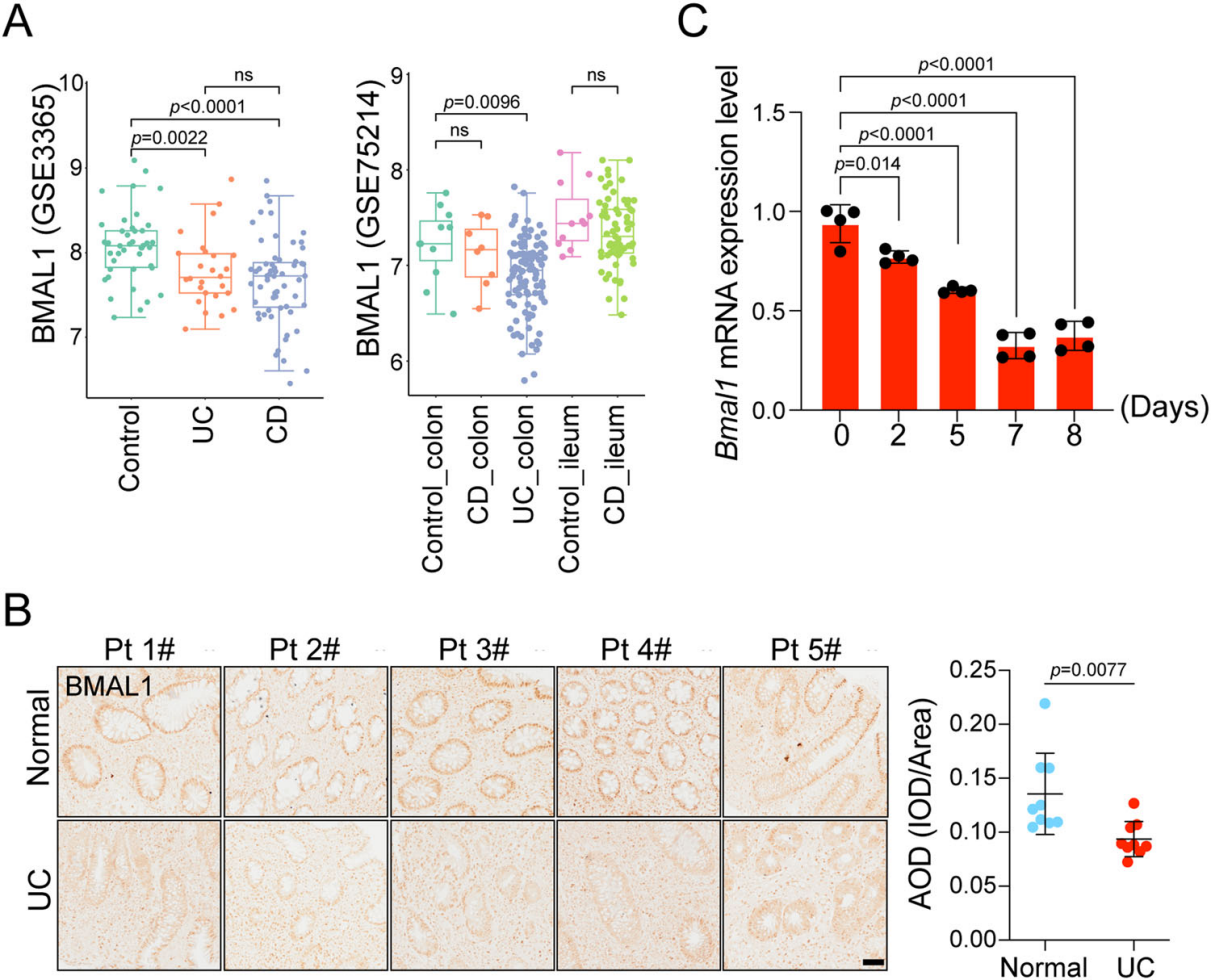
BMAL1 expression is reduced in clinical UC tissues. (**A**) Box plot showing mRNA expression of *BMAL1* in healthy and IBD patients using datasets GSE3365 (Burczynski et al, 2006; Data ref: Burczynski et al, 2006) and GSE75214 (Vancamelbeke et al, 2017; Data ref: Vancamelbeke et al, 2017). The central line represents the median. The box spans from the 25th to the 75th percentile. Whiskers extend to the smallest and largest values. All points are presented. GSE3365: Control, n = 42; UC, n = 26; CD, n = 5. GSE75214: Control_colon, n = 11; CD_colon, n = 8; UC_colon, n = 97; Control_ileum, n = 11; CD_ileum, n = 67. (**B**) Immunohistochemical analysis (left) of BMAL1 (CST, 14020S) in healthy and inflammatory regions of colonic epithelium from UC patients. Quantification of the IHC staining results by calculating the average option density (AOD) from normal and inflammatory regions of colonic epithelium from UC patients. n = 9 patient. Scale bar: 50 µm. (**C**) RT-qPCR of *Bmal1* mRNA expression in the colonic crypts of control and *Bmal1* cKO mice at different time points. n = 4 mice at different time points. Data information: Data are presented as mean ± SD, analyzed by two-tailed Student’s t-test (**B**), one-way ANOVA with Tukey’s multiple comparisons test (**C**). The exact *P* values are displayed. ns, no significance.

To examine the clinical relevance of our findings obtained from mice, we detected BMAL1 expression in clinical human UC tissue samples. By analyzing publicly available datasets (Burczynski et al, 2006; Data ref: Burczynski et al, 2006; Vancamelbeke et al, 2017; Data ref: Vancamelbeke et al, 2017), we found that the mRNA level of the *BMAL1* was lower in UC patients, compared to that in normal individuals (Fig. EV6A). Immunohistochemistry staining also confirmed that BMAL1 protein decreased in the inflammatory regions of colonic epithelium from UC patients, relative to the normal tissues (Fig. EV6B). Interestingly, the decreased BMAL1 expression in UC patients was consistent with the observation in DSS-induced colitis in mice in which *Bmal1* levels were gradually declined at different time points during the colitis progress (Fig. EV6C).

## Discussion

Various environmental triggers have been found to initiate and spread mucosal inflammation in susceptible IBD individuals (Khor et al, 2011). Sleep disruption and chronic fatigue are the major symptoms associated with IBD (Ranjbaran et al, 2007), and these symptoms are caused by disruption of circadian rhythms, which is widespread in modern people with irregular routines (Swanson et al, 2011). Consistently, circadian clock genes have been shown to play an important role in regulating digestive physiology and maintaining functions of gut barrier (Kyoko et al, 2014). In the present study, we surprisingly observed that mice in the active circadian phase are more resistant to DSS-induced colitis, and ablation of the circadian gene *Bmal1* renders the intestinal epithelium resistance to colitis.

Although inactivation of *Bmal1* in the intestinal epithelium had no significant effect on intestinal homeostasis, which is consistent with previous reports (Yu et al, 2021). However, we found that upon DSS treatment, *Bmal1* cKO mice exhibited a resistance to DSS-induced colitis, as evidenced by less weight loss, more intact epithelial barrier structure and function, as well as less infiltration of inflammatory factors and immune cells compared to control mice. This seems to contradict the findings of previous studies showing that mice with global knockout of the circadian genes *Bmal1*, *Per1/2*, or *Rev-erbβ* exhibit more severe symptoms in experimental colitis (Liu et al, 2021; Pagel et al, 2017; Taleb et al, 2022; Wang et al, 2018). Global knockout of circadian genes is not sufficient to characterize clock function in specific tissues as interference may come from other tissues, which may explain why different colitis phenotypes are observed in global knockout and intestinal epithelial-specific knockout of *Bmal1*. Global *Bmal1*-deficient mice also exhibit age-associated dilated cardiomyopathy, with dysfunction of left ventricular dilatation and contraction (Lefta et al, 2012). Adipocyte-specific deletion of *Bmal1* leads to obesity in mice for the disrupted food intake rhythm (Paschos et al, 2012), whereas intestinal epithelial-specific *Bmal1* deletion reduces high-fat diet-induced obesity (Yu et al, 2021). Deletion of *Bmal1* in Villin-Cre mice during the embryonic stage affects normal intestinal functions, such as rhythmicity of microbiota disruption, immune cell recruitment (Heddes et al, 2022). and is more susceptible to DSS-induced colitis (Jochum et al, 2023; Niu et al, 2024). In this study, we used CreER mice to exclude these potential effects of Cre constitutive knockout mice. Inactivation of *Bmal1* in the intestinal epithelium of adult mice had no significant effect on intestinal homeostasis but was more tolerant to DSS. These results indicate that the continuous loss of *Bmal1* during intestinal development and conditional loss under adult homeostasis lead to different consequences.

Excessive apoptosis of intestinal epithelial cells attributes to the development of IBD (Gunther et al, 2013), by disrupting the intestinal epithelial barrier, allowing the invasion of bacterial into the submucosal layer and promoting the host inflammatory response and ROS burst (Wan et al, 2022). We found that BMAL1 directly binds to the promoter regions of many apoptosis genes, such as *Bak1* and *Bax*, to enhance their transcription. Previous studies have reported that ~3–10% of transcripts exhibit circadian oscillations in mouse and human organoids cultured in vitro (Rosselot et al, 2022). Furthermore, we found that in homeostatic intestinal epithelium, cell apoptosis displays a circadian rhythm, which is *Bmal1*-dependent. Interestingly, the expression of some genes in the immune surveillance system also exhibits the circadian pattern (Fernandes et al, 1976; Wang et al, 2023). The number of circulating leukocytes in the blood reaches to the peak during the resting (daytime) period in mice (He et al, 2018), but it is unclear whether circulating leukocytes account for the difference in resistance to colitis between the resting and active periods in mice.

The circadian clock is involved in the pathogenesis of IBD. However, the application of chronopharmacology in the treatment of IBD is worth more attention. The small molecule SR9009 is an agonist of REV-ERBα/β that can reduce *Bmal1* expression (Zhuang et al, 2019). Previous studies have shown that SR9009 is effective in preventing and treating DSS-induced colitis and high-fat diet-induced obesity (Wang et al, 2018; Yu et al, 2021). Here, we explored the time-dependent effects of SR9009 on DSS-induced colitis. Mice treated with SR9009 at ZT0 showed the best therapeutic effect, with a stronger relief on intestinal inflammation and tissue protection, while those treated at ZT12 exhibited the worst efficacy. Our study gives an effectively time-dependent strategy for the treatment of IBD. In future work, the potential role of SR9009 should be further evaluated in clinical treatment of IBD.

In addition, we observed a downregulation of BMAL1 in clinically colonic samples from UC patients, which is consistent with its decreased expression in the late stages of DSS-treated mice. The decreased BMAL1 expression could be an adaption of the intestine to better regenerate the damaged tissue in the context of inflammation. The suppression of circadian clock was also observed in other tissue disorders. For instance, disruption of SCN function alleviated myocardial infarction induced cardiac dysfunction and cardiac fibrosis and the lethality of temperature imbalance in mice caused by time-restricted feeding treatment (Hao et al, 2023; Zhang et al, 2020). These observations suggest that mice may be able to rapidly respond to specific stress conditions by inhibiting the circadian clock, thereby better performing the repair process. Therefore, *Bmal1* depletion may exert a protective role in colitis by attenuating cell apoptosis. Targeting BMAL1 and inhibition of apoptosis may provide a potential strategy for prevention and treatment of colitis.

## Methods

**Table:** Reagents and tools table

Reagent/Resource	Reference or Source	Identifier or Catalog Number/RRID
**Experimental models**		
*Villin-CreERT2* mice (*M. musculus*)	(Ireland et al, 2004)	N/A
*Bmal1*^*fl/fl*^ mice (*M. musculus*)	(Hao et al, 2023)	N/A
**Antibodies**		
Rabbit anti-BMAL1	Cell Signaling Technology	14020S
Rabbit anti-cleaved-caspase3	Cell Signaling Technology	9664S
Rabbit anti-Bcl-2	Cell Signaling Technology	3498S
Mouse anti-caspase3	Santa Cruz	Sc-7272
Mouse anti-Bax	Santa Cruz	Sc-7480
Mouse anti-GAPDH	ZSGB-Bio	TA-08
Rabbit anti-active β-catenin	Cell Signaling Technology	8814
Mouse anti-Actin	ZSGB-Bio	TA-09
Rabbit anti-KI67	Abcam	Ab15580
Rabbit anti-OLFM4	Cell Signaling Technology	39141
Rabbit anti-lysozyme	Abcam	Ab108508
Rabbit anti-chromogranin A	Abcam	Ab15160
Mouse anti-E-cadherin	Cell Signaling Technology	14472
Mouse anti-IL1β	Santa Cruz	Sc-52012
Mouse anti-IFNγ	Santa Cruz	Sc-8423
Mouse anti-F4/80	Santa Cruz	Sc-377009
Rabbit anti-CD45	Abcam	Ab10558
Alexa Fluor 488-conjugated secondary antibody	Invitrogen	A28175
Alexa Fluor 594-conjugated secondary antibody	Invitrogen	A11037
Goat anti-rabbit IgG secondary antibody	Cell Signaling Technology	35401S
**Chemicals and other reagents**		
Tamoxifen	Sigma-Aldrich	T5648
DSS	MP Biomedicals	0216011090
SR9009	MCE	HY-16989
Cell strainer	Corning	352350
Matrigel	Corning	356231
Advanced DMEM/F12	Gibco	12634028
Penicillin/Streptomycin	HyClone	SV30010
GlutaMAX	Gibco	35050061
N2	Gibco	17502048
B27	Gibco	17504044
N-acetylcysteine	Selleck	S1623
EGF	Novoprotein	C029
Noggin	OrganRegen	807-NOG-1000
R-spondin1	OrganRegen	861-RS1-1000
CHIR	Selleck	S2924
Y-2763	Selleck	S1049
4-OH tamoxifen	Sigma	H7904
TrypLE	Gibco	12604021
TRIZOL	Thermo Fisher Scientific	15596026CN
HiScript III RT SuperMix	Vazyme	R323-01
ChamQ SYBR Color qPCR Master Mix	Vazyme	Q711-03
RIPA lysis buffer	Beyotime	P0013B
Cocktail	Roche	4693132001
H&E staining kit	Sangon Biotech	C0105
Alcain Blue staining kit	Sangon Biotech	C0153
DAPI	Solarbio	C0060
In-situ cell death detection kit	Roche	12156792910
PI	Selleck	S6874
GreenNuc™ Caspase-3 Assay Kit	Beyotime	C1168
CUT&Tag 4.0 High-Sensiticity Kit	Novoprotein	N259-YH01
**Software**		
Image J	https://imagej.net/ij/	N/A
GraphPad Prism10	https://www.graphpad-prism.cn/	N/A
IGV (2.6.2)	https://igv.org/	N/A
**Other**		
Real Time PCR system	Applied Biosystems	QuantStudio 3
microscope	Zeiss	LSM 980 META

### Human UC sample analysis

To investigate the expression of BMAL1 in the normal intestine and IBD, we collected paraffin tissue sections of UC samples and their adjacent normal colonic tissue samples (*n* = 9) from the 1st affiliated hospital, Jiangxi Medical College, Nanchang University. All samples were obtained with informed consent, and the study was approved by the First Affiliated Hospital of Nanchang University Medical Science Research Ethics Committee ((2024)CDYFYYLK(02-025)). The relevant clinical information was listed in Dataset EV4.

### Mice

*Villin-CreERT2* mice were a gift from Dr. Sylvie Robine. *Bmal1*^*fl/fl*^ mice from Dr. Ying Xu. *Villin-CreERT2; Bma1*^*fl/fl*^ mice were generated by crossing *Bmal1*^*fl/fl*^ mice with *Villin-CreERT2* mice. To activate Cre recombinase, mice were treated by intraperitoneal injection of tamoxifen (T5648, Sigma-Aldrich) in corn oil at a concentration of 20 mg/ml for 5 consecutive days. *Bmal1*^*fl/fl*^ mice were used as the control in all animal experiments. The following PCR primers (5′–3′) were used for genotyping: Forward primer, TGACCCTCATGGAAGGTTAGAA; Reverse prime, GGACATTGCATTGCATGTTGG.

Mice were housed in a pathogen-free facility under a 12 h light/12 h dark cycle at controlled room temperature of 22–25 °C with free access to water and food in Guangzhou National Laboratory. All mice used in study were generated on a C57BL/6 genetic background and aged 2–4 months from the same litter. All animal experimental procedures were conducted in accordance with the appropriate guidelines and approved by the ethical committee for the use of laboratory animals at Guangzhou National Laboratory (GZLAB-AUCP-2023-01-A01).

### DSS-induced colitis model

The experiments were conducted as described previously (Wei et al, 2023a). In this study, two main methods were used to induce the acute experimental colitis. One was through continuous gavaging for consecutive 5 days by giving a dose of 4 g/kg/day DSS (MP Biomedicals) solutions to 8–10-week-old male mice, followed by normal water until sacrificed at the designated time points. The other was by giving 3% DSS dissolved in their drinking water for 5 consecutive days and colon tissues were collected at the designated time points. To evaluate the optimal therapeutic effect of SR9009 on colitis, SR9009 (MCE, HY-16989, 50 mg/kg) was administered intraperitoneally once daily for 5 consecutive days at ZT0, ZT8, and ZT12, respectively, starting on the third day of DSS-induced colitis in mice. Colon tissues were subsequently collected at day 8. Control group intraperitoneal injection of vehicle (corn oil solution containing 5% DMSO). The severity of colitis was assessed by daily monitoring of the mice’s relevant physiological status, including body weight, posture, and stool. DAI scores was determined based on the following criteria: weight loss (0 for no change, 1 for 5–10%, 2 for 10–15%, and 3 for >15%), body posture (0 for smooth fur without a hunchback, 1 for mild fur and hunchback, 2 for moderate fur and hunchback, and 3 for severe fur and heavy hunchback), and stool consistency (0 for normal, 1 for mild loose stool, 2 for loose stool and diarrhea, and 3 for bloody stools).

After euthanizing the mice, the intestines were collected and washed with iced PBS and fixed in 4% paraformaldehyde at 4 °C overnight. After dehydration and processing, the intestinal tissue was embedded in paraffin wax. Freshly intestines embedded in paraffin were used to prepare 5-μm-thick sections. These paraffin tissue sections were used to analyze its immune cell ratio and histological score and so on. Histological damage was scored based on goblet cells loss, mucosa thickening, inflammatory cell infiltration, submucosa cell infiltration, ulcers, and crypt abscesses. A score of 1–3 or 1–4 was given for each parameter with a maximal total score of 20, as previously described (Wang et al, 2018).

### Intestinal crypt isolation and organoid culture

Intestinal crypts were isolated and cultured as previously described (Qi et al, 2017). Briefly, mouse large intestines were isolated and longitudinally washed several times with cold PBS. The large intestine was incubated in cold PBS containing 2 mM EDTA at 4 °C for 30 min. Afterwards, the inner lumen of the large intestine is scraped with a scalpel to remove the crypts. The crypts were then resuspended in cold PBS and filtered through a 70 μm cell strainer (Corning) for purification. The fraction enriched through centrifugation at a speed of 500 × *g* for 1 min and embedded in Matrigel (Corning) and seeded in 24-well plates. The crypts embedded in Matrigel were cultured in ENRCY medium (Advanced DMEM/F12 supplemented with Penicillin/Streptomycin, GlutaMAX, N2, B27 and N-acetylcysteine) containing EGF (50 ng/ml, Novoprotein), Noggin (100 ng/ml, R&D), R-spondin1 (500 ng/ml, R&D), CHIR (5 µM, Selleck) and Y-27632 (10 µM, Selleck). To get rid of non-epithelial cells, subsequent experiments were started after 2–3 generations of organoid passaging. To activate Cre recombinase in organoids, 4-OHT (200 nM, Sigma) was added to the culture medium for 24 h, organoids were passaged at day 3 and observed or harvested for subsequent analysis at day 6. To assess the organoid growth, organoids were digested with TrypLE (Gibco, 12604021) into single cell culture for organoids diameter statistic and immunoblotting. The diameter was measured by ImageJ.

### RNA isolation and qPCR

Total RNA was extracted and purified from colonic crypts using TRIZOL reagent (Thermo Fisher Scientific) according to the manufacturer’s instructions. The same amount (1 µg) of total RNA were reverse-transcribed to cDNA using the HiScript III RT SuperMix (Vazyme). Quantitative real-time PCR (qRT-PCR) was performed on a QuantStudio 3 Real Time PCR system (Applied Biosystems) using the ChamQ SYBR Color qPCR Master Mix (Vazyme). The mRNA expression levels were normalized to a housekeeping gene, and the quantitative PCR primers were listed in Dataset EV5.

### Immunoblotting

The experiments were conducted as described previously (Gao et al, 2010). The colonic crypts were collected and lysed in RIPA lysis buffer (Beyotime) added with a protease and phosphatase inhibitor cocktail (Roche). Primary antibody used included rabbit anti-BMAL1 (CST, 14020S, 1:1000), rabbit anti-c-caspase 3 (CST, 9664S, 1:1000), rabbit anti-Bcl-2 (CST, 3498S, 1:1000), mouse anti-caspase 3 (Santa Cruz, sc-7272, 1:500), mouse anti-Bax (Santa Cruz, sc-7480, 1:500), mouse anti-GAPDH (ZSGB-Bio, TA-08, 1:10,000), rabbit anti-active β-catenin (CST, 8814, 1:1000) and mouse anti-Actin (ZSGB-Bio, TA-09, 1:10,000).

### Hematoxylin and eosin (H&E), Alcian Blue, immunofluorescence (IF) and immunohistochemistry (IHC)

The experiments were conducted as described previously (Wei et al, 2023b). Paraffin tissue sections obtained as described above were subjected to H&E or other staining. H&E staining and Alcian Blue staining were performed using a kit (Sangon Biotech) according to the manufacture’ instructions. For immunohistochemistry staining, human and mouse paraffin tissue sections were deparaffinized in xylene and graded to reduce the alcohol concentration, followed by antigenic repair with citrate and blocked for 60 min in PBS solution containing 0.1% Triton X-100 containing 3% BSA. Sections were then incubated with primary antibodies at 4 °C overnight, followed by adding secondary horseradish peroxidase conjugated antibodies (Invitrogen, 1:200) as indicated for 1 h and diaminobenzidine (DAB) chromogen. Finally, slides were dehydrated and mounted with neutral balsam. For immunofluorescence staining, sections were treated with the same antigenic repair treatment and blocked for 60 min in PBS solution containing 0.1% Triton X-100 containing 3% BSA. Next, the sections were incubated overnight at 4 °C with primary antibodies. Primary antibodies used included rabbit anti-BMAL1 (CST, 14020S, 1:400), rabbit anti-KI67 (Abcam, ab15580, 1:1000), rabbit anti-OLFM4 (CST, 39141, 1:500), rabbit anti-lysozyme (Abcam, ab108508, 1:500), rabbit anti-chromogranin A (Abcam, ab15160, 1:500), mouse anti-E-cadherin (CST, 14472, 1:1000), mouse anti-IL1β (Santa Cruz, sc-52012, 1:200), mouse anti-IFNγ (Santa Cruz, sc-8423, 1:100) mouse anti-F4/80 (Santa Cruz, sc-377009, 1:400), rabbit anti-CD45 (Abcam, ab10558, 1:1000) and rabbit anti-c-caspase 3 (CST, 9664S, 1:1000). After incubation with the primary antibodies, the sections were incubated with Alexa Fluor 488-, Alexa Fluor 594-conjugated secondary antibodies (Invitrogen, 1:1000) for 2 h at room temperature. Nuclei were stained with DAPI (Solarbio), and slides were dried and mounted using Antifade Mounting Medium (Invitrogen). Images were captured using a Zeiss LSM 980 META microscope, and images were processed using photoshop software. All images shown in this experiment are representative of at least three randomly selected attempts.

### TUNEL staining

Paraffin tissue sections were subjected to TUNEL staining using the in situ cell death detection kit (Roche) according to the manufacturer’s instructions, followed by DAPI co-staining as described above. Images were acquired using a Zeiss LSM 980 microscope.

### PI staining of organoids

Intestinal organoids in Matrigel stained with propidium iodide (PI) at a final concentration of 20 µg/ml each. The organoids were stained for 10 min and then washed three times with cold PBS for subsequent analysis microscopy. PI intensity was measured by ImageJ.

### C-caspase 3 staining of organoids

The GreenNuc™ Caspase-3 Assay Kit for colonic organoids purchased from Beyotime (Nanjing, China) was used to measure caspase 3 activity in cells according to the manufacturer’s introductions. Intestinal organoids in Matrigel stained with caspase 3 substrate at a final concentration of 5 µM. The organoids were stained for 30 min and then washed three times with cold PBS for subsequent analysis microscopy. C-caspase 3 intensity was measured by ImageJ.

### RNA-seq library construction and analysis

The RNA purity of RNA-seq samples was detected using Nanophotometer spectrophotometer (IMPLEN, CA, USA), and then the concentration and integrity of RNA samples were tested using Agilent Technologies 2100 RNA Nano 6000 Assay Kit (Agilent Technologies, CA, USA). The mRNA library was prepared using the NEBNext® UltraTM RNA Library Prep Kit for Illumina and paired-end sequencing was conducted on the Illumina platform employing a PE150 strategy by Novogene Bioinformatics Technology Co., Ltd (Beijing, China).

RNA-seq reads were aligned to the reference genome GRCm38 using the STAR software (version 2.2.1). Then the differential expression analysis was performed employing DESeq2 software (version 1.40.1). For functional enrichment analyses, GO and KEGG enrichment analyses were conducted, utilizing ClusterProfiler software (version 4.9.0). Gene Set Enrichment Analysis (GSEA) was performed using the fgsea software (version 1.27.0), Gene Set Variation Analysis (GSVA) was conducted with the R/Bioconductor GSVA package (version 4.3.2) to obtain pathway enrichment scores, and visualization was conducted using the ggplot2 package (version 3.4.2).

### CUT&Tag-seq

For CUT&Tag-seq, the libraries were prepared using a CUT&Tag 4.0 High-Sensitivity Kit (Novoprotein; N259-YH01) according to the manufacturer’s instructions. Colonic organoids were fixed in 1% formaldehyde and lysed with lysis buffer, followed by digestion with micrococcal nuclease. We used a rabbit anti-BMAL1 antibody (CST; 14020S) and goat anti-rabbit IgG secondary antibody.

CUT&Tag-seq reads were aligned to the reference genome GRCm38 using the hisat2 software (version 2.2.1), and only uniquely and non-duplicate mapped reads were used to perform the downstream analysis. The reads coverage and depth were calculated by samtools software (version 1.9). The BAM files were subjected to peak calling analysis using MACS2 (version 2.2.7.1) to identify regions in the genome exhibiting significant enrichment of reads. The identified peaks were further subjected to gene annotation analysis and visualization using the Chipseeker package (version 1.37.0) and IGV software (version 2.16.1).

### Circadian analyses

The experiments were conducted as described previously (De Los Santos et al, 2020; Wu et al, 2016). The rhythmicity of the oscillatory expression is measured by the JTK cycle through the MetaCycle R package. With the settings of Period = 24 h and adj.*p* < 0.05, oscillatory patterns were defined as rhythmic.

### Statistical analysis

All experiments were conducted independently at least three times. Each experiment contained at least two biological replicates. The data were presented in column graphs as means ± SD. The normality of all samples was tested using the Shapiro–Wilk test. Unpaired two-tailed t-test, one-way ANOVA, and two-way ANOVA analysis were used to compare differences as indicated in the figure legends. The statistical analysis was performed with GraphPad Prism 9.0 software. Ns indicates no significance. Exact details are listed in Dataset EV6, and the exact *P* values are displayed in the figures.

## Supplementary information

Peer Review File

Dataset EV1

Dataset EV2

Dataset EV3

Dataset EV4

Dataset EV5

Dataset EV6

Figure EV2F Source Data

Expanded View Figures

## Data Availability

The bulk-RNA sequencing data and CUT&Tag sequencing data have been deposited in the Gene Expression Omnibus (GEO) under the accession code GSE271317 and GSE254225. The source data of this paper are collected in the following dataset: S-BIAD1672. The source data of this paper are collected in the following database record: biostudies:S-SCDT-10_1038-S44319-025-00464-y.
