# Supplementary figures and images for: Genetic disruption of the circadian gene Bmal1 in the intestinal epithelium reduces colonic inflammation
Source: EMBO Rep. 2025 Apr 30;26(12):3138–61. doi: 10.1038/s44319-025-00464-y (PMC12187941; doi:10.1038/s44319-025-00464-y)

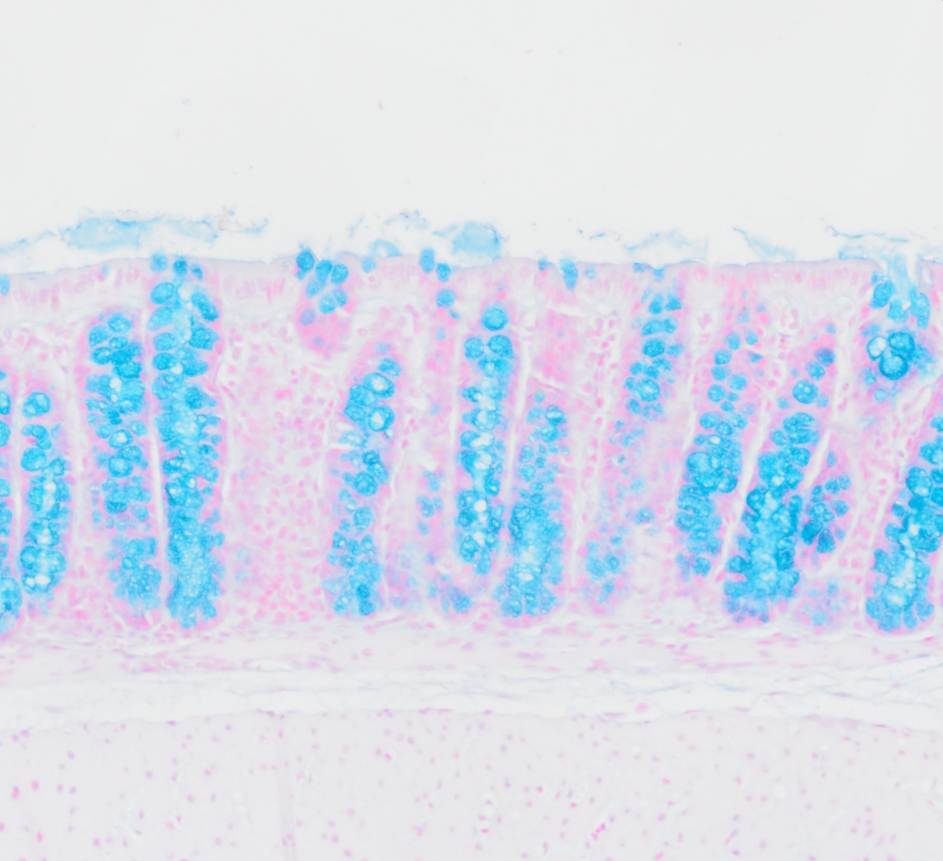

Supplement: Supplementary file 8 — Figure EV2F Source Data [file 44319_2025_464_MOESM8_ESM.zip › Bmal1 cKO large intestine(Enlarge image).tif]

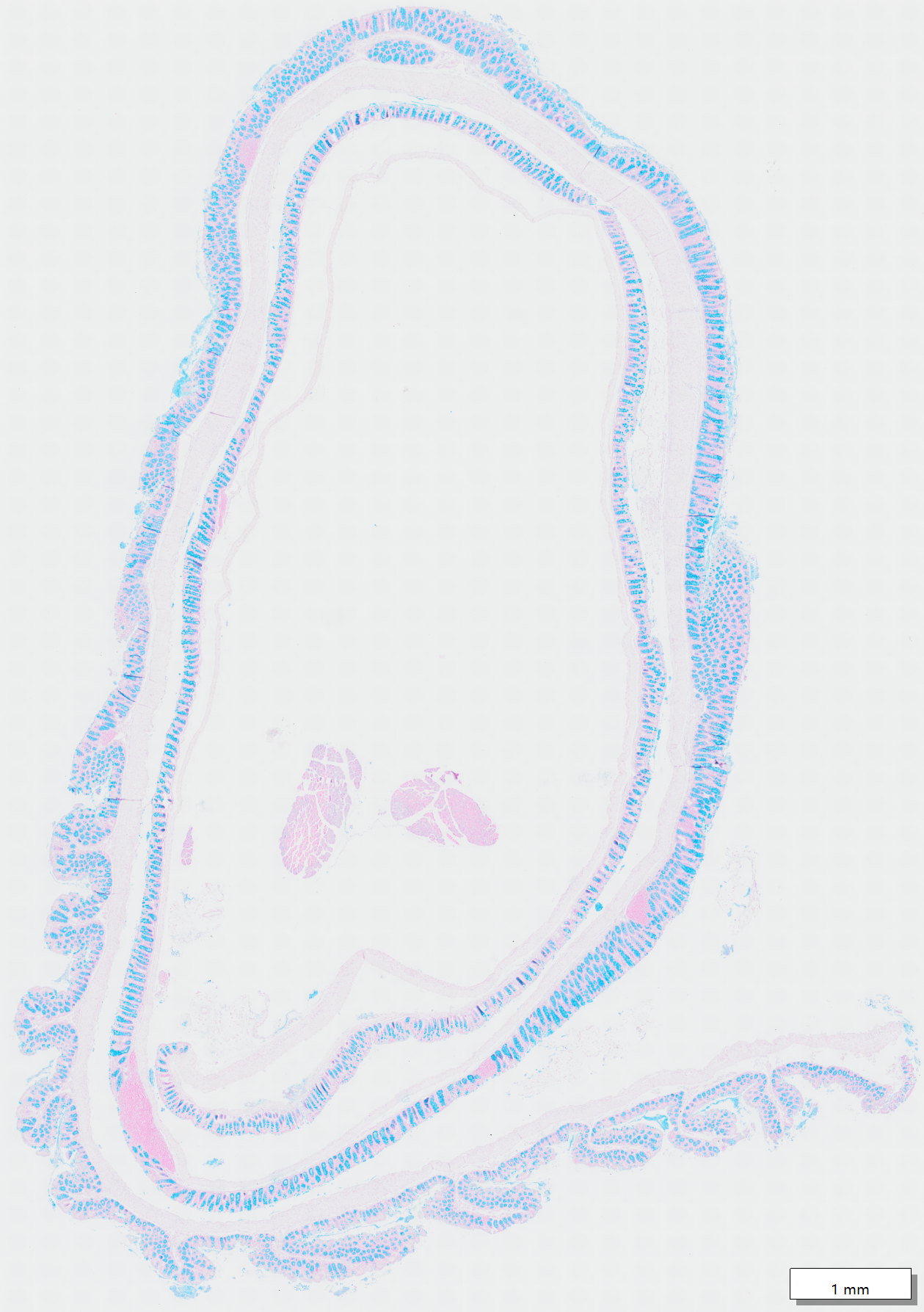

Supplement: Supplementary file 8 — Figure EV2F Source Data [file 44319_2025_464_MOESM8_ESM.zip › Bmal1 cKO large intestine.tif]

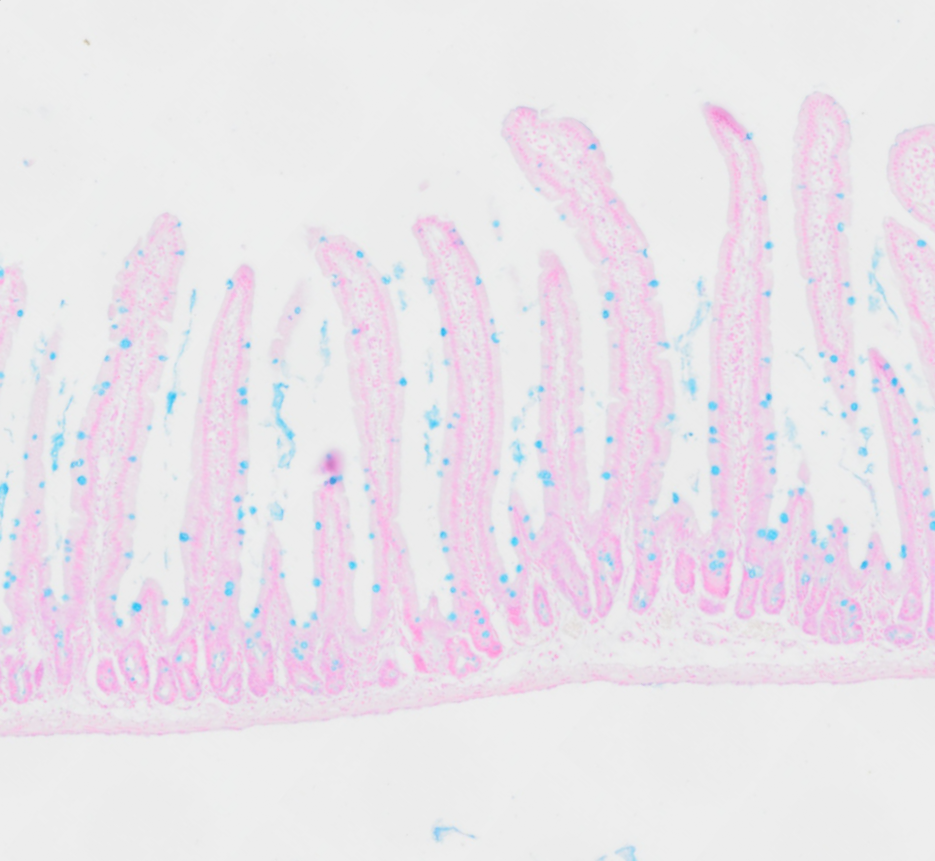

Supplement: Supplementary file 8 — Figure EV2F Source Data [file 44319_2025_464_MOESM8_ESM.zip › Bmal1 cKO small intestine (Enlarge image).tif]

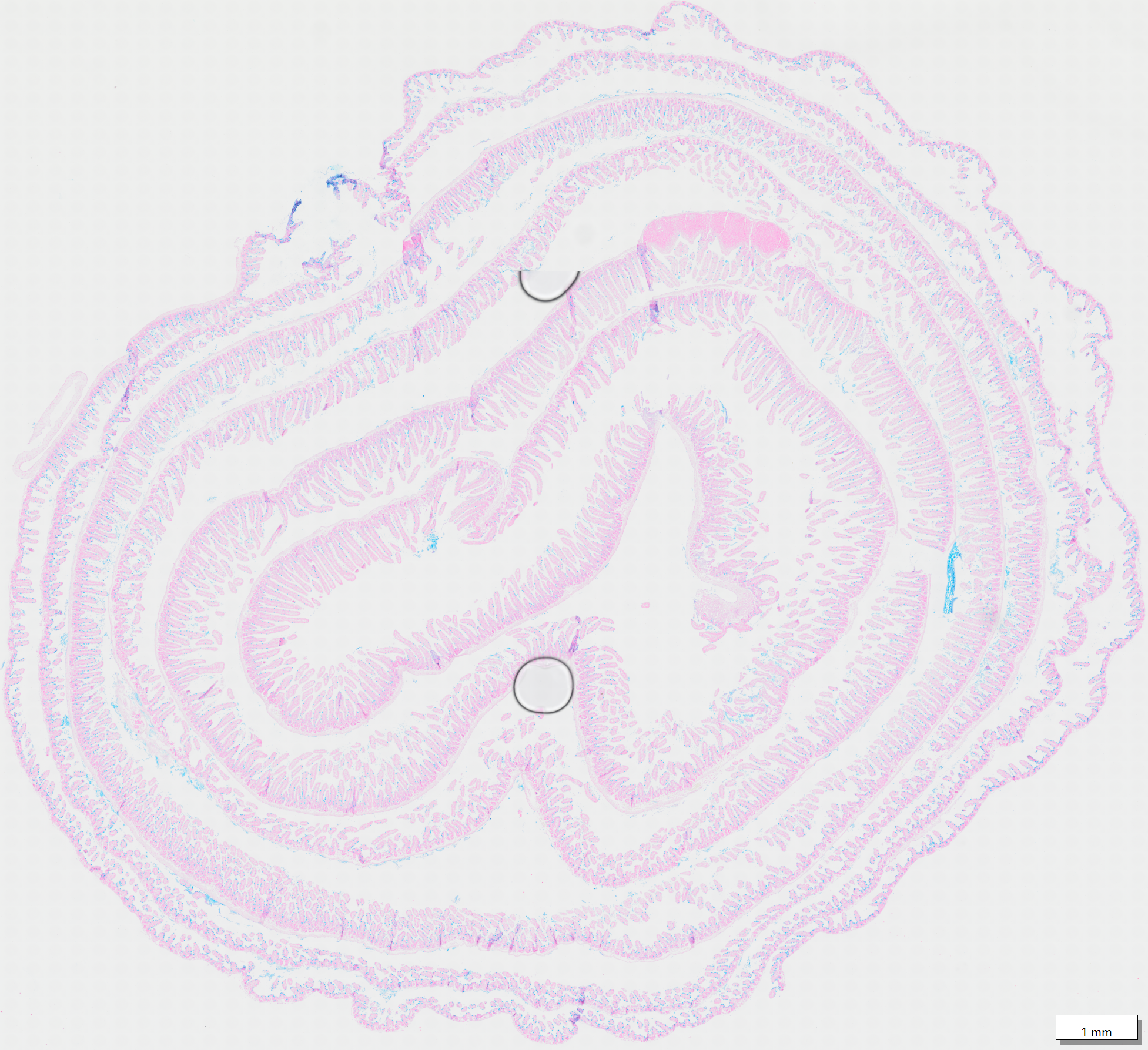

Supplement: Supplementary file 8 — Figure EV2F Source Data [file 44319_2025_464_MOESM8_ESM.zip › Bmal1 cKO small intestine.tif]

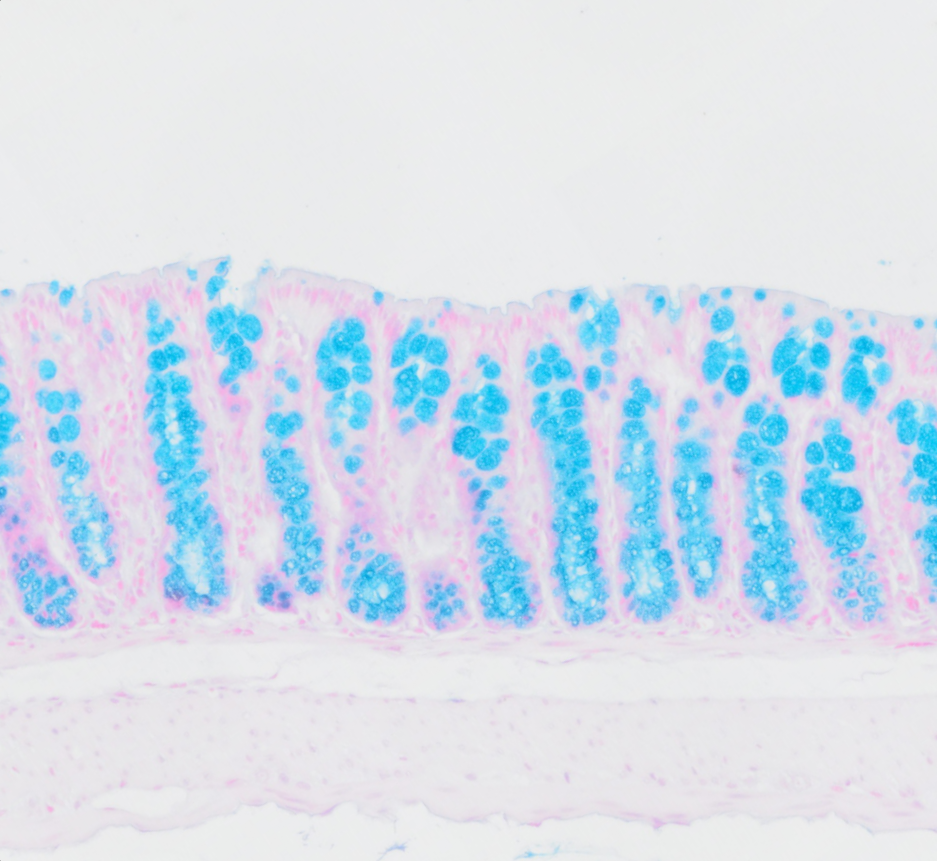

Supplement: Supplementary file 8 — Figure EV2F Source Data [file 44319_2025_464_MOESM8_ESM.zip › Control large intestine (Enlarge image).tif]

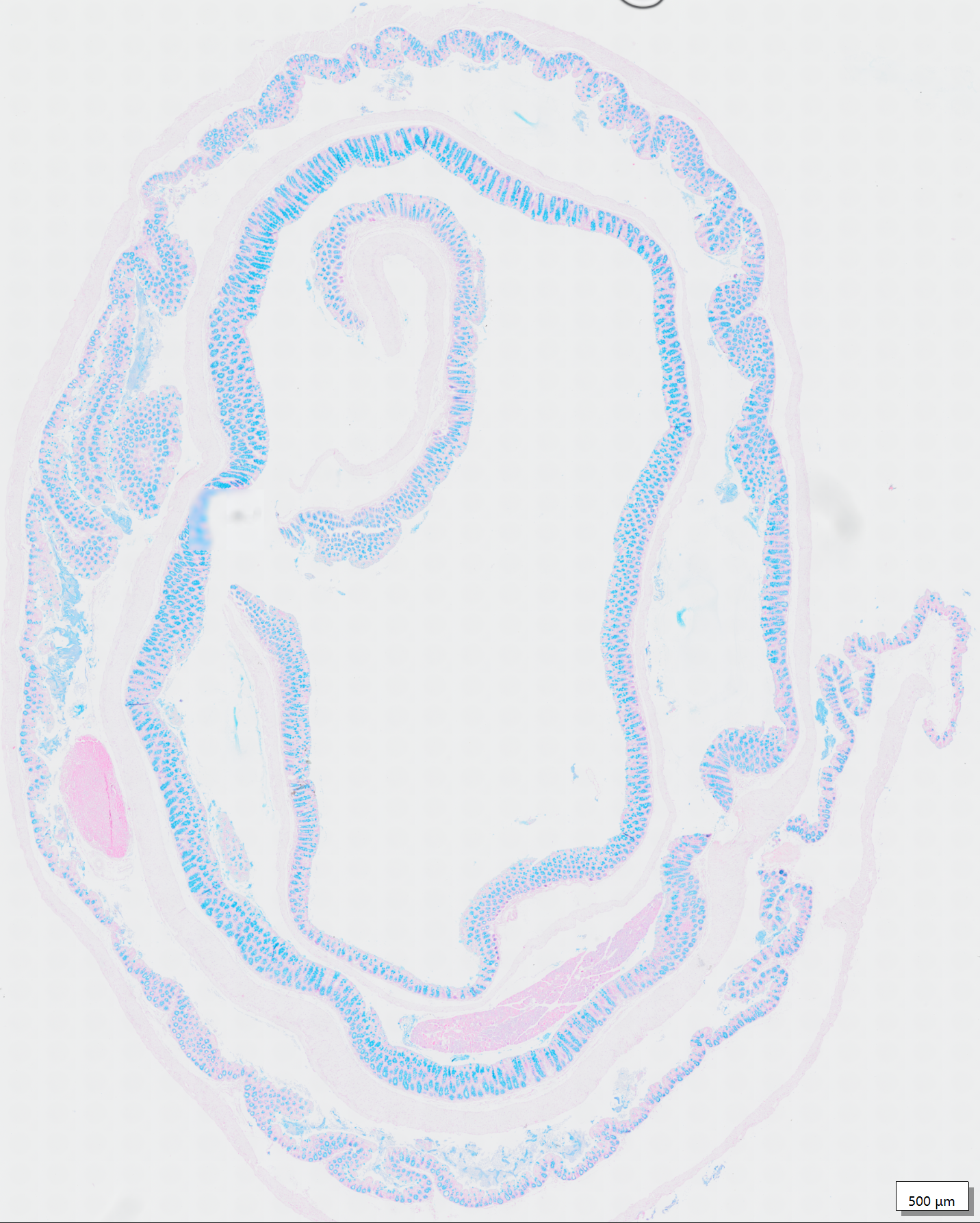

Supplement: Supplementary file 8 — Figure EV2F Source Data [file 44319_2025_464_MOESM8_ESM.zip › Control large intestine.tif]

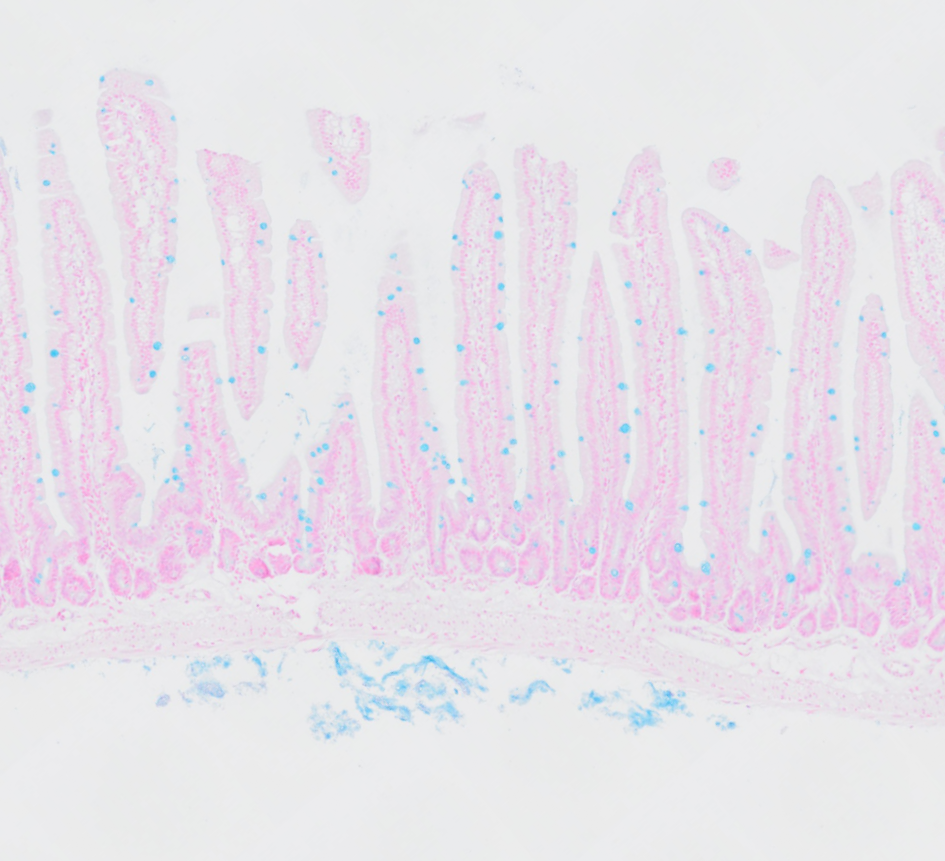

Supplement: Supplementary file 8 — Figure EV2F Source Data [file 44319_2025_464_MOESM8_ESM.zip › Control small intestine (Enlarge image).tif]

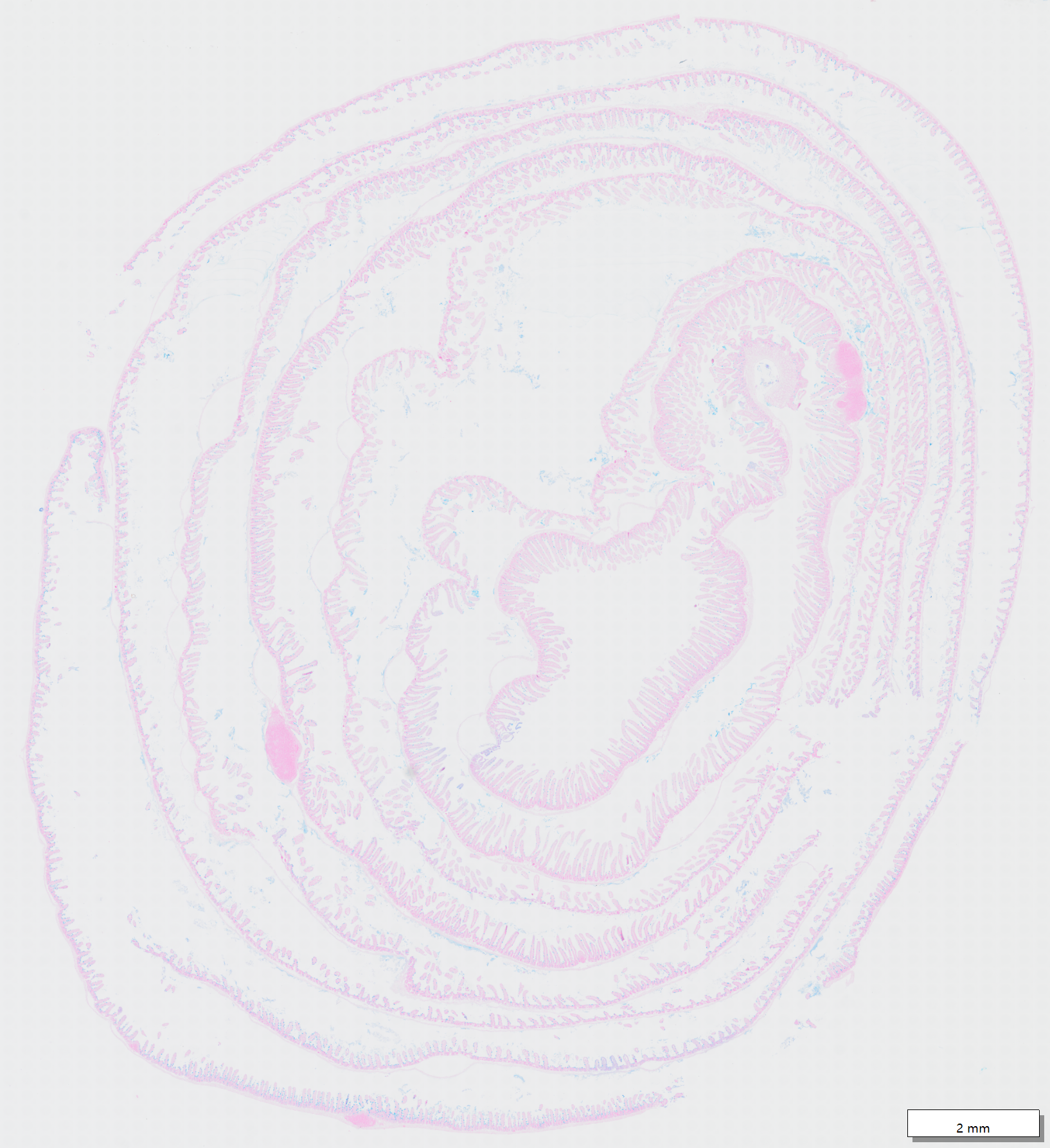

Supplement: Supplementary file 8 — Figure EV2F Source Data [file 44319_2025_464_MOESM8_ESM.zip › Control small intestine.tif]

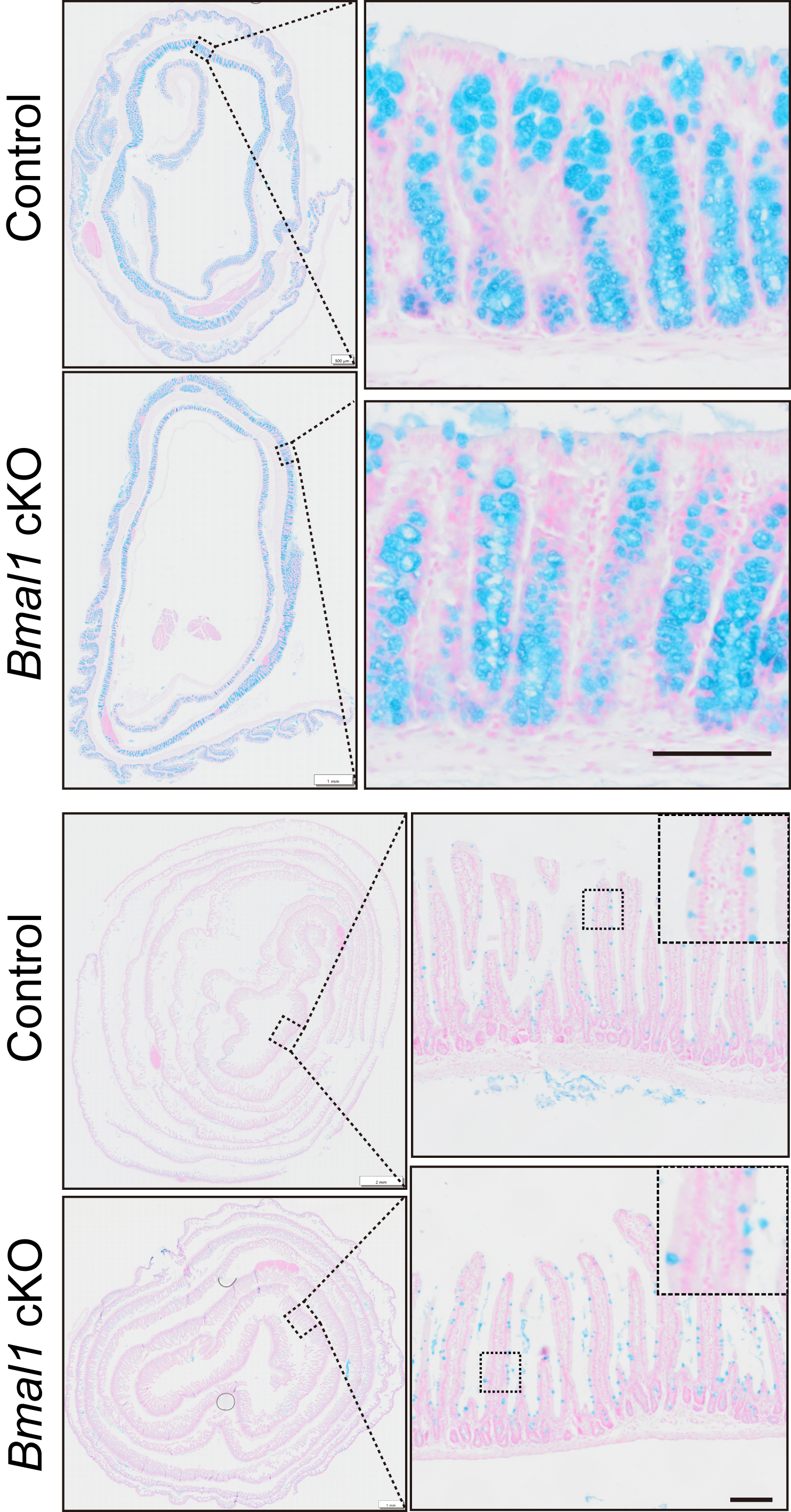

Supplement: Supplementary file 8 — Figure EV2F Source Data [file 44319_2025_464_MOESM8_ESM.zip › Source data of Fig. EV2F.tif]
