# Supplementary material for: Genetic disruption of the circadian gene Bmal1 in the intestinal epithelium reduces colonic inflammation
Source: EMBO Rep. 2025 Apr 30;26(12):3138–61. doi: 10.1038/s44319-025-00464-y (PMC12187941; doi:10.1038/s44319-025-00464-y)
Supplement: Supplementary file 9 — Expanded View Figures [file 44319_2025_464_MOESM9_ESM.pdf]

Expanded View Figures

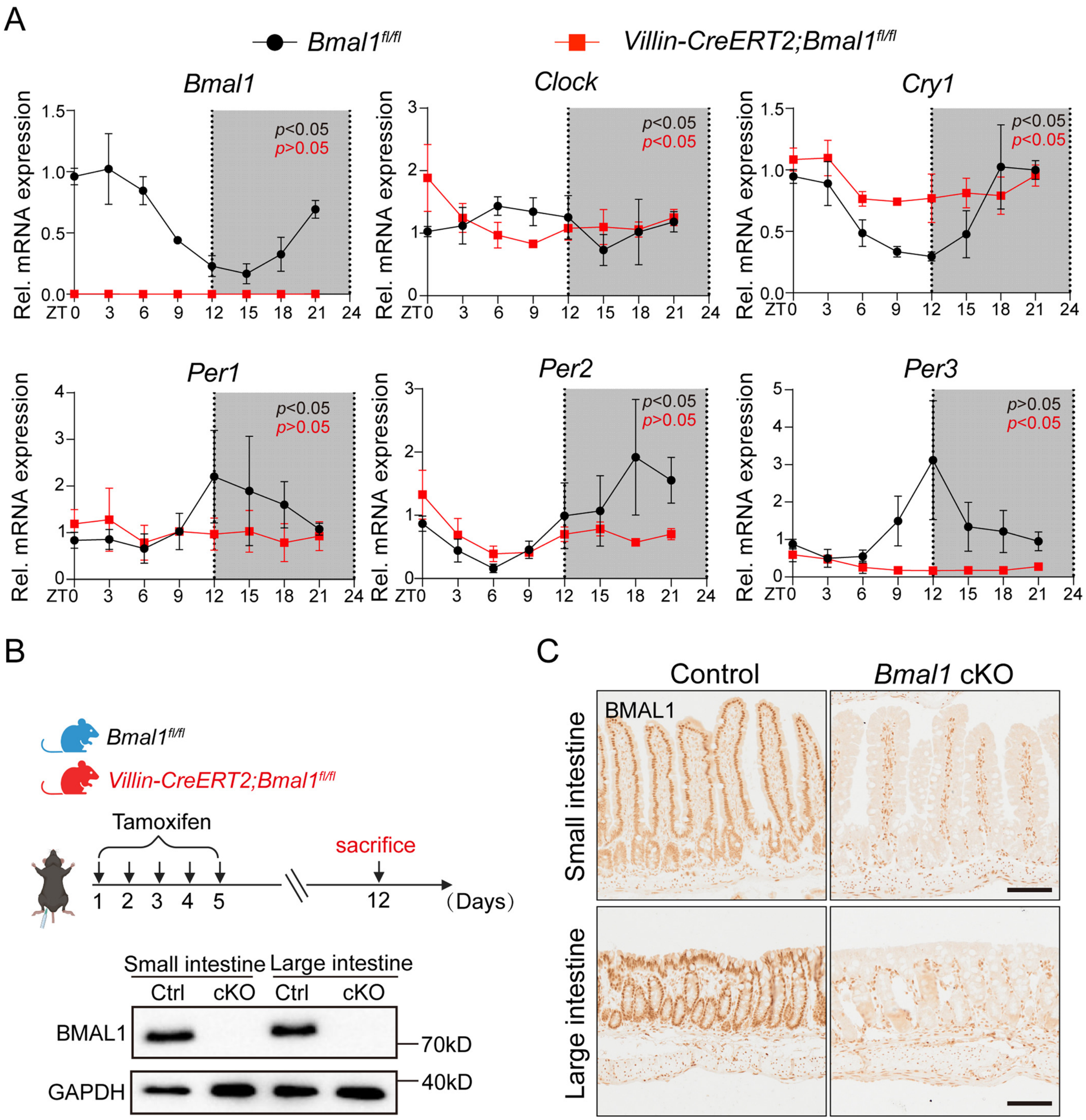

Figure EV1. Dynamic expression of circadian clock genes in the colonic epithelium.

(**A**) RT-qPCR analysis of mRNA levels of circadian genes in the colonic crypts of control and *Bmal1* cKO mice. The colonic epithelia were collected at 3-h intervals over a 24-h period in a day. n = 3 mice for each group. Shaded areas represent mice in the dark. (**B**) After consecutive daily tamoxifen injection for 5 days to induce specific knockout of *Bma1* in the mouse intestinal crypts, tissues were harvested 7 days later for immunoblotting to examine BMAL1 and GAPDH protein levels. (**C**) Immunohistochemical analysis of BMAL1 in intestine sections from control and *Bmal1* cKO mice. Scale bar: 100 µm. Data information: Data are presented as mean ± SD. The rhythmicity of the oscillating pattern was measured by the JTK cycle through the MetaCycle R package. With the settings of Period = 24 h and adj.$p < 0.05$, expression patterns were then defined as rhythmic.

 © The Author(s)

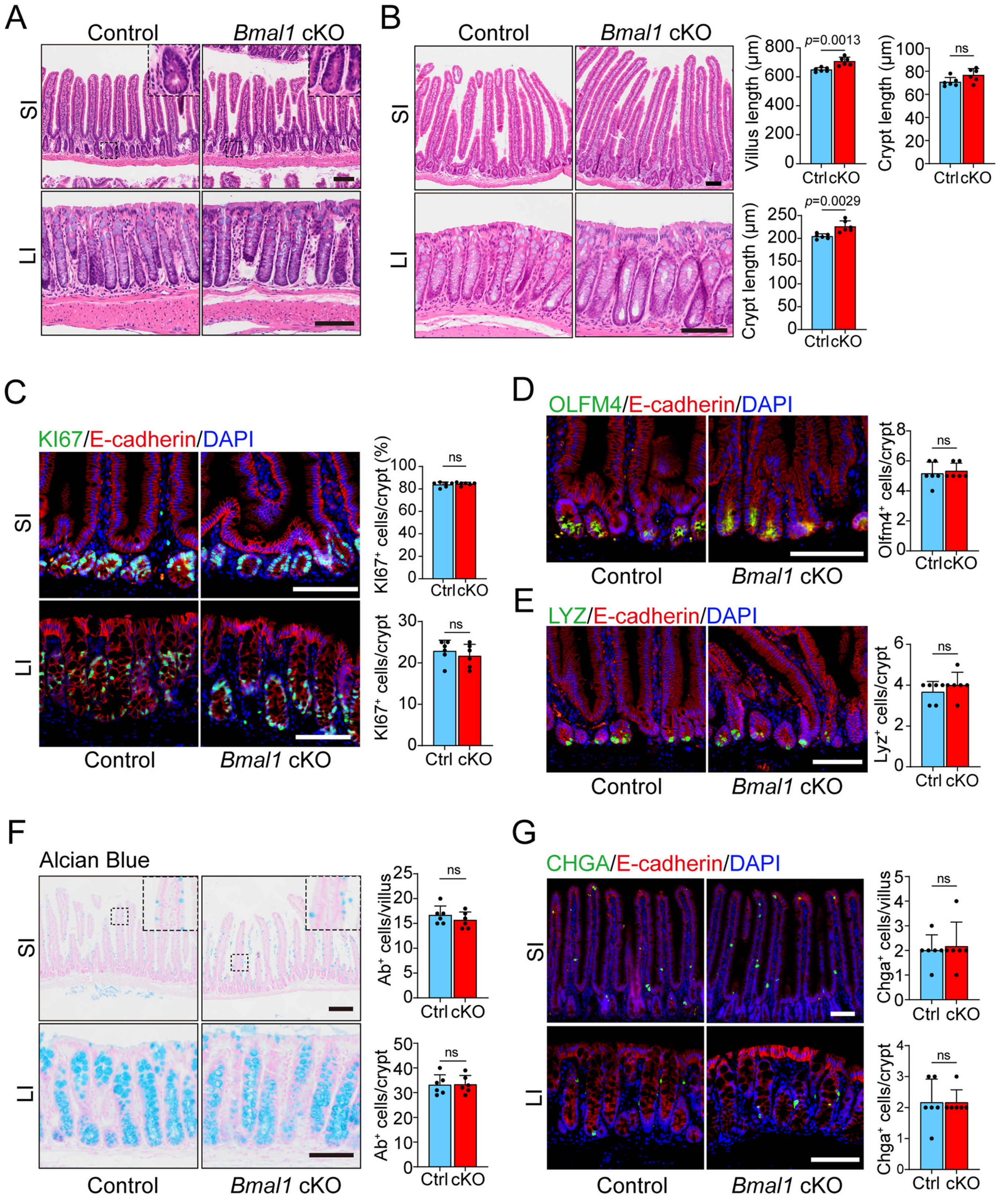

A
Control
Bmal1 cKO
SI
LI
B
Control
Bmal1 cKO
SI
LI
Villus length (μm)
p=0.0013
Crypt length (μm)
ns
p=0.0029
Ctrl cKO
C
KI67/E-cadherin/DAPI
SI
LI
Control
Bmal1 cKO
KI67+ cells/crypt (%)
KI67+ cells/crypt
ns
D
OLFM4/E-cadherin/DAPI
Olfm4+ cells/crypt
E
LYZ/E-cadherin/DAPI
Lyz+ cells/crypt
F
Alcian Blue
Ab+ cells/villus
Ab+ cells/crypt
G
CHGA/E-cadherin/DAPI
Chga+ cells/villus
Chga+ cells/crypt

Figure EV2. Ablation of *Bmal1* in the colonic epithelium does not affect the renewal and differentiation of colonic epithelial cells.

(**A**) Histological images of the small intestine (SI) and large intestine (LI) from control and *Bmal1* cKO mice. Scale bar: 100 µm. (**B**) Histological images and quantification of villus length (top, left) and crypt length of the proximal small intestine (top, right) and distal large intestine (bottom) from control and *Bmal1* cKO mice. n = 6 mice for each group. Scale bar: 100 µm. (**C**) Immunofluorescence staining (left) of $KI67^+$ TA cells in the SI and LI from control and *Bmal1* cKO mice. Quantification (right) of $KI67^+$ TA cells in the SI (top) and LI (bottom) from control and *Bmal1* cKO mice. Epithelial cells were stained by E-cadherin. n = 6 mice for each group. Scale bar: 100 µm. (**D**, **E**) IF staining for $OLFM4^+$ ISCs and LYZ^+ Paneth cells (left) and quantification results of $OLFM4^+$ and LYZ^+ cells (right) in the small intestinal from control and *Bmal1* cKO mice. n = 6 mice for each group. Scale bar: 100 µm. (**F**) Alcain blue staining of goblet cells in the SI and LI from control and *Bmal1* cKO mice. Quantification (right) of goblet cells in the SI (top) and LI (bottom) from control and *Bmal1* cKO mice. n = 6 mice for each group. Scale bar: 100 µm. (**G**) IF staining (left) of $CHGA^+$ enteroendocrine cells in the SI and LI from control and *Bmal1* cKO mice. Quantification (right) of $CHGA^+$ cells in the SI (top) and LI (bottom) from control and *Bmal1* cKO mice. Epithelial cells were stained by E-cadherin. n = 6 mice for each group. Scale bar: 100 µm. Data information: Data are presented as mean ± SD. The data were analyzed by two-tailed Student's t-test (**B**–**G**). The exact *P* values are displayed. ns, no significance. Source data are available online for this figure.

 © The Author(s)

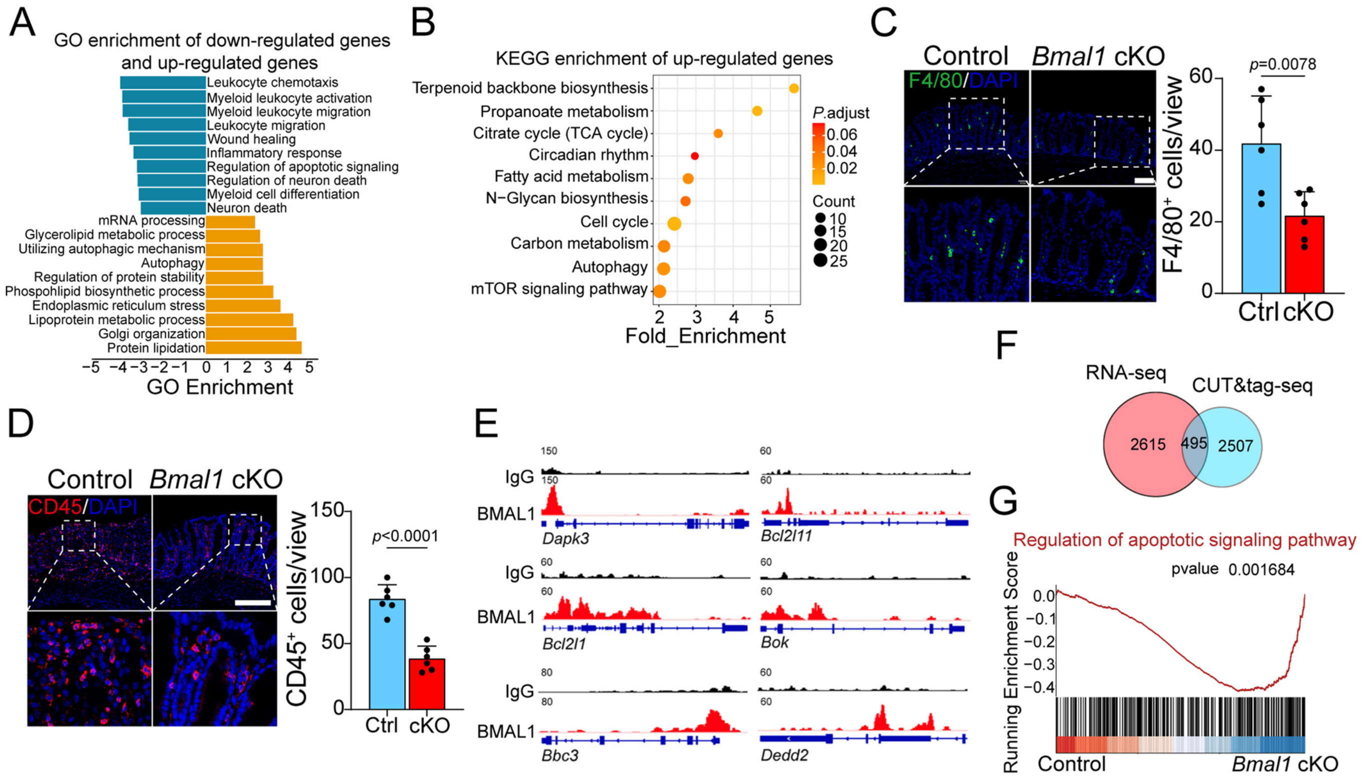

Figure EV3. Ablation of *Bmal1* in the intestinal epithelium is accompanied by reduced inflammation and immune response.

(**A**, **B**) GO enrichment (**A**) of upregulated genes and downregulated genes in the colonic crypts of *Bmal1* cKO mice and KEGG enrichment (**B**) of upregulated genes in the colonic crypts. The tissues were collected from control and *Bmal1* cKO mice, respectively, after 3% DSS drinking for 5 days, followed by a switch to normal water drinking for 2 days. (**C**, **D**) IF staining for F4/80 (**C**) and CD45 (**D**) and quantification of F4/80$^+$ and CD45$^+$ cells (right) in the distal colon sections from control and *Bmal1* cKO mice. n = 6 mice for each group. Scale bar: 100 μm. (**E**) Genomic views of BMAL1 CUT&Tag-seq assay enrichment at the promoters of the apoptosis-related genes in colonic organoids from control mice. (**F**) Venn diagram shows the overlapped genes between anti-BMAL1 CUT&Tag-seq assay and downregulated genes by RNA-seq in *Bmal1* cKO mice. The colonic organoids from wild-type mice were collected for CUT&Tag-seq assay. The tissues were collected from *Bmal1* cKO mice after 3% DSS drinking for 5 days, followed by a switch to normal water drinking for 2 days for RNA-seq. (**G**) GSEA of apoptosis gene sets enriched with decreased genes in colonic crypts from *Bmal1* cKO mice. Data information: Data are presented as mean ± SD. The data were analyzed by two-tailed Student's t-test (**C**, **D**). The exact *P* values are displayed.

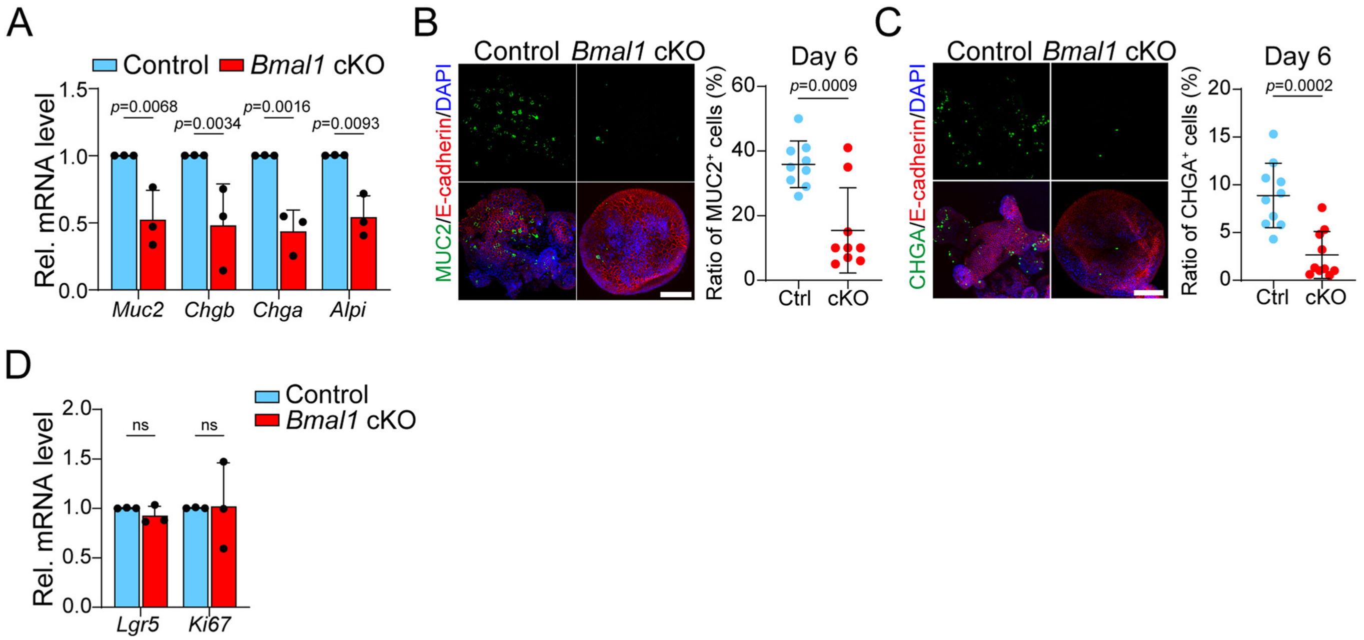

Figure EV4. Ablation of *Bmal1* suppresses differentiation but does not affect stemness and proliferation in vitro.

(**A**) RT-qPCR analysis of the mRNA expression of *Muc2*, *Chgb, Chga* and *Alpi* from control or *Bmal1* cKO organoids on day 6. n = 3 biological replicates. (**B**, **C**) Immunofluorescence staining for MUC2 (**B**) and CHGA (**C**) and quantification of $MUC2^+$ and $CHGA^+$ cells (right) from control or *Bmal1* cKO organoids. n = 9/10 organoids for each group. Scale bar: 100 μm. (**D**) RT-qPCR analysis for the mRNA expression of *Lgr5* and *Ki67* in control and *Bmal1* cKO organoids at day 3 starting at single cells. n = 3 biological replicates. Data information: Data are presented as mean ± SD with statistical analyses determined by two-tailed Student's t-test (**B**, **C**) and two-way ANOVA with Tukey's multiple comparisons test (**A**, **D**). The exact *P* values are displayed. ns, no significance.

 © The Author(s)

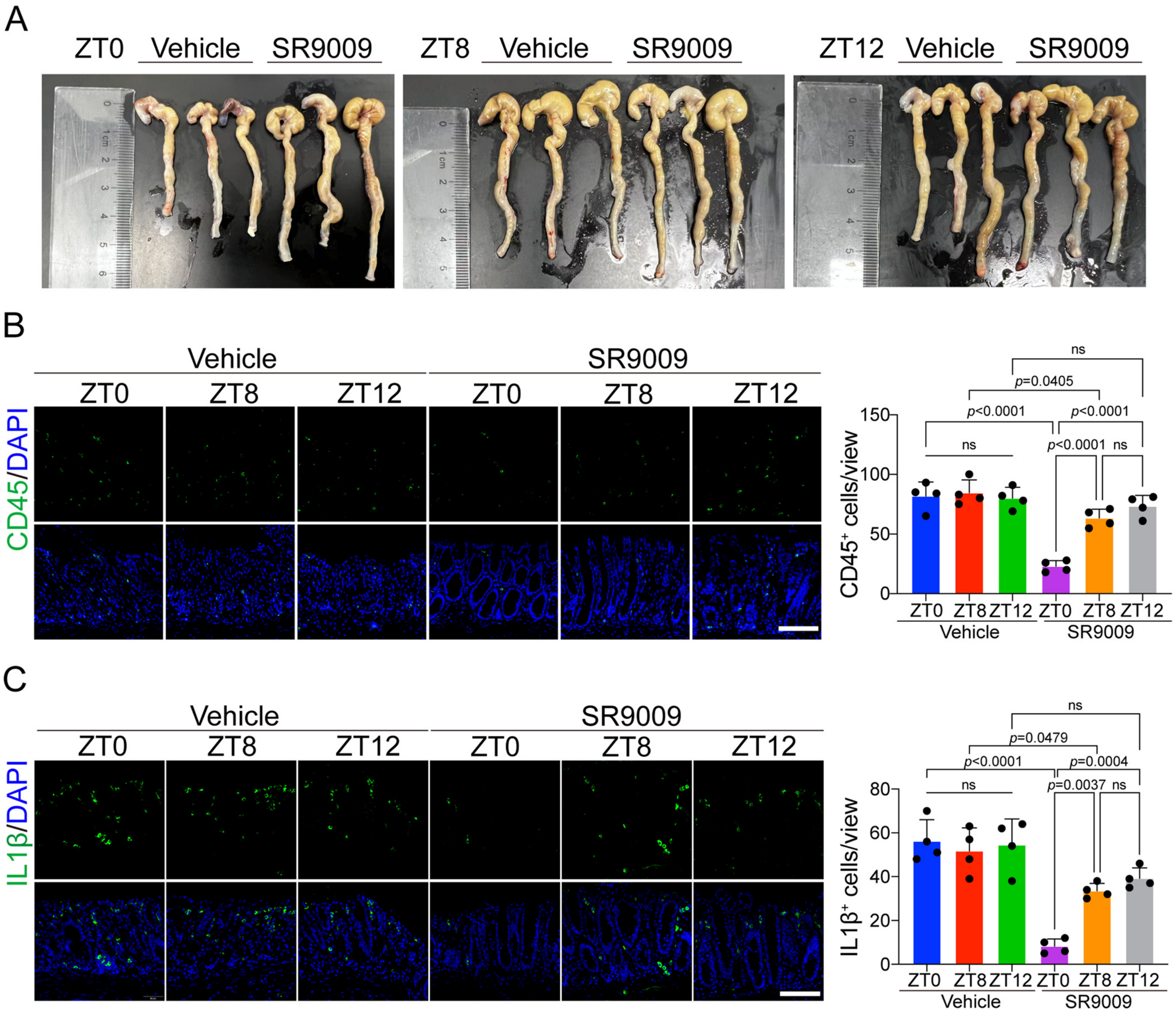

Figure EV5. Mice treated with SR9009 at ZT0 have less immune cell infiltration.

(**A**) Colon length from six groups of mice. (**B**, **C**) Immunofluorescence staining for CD45 (**B**) and IL1β (**C**) and quantification of $CD45^+$ and $IL1β^+$ cells (right) in the distal colon sections from six group of mice. n = 4 mice for each group. Scale bar: 100 µm. Data information: Data are presented as mean ± SD, analyzed by one-way ANOVA with Tukey's multiple comparisons test (**B**, **C**). The exact *P* values are displayed. ns, no significance.

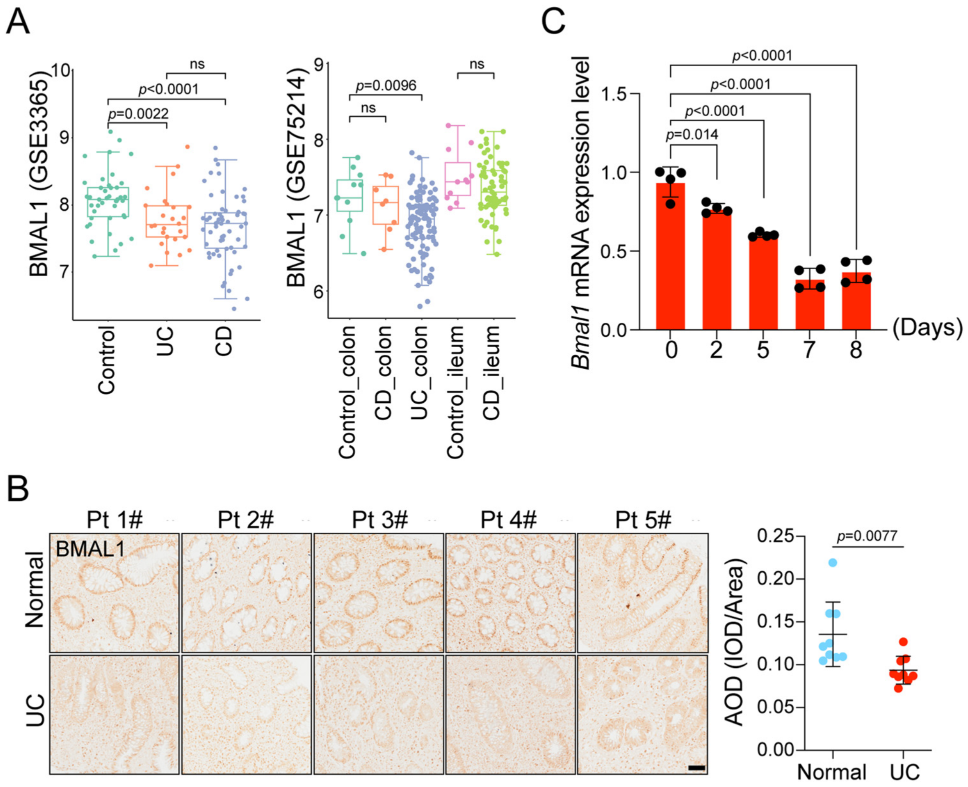

Figure EV6. BMAL1 expression is reduced in clinical UC tissues.

(**A**) Box plot showing mRNA expression of *BMAL1* in healthy and IBD patients using datasets GSE3365 (Burczynski et al, 2006; Data ref: Burczynski et al, 2006) and GSE75214 (Vancamelbeke et al, 2017; Data ref: Vancamelbeke et al, 2017). The central line represents the median. The box spans from the 25th to the 75th percentile. Whiskers extend to the smallest and largest values. All points are presented. GSE3365: Control, n = 42; UC, n = 26; CD, n = 5. GSE75214: Control_colon, n = 11; CD_colon, n = 8; UC_colon, n = 97; Control_ileum, n = 11; CD_ileum, n = 67. (**B**) Immunohistochemical analysis (left) of BMAL1 (CST, 14020S) in healthy and inflammatory regions of colonic epithelium from UC patients. Quantification of the IHC staining results by calculating the average option density (AOD) from normal and inflammatory regions of colonic epithelium from UC patients. n = 9 patient. Scale bar: 50 µm. (**C**) RT-qPCR of *Bmal1* mRNA expression in the colonic crypts of control and *Bmal1* cKO mice at different time points. n = 4 mice at different time points. Data information: Data are presented as mean ± SD, analyzed by two-tailed Student's t-test (**B**), one-way ANOVA with Tukey's multiple comparisons test (**C**). The exact *P* values are displayed. ns, no significance.

 © The Author(s)
