# Supplementary material for: Genetic disruption of the circadian gene Bmal1 in the intestinal epithelium reduces colonic inflammation
Source: EMBO Rep. 2025 Apr 30;26(12):3138–61. doi: 10.1038/s44319-025-00464-y (PMC12187941; doi:10.1038/s44319-025-00464-y)
Supplement: Supplementary file 1 — Peer Review File [file 44319_2025_464_MOESM1_ESM.pdf]

EMBO reports

Genetic disruption of the circadian gene Bmal1 in the intestinal epithelium reduces colonic inflammation

Shan Hua, Ze Zhang, Zhe Zhang, Liansheng Liu, Shicheng Yu, Yanhui Xiao, Yuan Liu, Siting Wei, Ying Xu, and Ye-Guang Chen

Corresponding author(s): Ye-Guang Chen (ygchen@tsinghua.edu.cn)

Review Timeline:

Submission Date:	7th Sep 24
Editorial Decision:	10th Oct 24
Revision Received:	28th Feb 25
Editorial Decision:	2nd Apr 25
Revision Received:	12th Apr 25
Accepted:	16th Apr 25

Editor: Achim Breiling

Transaction Report:

(Note: With the exception of the correction of typographical or spelling errors that could be a source of ambiguity, letters and reports are not edited. Depending on transfer agreements, referee reports obtained elsewhere may or may not be included in this compilation. Referee reports are anonymous unless the Referee chooses to sign their reports.)

Dear Dr. Chen,

Thank you for the submission of your manuscript to EMBO reports. I have now received the reports from the three referees that were asked to evaluate your study, which can be found at the end of this email.

As you will see, the referees find the study very interesting. Nevertheless, they have several comments, concerns, and suggestions, indicating that a major revision of the manuscript is necessary to allow publication of the study in EMBO reports. As the reports are below, and all the concerns need to be addressed, I will not detail them further here. Moreover, please cite all relevant literature (as indicated by the referees) and clearly discuss contradictions to previous publications.

Given the constructive referee comments, I would like to invite you to revise your manuscript with the understanding that the concerns of the referees must be addressed in the revised manuscript and in a detailed point-by-point response. Acceptance of your manuscript will depend on a positive outcome of a second round of review. It is EMBO reports policy to allow a single round of revision only and acceptance of the manuscript will therefore depend on the completeness of your responses included in the next, final version of the manuscript.

Revised manuscripts should be submitted within three months of a request for revision. Please contact me to discuss the revision (also by video chat) if you have questions or comments regarding the revision, or should you need additional time.

When submitting your revised manuscript, please also carefully review the instructions that follow below.

PLEASE NOTE THAT upon resubmission revised manuscripts are subjected to an initial quality control prior to exposition to re-review. Upon failure in the initial quality control, the manuscripts are sent back to the authors, which may lead to delays. Frequent reasons for such a failure are the lack of the data availability section (please see below) and the presence of statistics based on n=2 (the authors are then asked to present scatter plots or provide more data points).

When submitting your revised manuscript, we will require:

1) a .docx formatted version of the final manuscript text (including legends for main figures, EV figures and tables), but without the figures included. Figure legends should be compiled at the end of the manuscript text.

2) individual production quality figure files as .eps, .tif, .jpg (one file per figure), of main figures and EV figures. Please upload these as separate, individual files upon re-submission.

The Expanded View format, which will be displayed in the main HTML of the paper in a collapsible format, has replaced the Supplementary information. You can submit up to 5 images as Expanded View. Please follow the nomenclature Figure EV1, Figure EV2 etc. The figure legend for these should be included in the main manuscript document file in a section called Expanded View Figure Legends after the main Figure Legends section. Additional Supplementary material should be supplied as a single pdf file labeled Appendix. The Appendix should have page numbers and needs to include a table of content on the first page (with page numbers) and legends for all content. Please follow the nomenclature Appendix Figure Sx, Appendix Table Sx etc. throughout the text, and also label the figures and tables according to this nomenclature.

For more details, please refer to our guide to authors:
http://www.embopress.org/page/journal/14693178/authorguide#manuscriptpreparation

Please consult our guide for figure preparation:
http://wol-prod-cdn.literatumonline.com/pb-assets/embo-site/EMBOPress_Figure_Guidelines_061115-1561436025777.pdf

See also the guidelines for figure legend preparation:
https://www.embopress.org/page/journal/14693178/authorguide#figureformat

3) a .docx formatted letter INCLUDING the reviewers' reports and your detailed point-by-point responses to their comments. As part of the EMBO Press transparent editorial process, the point-by-point response is part of the Review Process File (RPF), which will be published alongside your paper.

4) a complete author checklist, which you can download from our author guidelines (https://www.embopress.org/page/journal/14693178/authorguide). Please insert page numbers in the checklist to indicate where the requested information can be found in the manuscript. The completed author checklist will also be part of the RPF.

Please also follow our guidelines for the use of living organisms, and the respective reporting guidelines:
http://www.embopress.org/page/journal/14693178/authorguide#livingorganisms

5) that primary datasets produced in this study (e.g. RNA-seq, ChIP-seq, structural and array data) are deposited in an appropriate public database. If no primary datasets have been deposited, please also state this in a dedicated section (e.g. 'No primary datasets have been generated and deposited'), see below.

See also: http://embor.embopress.org/authorguide#datadeposition

Please remember to provide a reviewer password if the datasets are not yet public.

The accession numbers and database should be listed in a formal "Data Availability" section that follows the model below. This is now mandatory (like the COI statement). Please note that the Data Availability Section is restricted to new primary data that are part of this study. This section is mandatory. As indicated above, if no primary datasets have been deposited, please state this in this section

Data availability

The datasets produced in this study are available in the following databases:

- RNA-Seq data: Gene Expression Omnibus GSE46843 (https://www.ncbi.nlm.nih.gov/geo/query/acc.cgi?acc=GSE46843)
- [data type]: [name of the resource] [accession number/identifier/doi] ([URL or identifiers.org/DATABASE:ACCESSION])

*** Note - All links should resolve to a page where the data can be accessed. ***

Moreover, I have these editorial requests:

6) We now request the publication of original source data with the aim of making primary data more accessible and transparent to the reader. Our source data coordinator will contact you to discuss which figure panels we would need source data for and will also provide you with helpful tips on how to upload and organize the files.

7) Our journal encourages inclusion of *data citations in the reference list* to directly cite datasets that were re-used and obtained from public databases. Data citations in the article text are distinct from normal bibliographical citations and should directly link to the database records from which the data can be accessed. In the main text, data citations are formatted as follows: "Data ref: Smith et al, 2001" or "Data ref: NCBI Sequence Read Archive PRJNA342805, 2017". In the Reference list, data citations must be labeled with "[DATASET]". A data reference must provide the database name, accession number/identifiers and a resolvable link to the landing page from which the data can be accessed at the end of the reference. Further instructions are available at: http://www.embopress.org/page/journal/14693178/authorguide#referencesformat

8) Regarding data quantification and statistics, please make sure that the number "n" for how many independent experiments were performed, their nature (biological versus technical replicates), the bars and error bars (e.g. SEM, SD) and the test used to calculate p-values is indicated in the respective figure legends (also for EV and Appendix figures). Please also check that all the p-values are explained in the legend, and that these fit to those shown in the figure. Please provide statistical testing where applicable. Please avoid the phrase 'independent experiment', but clearly state if these were biological or technical replicates. Please also indicate (e.g. with n.s.) if testing was performed, but the differences are not significant. In case n=2, please show the data as separate datapoints without error bars and statistics. See also: http://www.embopress.org/page/journal/14693178/authorguide#statisticalanalysis

If n<5, please show single datapoints for diagrams.

9) Please add scale bars of similar style and thickness to microscopic images, using clearly visible black or white bars (depending on the background). Please place these in the lower right corner of the images themselves. Please do not write on or near the bars in the image but define the size in the respective figure legend.

10) Please also note our reference format: http://www.embopress.org/page/journal/14693178/authorguide#referencesformat

11) We updated our journal's competing interests policy in January 2022 and request authors to consider both actual and perceived competing interests. Please review the policy https://www.embopress.org/competing-interests and update your competing interests if necessary. Please name this section 'Disclosure and Competing Interests Statement' and put it after the Acknowledgements section.

12) We now use CRediT to specify the contributions of each author in the journal submission system. CRediT replaces the author contribution section. Please use the free text box to provide more detailed descriptions and do NOT provide your final manuscript text file with an author contributions section. See also our guide to authors: https://www.embopress.org/page/journal/14693178/authorguide#authorshipguidelines

13) All Materials and Methods need to be described in the main text using our 'Structured Methods' format, which is required for all research articles. According to this format, the Methods section should include a Reagents and Tools Table (listing key reagents, experimental models, software, and relevant equipment and including their sources and relevant identifiers), uploaded as separate file, followed by a Methods section in which we encourage the authors to describe their methods using a step-by-step protocol format with bullet points, to facilitate the adoption of the methodologies across labs. More information on how to adhere to this format as well as downloadable templates (.doc) for the Reagents and Tools Table can be found in our author guidelines (section 'Structured Methods'):

https://www.embopress.org/page/journal/14693178/authorguide#manuscriptpreparation

14) Please provide the abstract written in present tense and order the manuscript sections like this, using these names: Title page - Abstract - Keywords - Introduction - Results - Discussion - Methods - Data availability section - Acknowledgements (including funding information) - Disclosure and Competing Interests Statement - References - Figure legends - Expanded View Figure legends

15) Please make sure that all the funding information is also entered into the online submission system and that it is complete and similar to the one in the acknowledgement section of the manuscript text file.

We would also welcome the submission of cover suggestions, or motifs to be used by our Graphics Illustrator in designing a cover.

As part of the EMBO publication's Transparent Editorial Process, EMBO reports publishes online a Review Process File (RPF) to accompany accepted manuscripts. This File will be published in conjunction with your paper and will include the referee reports, your point-by-point response and all pertinent correspondence relating to the manuscript.

You are able to opt out of this by letting the editorial office know (emboreports@embo.org). If you do opt out, the Review Process File link will point to the following statement: "No Review Process File is available with this article, as the authors have chosen not to make the review process public in this case."

I look forward to seeing a revised form of your manuscript when it is ready.

Yours sincerely,

Achim Breiling
Senior Editor
EMBO Reports

Referee #1:

The authors test the role of the circadian clock gene Bmal1 in colitis using a mouse model of disease. They find that there is a time-dependent effect of colitis damage that causes apoptosis and inflammation depending on when colitis is initiated. A conditional knock out of Bmal1 in the epithelium shows that its loss is actually protective, and it is reported that Bmal1 regulates pro-apoptotic genes. Pharmacological application to reduce Bmal1 expression at different times of day affects the degree of colitis.

The study addresses an important and interesting topic and provides some new insight into this field. It should generate interest but there are some important problems that need to be addressed to solidify the results and conclusions.

Several studies have not been cited. PMID: 33652118, and 36287037 that showed Bmal1 knock outs have increased DSS colitis. More importantly PMID: 36287037, and 38918576 that show epithelium conditional knock out of Bmal1 increases colitis disease. The paper PMID 36241650 also shows that epithelium conditional knock out of Bmal1 is detrimental to gut health. The results from these papers using very similar methods in this paper contrast with data shown in figure 2. I did not notice that these papers are cited or discussed. The authors need to discuss or resolve why their study is contradicting these previously published studies.
The results of the mouse models also contradict the clinical studies. On p5 the authors say that
"103 Analysis of public databases of human patients with inflammatory
104 bowel disease (IBD) showed that BMAL1 was downregulated in IBD patients, suggesting a
105 correlation between BMAL1 and IBD."
In figure S6 patient IBD samples are tested to show that they have low bmal1 expression. But the data in the mouse experiments shows that Bmal1 loss reduces colitis, not increases it. This contradicts the patient data and again this needs to be clearly stated and discussed in the paper.

The result that DSS gavage at ZT0 generates worse colitis than at ZT12 is very interesting. This may shed light on why the authors study is different from the published work. However, they did not measure to compare the amount of DSS consumed in figure 1 where gavage is done and figure 2 where animals drink ad libitum. It is hard to compare the ad libitum and gavage DSS, this might help resolve the problems above.

The RNA sequencing data is confusing and it isn't clear how it is relevant to the model being studied. There are some reasons for this.
168 "... we performed bulk RNA sequencing in colonic epithelial cells isolated from
169 ... control and Bmal1 KO mice"
The methods state colon crypts are collected by scraping. This method means that RNA from surrounding stroma, immune cells, etc is present in the samples and the genes detected are not specific to epithelial cells. Many of the cytokines shown in figure 3 RNA and cell analysis are not in the epithelium that confirms this contamination. It is hard to understand how this data fits tests of conditional Bmal1 knock out. A second problem is the RNA is collected not during DSS administration so the results do not show insight into the changes observed when colitis is present. In addition the time of sample collection is not given and the results may not indicate circadian rhythms in changes. I also don't see a list of the genes identified by RNA seq or Cut/tag seq in the supplementary files. The authors need to improve the methodology, analysis, and reporting of the RNA sequencing analysis to make sense of which genes are regulated by Bmal1 in the epithelium and how these affect colitis. Does the data they show in the figures agree with the publications PMID 36241650 and 38918576? These studies already did rhythmic analysis of Bmal1 regulated genes using epithelium conditional knock out but are not compared in the manuscript. The authors reanalyze published data in figure 5c-d but they need to expand this analysis outside of the handful of clock genes and apoptosis genes shown.

The organoid results showing Bmal1 conditional knock out organoids have spheroid shape and increased growth after passaging in figure 4d-g indicate a defect in differentiation not cell death. Why would organoids maintain spherical morphology due to lower cell death? It is the differentiation of organoid cells that gives rise to morphological changes as the cells differentiate up the crypt axis that give the organoid a non-spherical shape. If cells grow faster after passage this also does not indicate cell death but increased growth rate. Do organoids show rhythms in apoptosis, and would apoptotic inhibitors turn wildtypes into Bmal1 conditional knock outs? A better explanation and testing of the organoids is needed to resolve.

The data analysis in figure 5a-b is not convincing - the authors only test two samples over four timepoints in a day. There seems to be a lot of variance between replicates. Is this statistically significant? The testing of only two timepoints in figure 5e-f makes it hard to see if there is a rhythmic change. Further testing the bmal1 conditional knock out at only one timepoint does not test if apoptosis rhythms are Bmal1-dependent. The number of tests needs to be higher to make the conclusions.

There are some issues with the Reverb agonist SR9009 used in figure 6. First, the authors are assuming that the SR9009 works through Bmal1 because it inhibits Bmal1 when Reverb is agonized. This is not shown. The SR9009 is well known to have non-specific effects on Reverb itself (PMID: 31127047) and assuming it works through Bmal1 in line with the results of the study is not clear. The authors could test the Bmal1 conditional knock out with the SR9009 to show this. There are additional problems with the experimental design. Controls provided at ZT0 and ZT12 to compare with the only ZT8 control are not done. The effects of the chemical are also not straightforward because ZT0 is best then ZT12 but ZT8 in the middle of these two is worst. This is not a circadian rhythm which raises questions about the conclusions.

Statistical analysis in the methods needs to be improved.
"462 All experiments were conducted independently at least three times.
463 Each experiment contained at least two biological replicates...
463 Unless otherwise specified, statistical significance was
465 calculated using a two tailed unpaired student's t-test for two groups. The statistical analysis
466 was performed with GraphPad Prism 9.0 software, and p-values were showed as *p < 0.05,
467 **p < 0.01, ***p < 0.001, n.s, no significance."
The groups should be compared by one or two way Anova not T-tests. Exact details and p-values should be given in a supplementary table.

In many figures the time of sample collection is not indicated.

In the paper the vil-cre conditional knock out is labeled "Bmal1 KO". This should be changed since this wording is typically used in the field for the Bmal1 knock out, not a conditional knock out. This should be changed to something like "Bmal1 cKO" to match the literature in the field.

Referee #2:

In their study "Genetic disruption of the circadian gene Bmal1 in the intestinal epithelium alleviates colonic inflammation" Hue et

al. are exploring the role of the circadian clock gene Bmal1 in regulation of DSS induced colitis. The study demonstrates a circadian rhythm regulated susceptibility to DSS induced colitis that appears to be regulated by Bmal1. The study presents interesting findings in the context of colitis, but a number of points need to be addressed for this manuscript to be suitable for publication.

On a general level the, the statistical analyses need to be revised. The authors are using repeated t-tests at several places in the manuscript when they should be using a one-way ANOVA. The authors have not included any information regarding how they have assessed the cycling pattern of genes of interest. During image analysis, the authors have not calculated an average for each individual and it appears that they present each evaluated tissue section, which in my opinion is not the correct representation of that kind of data. Following revision of the statistical analysis some of the results may change, and the authors then might have to change some of their claims and interpretations throughout the paper.

Furthermore, the methods description needs to be expanded to explain the experiments in more detail. For example, there is no information regarding how the colonic epithelium was isolated.

Below you can find a point-by-point description of my specific comments.

Abstract and introduction:
Change dextrose to dextran.

Results and Figures:
Page 8: The authors need to run tests to validate that the genes they are evaluating in Fig. S1 are indeed oscillating not just making a statement that they are oscillating. They also need to support their claim of loss of oscillation in Bmal1 KO mice by statistical comparisons between groups.

Figure 1:
Fig. 1E: What comparison does the statistics refer to? I do not agree with the use of repeated t-test for data that is linked in time. The correct test to use is a two-way ANOVA. This comment also applies to 1B and C.
Fig. 1F: Please show the dots in the bar graph as you do in Fig. 1D.
Fig. 1F and 1G: The pictures are too small. Please increase the size so that it is possible to see the structure of the tissue and the immune cells. I do not agree with the representation of the data in 1G. One individual animal should be represented by one data point, not one data point per evaluated section. This type of analysis will overestimate the difference between groups. Please use the same type of bar graph as you use in 1D. Since you are comparing more than 2 groups you should use a one-way ANOVA or a non-parametric test, not multiple t-tests.

Figure 2:
Fig. 2B and C: A two-way ANOVA would be more appropriate than repeated t-tests.
Fig. 2D and F: Please show the dots in the bar graph as you do in 2E.
Fig. 2G: Same comment as for Fig. 1. There should be one dot per individual not one for each analyzed section and the pictures are too small. Please increase the size of the pictures.

Figure 3:
Fig. 3D: please show the individual dots.
Fig. 3E and F: The pictures are too small and change the data so that there is one dot per individual mouse.

Figure 4:
Fig. 4A: Pictures are too small and redo the analysis as suggested for the other images.
Fig. 4B: Show the individual data points.
What do the authors mean by bubble formation? What does % spheres refer to? To me it looks like the control spheroids are more differentiated as compared to the Bmal1 KO spheroids, as the epithelium is thicker in the control.
The authors need to add a control where they show the effect of 4-OHT on Cre- Bmal1fl/fl mice to confirm that the observed effect is related to loss of Bmal1 and not due to the 4-OHT treatment.
Fig. 4H: Same comment her as for the other analyzed images. One point per individual.

Figure 5:
Fig. 5E and F: The pictures are too small to see the apoptotic cells. Please change the graph so that one point represents one individual.
Have the authors measured villus and crypt length in the Bmal1 KO mice? If apoptosis is reduced one would expect the villi and/or crypt length to be affected.

Figure 6:
Fig. 6E and F: The pictures are too small to see the stained cells. Same comment as previously, change the graph so that one individual is represented by one point.
I think it would help the readers of this manuscript with a schematic drawing of how these experiments were done. I first thought

that mice were given DSS by gavage at the different time points in the presence of SR, and not that SR given at different time points to mice given DSS in the drinking water.

Figure S1:
The authors need to evaluate the data in Fig. 1SA for oscillating patterns not just state that they are oscillating and that oscillations are being lost in the Bmal1 KO mice. https://academic.oup.com/bioinformatics/article/32/21/3351/2415176, https://academic.oup.com/bioinformatics/article/36/3/773/5544107

Figure S2:
The pictures are too small and the statistical representation of the data needs to be changed to one point per animal.

Figure S3:
The pictures are too small in C and D. Please change the data in C to one point per individual. Is it correct that the scale bar in D is 200 µm? That would make the crypts approx. 600 µm long.
Fig. S3F: It is not clear to me what this figure represents.

Figure S4:
Please show the individual values in the bar graphs and write out the number of individual experiments.

Figure S5:
Fig. S5C and D: Please increase the size of the pictures and change the data into one point per individual.

Figure S6:
The figure legend does not match the figure. In B, use a one-way ANOVA instead of repeated t-test.

Materials and methods:

Human UC samples:
Please include information regarding the human samples. Age, gender, disease activity and treatment status. How were the human tissues stained? Did you use the same method and antibody that was used for the mouse tissue?

DSS colitis:
Line 363. If you have paraffin embedded tissues, why did you freeze the tissues?

Intestinal crypt isolation and organoid culture:
For how long were the organoids cultured prior to analysis? How was the organoid diameter analyzed? Please expand the description of how organoids were grown for the experiments and how they were analyzed.

RNA isolation and qPCR:
How were the colonic crypts isolated? Purification by TRIZOL needs more details. Table S1 shows one housekeeping gene for control and one for colitis. Does that mean that the control and DSS data were normalized against different genes? If that was the case, explain why and how that is the correct way of doing this kind of analysis.

Immunoblotting:
How was the colonic epithelium isolated?

H&E, Alcian blue, IF and IHC:
Please provide information regarding the kit that was used for H&E staining and Alcian blue. The authors have used mouse anti Il1b, IFNg and F4/80 for the analysis. Since these proteins are produced by immune cells and the authors are quantifying the number of lamina propria immune cells they cells in the lamina propria they have to provide information regarding how they have separated the real signal from the signal from the anti-mouse secondary antibody binding to lamina propria B cells.

PI staining of organoids:
Was there no washing steps added to the staining protocol?

Referee #3:

The disruption of circadian clocks has been linked to increased incidence of a range of human disorders. In this study, Hua et al. aims to investigate the functional relevance of the intestinal clock intestinal inflammation. They generated an intestinal epithelium-specific Bmal1 KO mice and examined severity of DSS-induced colitis (model for inflammatory bowel disease) in this animal. They observed that WT control mice in the active phase (ZT12) were resistant to DSS-induced colitis when compared to animals in the resting phase (ZT0). Furthermore, Bmal1 KO rendered intestinal epithelium more tolerant to DSS-induced acute

colitis, as evidenced by less weight loss, more intact epithelial barrier structure and function, as well as less infiltration of inflammatory factors and immune cells compared to control mice. They then performed RNA-seq and immunostaining to provide support that this is due to the pro-apoptotic function of BMAL1. When BMAL1 level is low (ZT12) or when it is deleted (in the BMAL1 KO), this reduced cell apoptosis, leading to the resistant phenotype. Finally, they determined the therapeutic efficacy of the REV-ERBa agonist SR9009 against DSS-induced colitis, and found that it is most effective at DSS-induced colitis at ZT0 (although it is also effective at ZT12). They conclude that targeting BMAL1 function may be a potential approach to treat inflammation-related GI disorders.

Major comments:

1. Figure 6: I understand that SR9009 has previously been shown to be an agonist for REV-ERBa. Nonetheless, they should still show BMAL1 and cleaved-caspase3 level in their SR9009 treatment samples to support that the therapeutic outcomes they observed are mediated by reduced BMAL1 and apoptosis as they predicted. This is especially important given the prior published contradictory results based on global Bmal1 KO and the fact that they also saw an effect for their ZT12+SR sample (in addition to their ZT0+SR sample).

2. Figure 5 F: The authors should provide data for ZT0 and ZT12 for both genotypes (Ctrl and KO) to highlight the loss of the rhythmicity in the KO. In addition, it will be less confusing they should use different color schemes to differentiate timepoints (ZT0 and ZT12) and genotypes (Control and KO).

3. The authors should provide quantification and statistics for all western blots and immunostaining. Only some are provided.

Minor comments:

1. I would suggest changing the first sentence in the abstract. It is awkward to suggest the clock regulates a disease. Perhaps just it to "Disruption of the circadian clock increases the risk (or incidence) of IBD.

2. Line 37: "injure" should be "injury".

3. Define abbreviations the first time you use it in text of figure. For example, line 137, H&E. In figure legends, define SI and LI (small intestine and large intestine).

4. Line 160: define what you mean by "better structure", or include citations that define better structure.

5. Line 189: Please elaborate why only the overlapping 495 genes are potential Bmal1-regulated genes? Why aren't all genes that were identified to be BMAL1-bound considered to be Bmal1-regulated genes?

6. Line 205: indicate dead cells are stained by propidium iodide.

Summary of Figure Changes in the Revised Manuscript

Revised version	Previous version	Description
Fig. 1B-E	Figure 1B-E	Change statistical analyses to two-way ANOVA
Fig. 1F	Figure 1F	Increase the size of the pictures<br>Show the dots in the bar graph<br>Change statistical analyses to one-way ANOVA
Fig. 1G	Figure 1G	Increase the size of the pictures<br>Show the dots in the bar graph<br>Represent one individual animal by one data point<br>Change statistical analyses to one-way ANOVA
Fig. 2B-C	Figure 2B-C	Change statistical analyses to two-way ANOVA
Fig. 2D	Figure 2D	Show the dots in the bar graph<br>Represent one individual animal by one data point
Fig. 2E	Figure 2E	Increase the size of the pictures<br>Show the dots in the bar graph<br>Represent one individual animal by one data point
Fig. 2F	Figure 2F	Show the dots in the bar graph<br>Represent one individual animal by one data point
Fig. 2G-H	Figure 2G-H	Increase the size of the pictures<br>Represent one individual animal by one data point
Fig. 3D	Figure 3D	Show the dots in the bar graph<br>Represent one individual animal by one data point
Fig. 3E-F	Figure 3E-F	Increase the size of the pictures<br>Represent one individual animal by one data point
Fig. 4A	Figure 4A	Increase the size of the pictures<br>Represent one individual animal by one data point
Fig. 4B	Figure 4B	Show the dots in the bar graph<br>Represent one individual animal by one data point
Fig. 4C	Figure 4C	Quantitative western-blot by ImageJ
Fig. 4D		Add the schematic diagram of organoid processing
Fig. 4E		New data
Fig. 4F		New data
Fig. 4G	Figure 4D	Add quantifications and statistics for PI intensity
Fig. 4H	Figure 4E	Add quantifications and statistics for ratio of cleaved-caspase cells (%)
Fig. 4I	Figure 4F	Replaced with the new data
Fig. 4J	Figure 4G	Replaced with the new data
Fig. 4K	Figure 4H	Add quantifications for western-blot
Fig. 5A		New data
Fig. 5B	Figure 5A	Replaced with the new data

Fig. 5C	Figure 5B	Replaced with the new data
Fig. 5D		New data
Fig. 5E	Figure 5C	Replace with the new data
Fig. 5F	Figure 5D	Replace with the new data
Fig. 5G	Figure 5E-F	New data
Fig. 6A-B		New data
Fig. 6C		Add the schematic diagram of SR9009-treatment
Fig. 6D-F	Figure 6A-C	Replace with the new data
Fig. 6G		New data
Fig. 6H-J	Figure 6D-F	Replace with the new data
Fig. EV1A	Supfigure 1A	Add statistics
Fig. EV2B		New data
Fig. EV2C	Supfigure 2B	Add statistics on the number of KI67$^+$ cells in the colonic crypts<br>Represent one individual animal by one data point
Fig. EV2D-E	Supfigure 2C-D	Represent one individual animal by one data point
Fig. EV2E	Supfigure 2D	Represent one individual animal by one data point
Fig. EV2F	Supfigure 2E	Add statistics on the number of Ab$^+$ cells in the colonic crypts<br>Represent one individual animal by one data point
Fig. EV2G	Supfigure 2F	Add statistics on the number of Chga$^+$ cells in the colonic crypts<br>Represent one individual animal by one data point
Fig. EV3C-D	Supfigure 3C-D	Increase the size of the pictures<br>Represent one individual animal by one data point
Fig. EV4A-D	Supfigure 4A	Replace with the new data
Fig. EV5A-C	Supfigure 5A-D	Replace with the new data
Fig. EV6C	Supfigure 6C	Change statistical analyses to one-way ANOVA

Referee #1:

The authors test the role of the circadian clock gene Bmal1 in colitis using a mouse model of disease. They find that there is a time-dependent effect of colitis damage that causes apoptosis and inflammation depending on when colitis is initiated. A conditional knock out of Bmal1 in the epithelium shows that its loss is actually protective, and it is reported that Bmal1 regulates pro-apoptotic genes. Pharmacological application to reduce Bmal1 expression at different times of day affects the degree of colitis.

The study addresses an important and interesting topic and provides some new insight into this field. It should generate interest but there are some important problems that need to be addressed to solidify the results and conclusions.

1. Several studies have not been cited. PMID: 33652118, and 36287037 that showed Bmal1 knock outs have increased DSS colitis. More importantly PMID: 36287037, and 38918576 that show epithelium conditional knock out of Bmal1 increases colitis disease. The paper PMID 36241650 also shows that epithelium conditional knock out of Bmal1 is detrimental to gut health. The results from these papers using very similar methods in this paper contrast with data shown in figure 2. I did not notice that these papers are cited or discussed. The authors need to discuss or resolve why their study is contradicting these previously published studies.

Response: We appreciate your insightful comments. As discussed in our manuscript **(page 15, lines 288-298),** we noticed that previous studies have reported increased colitis susceptibility in global *Bmal1* knockout mice (including PMID: 33652118). However, accumulating evidence indicates significant tissue-specific effects of *Bmal1* deletion. Global *Bmal1*-deficient mice exhibit age-associated dilated cardiomyopathy, with dysfunction of left ventricular dilatation and contraction (Lefta *et al*, 2012). Adipocyte-specific deletion of *Bmal1* leads to obesity in mice for the disrupted food intake rhythm (Paschos *et al*, 2012), whereas intestinal epithelial-specific *Bmal1* deletion reduces high-fat diet-induced obesity (Yu *et al*, 2021). These reports suggest that the global knockout of circadian genes may not be appropriate to characterize clock function in specific tissues. These could explain the differing colitis phenotypes observed in global and intestinal epithelial-specific knockout of *Bmal1*. We have cited relevant studies in the revised Discussion section **(page 15, lines 288-298).**

To address this, we employed *Villin-CreERT2; Bma1*$^{fl/fl}$ with tamoxifen-inducible deletion with specific target to the intestinal epithelium. In contrast, PMID: 36287037 and 38918576 used *TS4-Cre; Bmal1*$^{fl/fl}$ and *Villin-Cre; Bmal1*$^{fl/fl}$ mice, one with a different specific knockout site (Villin in our case), and both constitutive knockouts that lack temporal control and could involve developmental confounding. It is difficult to rule out developmental effects of knockouts in *Cre* mice, while potential effects in *Cre* mice may occur during continued knockouts. These methodological differences are consistent with recent findings (including PMID: 36241650) demonstrating that chronic epithelial *Bmal1* deletion impairs gut homeostasis. Thus, we believe these differences explain the divergence between our conclusions and those of previous studies. We have cited the relevant work and

further elaborated on these points in the Discussion section **(page 16, lines 299-302)**.

2. The results of the mouse models also contradict the clinical studies. On p5 the authors say that "Analysis of public databases of human patients with inflammatory bowel disease (IBD) showed that BMAL1 was downregulated in IBD patients, suggesting a correlation between BMAL1 and IBD." In figure S6 patient IBD samples are tested to show that they have low bmal1 expression. But the data in the mouse experiments shows that Bmal1 loss reduces colitis, not increases it. This contradicts the patient data and again this needs to be clearly stated and discussed in the paper.

Response: As we discussed in the Discussion section **(page 17, lines 329-336)**, the decreased BMAL1 expression in IBD patients could be an adaptation of the intestine to better regenerate the damaged tissue in the context of inflammation. The suppression of circadian clock was also observed in other disorders. For example, it has been reported that disruption of SCN function alleviated myocardial infarction induced cardiac dysfunction and cardiac fibrosis and the lethality of temperature imbalance in mice caused by time-restricted feeding treatment (Hao et al, 2023; Zhang et al, 2020). These observations suggest that mice may be able to rapidly respond to some stress conditions by inhibiting the circadian clock, thereby better performing the repair process.

3. The result that DSS gavage at ZT0 generates worse colitis than at ZT12 is very interesting. This may shed light on why the authors study is different from the published work. However, they did not measure to compare the amount of DSS consumed in figure 1 where gavage is done and figure 2 where animals drink ad libitum. It is hard to compare the ad libitum and gavage DSS, this might help resolve the problems above.

Response: Thank you for your insightful suggestion. As detailed in the Methods section **(page 19, lines 362-388)**, we employed two distinct DSS-induced colitis models to address specific research objectives. To assess the impact of circadian clock and time on colitis, we induced colitis by gavaging mice with high doses of DSS solution during the early resting period (ZT0) and early active period (ZT12). In contrast, to investigate the role of the circadian gene *Bmal1* in colitis, we challenged control mice and *Bmal1* cKO mice by drinking 3% DSS solution ad libitum using a more commonly used method. Given their fundamentally different purposes (circadian phase assessment vs gene function analysis), direct comparison of DSS consumption between these methodologies would not yield biologically meaningful insights. We separately mentioned the two experiments to avoid possible confusions.

4. The RNA sequencing data is confusing and it isn't clear how it is relevant to the model being studied. There are some reasons for this.

168 "... we performed bulk RNA sequencing in colonic epithelial cells isolated from control and Bmal1 KO mice"

The methods state colon crypts are collected by scraping. This method means that RNA from surrounding stroma, immune cells, etc is present in the samples and the genes detected are not specific to epithelial cells. Many of the cytokines shown in figure 3 RNA and cell analysis are not in the epithelium that confirms this contamination. It is hard to understand

how this data fits tests of conditional Bmal1 knock out. A second problem is the RNA is collected not during DSS administration so the results do not show insight into the changes observed when colitis is present. In addition the time of sample collection is not given and the results may not indicate circadian rhythms in changes. I also don't see a list of the genes identified by RNA seq or Cut/tag seq in the supplementary files. The authors need to improve the methodology, analysis, and reporting of the RNA sequencing analysis to make sense of which genes are regulated by Bmal1 in the epithelium and how these affect colitis. Does the data they show in the figures agree with the publications PMID 36241650 and 38918576? These studies already did rhythmic analysis of Bmal1 regulated genes using epithelium conditional knock out but are not compared in the manuscript. The authors reanalyze published data in figure 5c-d but they need to expand this analysis outside of the handful of clock genes and apoptosis genes shown.

Response: We appreciate your valuable suggestions. For colonic crypt isolation, we scraped and filtered samples through a 70 µm membrane to remove most stromal cells, isolating primarily epithelial cells. We have provided a more detailed description in the Methods section **(page 20, lines 389-395)**.

We performed the DWLS deconvolution (Avila Cobos *et al*, 2020; Tsoucas *et al*, 2019) to quantify cell proportions in our RNA-seq data. We found that in the samples isolated from control mice, the proportions of six epithelial cell types (EEC, Enterocyte, Goblet_cell, Stem_cell, TA_cell, and Tuft_cell) reached 80.86% and 93.17%, respectively, while in the cKO samples, the proportions were both around 99% of epithelial cells **(**see the following **Response table 1)**, indicating that our samples are the epithelial tissues. While some immune cells and other cells may be present in isolated crypts, these do not affect our conclusions. Specifically, *Bmal1* cKO mice exhibited reduced inflammation and immune cell infiltration in the colon after DSS treatment, likely due to that decreased epithelial apoptosis preserves the epithelial barrier and limits immune infiltration. Consistently, we performed CUT&Tag experiments with wild-type organoids, demonstrating that BMAL1 binds to the promoters of apoptosis-related genes and regulates their expression in intestinal epithelial cells.

Cell ratio	Control 1#	Control 2#	cKO 1#	cKO 2#
EEC	3.84%	2.97%	0.50%	0.46%
Enterocyte	28.23%	37.13%	47.04%	41.55%
Goblet_cell	12.64%	19.38%	9.68%	14.14%
Stem_cell	0.00%	0.00%	15.56%	18.66%
TA_cell	35.19%	32.69%	27.14%	24.42%
Tuft_cell	0.96%	1.00%	0.07%	0.14%
**Total**	80.86%	93.17%	99.99%	99.37%
B_cell	6.29%	0.00%	0.01%	0.63%
Macrophage	4.86%	6.26%	0.00%	0.00%
myoFibroblast	0.23%	0.00%	0.00%	0.00%

Response table 1. **DWLS deconvolution to quantify cell proportions in control and *Bmal1* cKO mice at day 7 following DSS treatment.**

Regarding the RNA collection time, samples were collected on day 7 of DSS treatment, which corresponds to the peak of the colitis phenotype **(Figure 2)**. We believe this time point captures more pronounced transcriptional differences between wild-type and *Bmal1* cKO mice. The relevant RNA-seq gene expression list and CUT&Tag Seq list are listed in Dataset EV1-3. To further address your comments, we have isolated DAPI$^-$ Epcam$^+$ epithelial cells from control and *Bmal1* cKO mice at day 5 following DSS treatment and performed RNA-seq analysis. These results revealed a higher ratio of intestinal epithelial cells in *Bmal1* cKO mice at day 5 following DSS treatment, suggesting less immune cell infiltration **(Response Figure 1A)**. GO analysis revealed that the majority of down-regulated genes in *Bmal1* cKO mice were enriched in cell death and the apoptotic pathway **(Response Figure 1B)**. Consistently, genes involved in apoptotic signaling were greatly downregulated upon *Bmal1* cKO **(Response Figure 1C, D)**. These results indicate that BMAL1 can transcriptionally activate the expression of apoptosis-related genes in the DSS-treated intestine.

We have reviewed the two recommended studies (PMID: 36241650 and 38918576) and found that they did not provide relevant transcriptomic data. PMID: 36241650 employed a *Villin-Cre* constitutive knockout model, which differs from our *Villin-CreERT2* inducible model. The *Villin-Cre* model introduces potential developmental defects due to continuous knockout. Finally, we reanalyzed the published data in Figure 5C-D and confirmed that the transcriptomes of small intestinal epithelial cells exhibit rhythmicity.

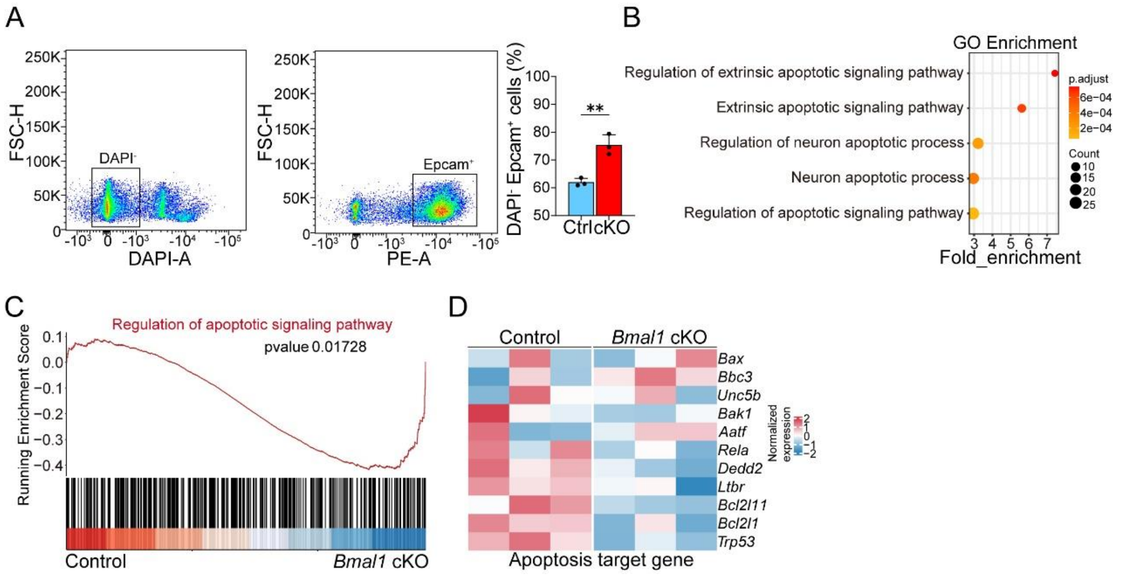

Response Figure 1. **Cell apoptosis is attenuated in *Bmal1*-deficient intestinal epithelium.** (**A**) FACS gating strategy of DAPI$^-$ Epcam$^+$ epithelial cells (left) and DAPI$^-$ Epcam$^+$ cells were quantified (right) from control and *Bmal1* cKO mice after DSS treatment at day 5 following DSS treatment. Plots are shown from a representative sample. (**B**) GO enrichment of downregulated genes in the DAPI$^-$ Epcam$^+$ epithelial cells of *Bmal1* cKO mice at day 5 following DSS treatment. (**C**) GSEA of apoptosis gene sets enriched with decreased genes in DAPI$^-$ Epcam$^+$ epithelial cells from *Bmal1* cKO mice at day 5 following DSS treatment. (**D**) Heatmap showing the expression of the apoptosis related genes in

DAPI- Epcam+ epithelial cells from control and *Bmal1* cKO mice at day 5 following DSS treatment. Data are presented as mean ± SD with statistical analyses determined by two-tailed Student's t-test. **$p < 0.01$.

5. The organoid results showing Bmal1 conditional knock out organoids have spheroid shape and increased growth after passaging in figure 4d-g indicate a defect in differentiation not cell death. Why would organoids maintain spherical morphology due to lower cell death? It is the differentiation of organoid cells that gives rise to morphological changes as the cells differentiate up the crypt axis that give the organoid a non-spherical shape. If cells grow faster after passage this also does not indicate cell death but increased growth rate. Do organoids show rhythms in apoptosis, and would apoptotic inhibitors turn wildtypes into Bmal1 conditional knock outs? A better explanation and testing of the organoids is needed to resolve.

Response: Thank you for your insightful comments and suggestions. We observed that *Bmal1* cKO organoids exhibit a bubbing phenotype, which is accompanied by a decrease in apoptotic cells within the cavity, which may attributable to the suppression of differentiated cell populations (including MUC2^{+} goblet cells and CHGA^{+} enteroendocrine cells), decreased cell death and but not cell proliferation **(Response Figure 2, new Fig. EV4)**. Furthermore, we observed that the addition of the apoptosis inhibitor Z-VAD-FMK to cultured wild-type organoids in vitro did not induce bubble formation (**Response Figure 3**).

Previous studies have reported that the transcriptome of intestinal organoids cultured in vitro have no rhythmic expression (Rosselot *et al*, 2022). We have cited it in the text **(page16 , line 307-309)**.

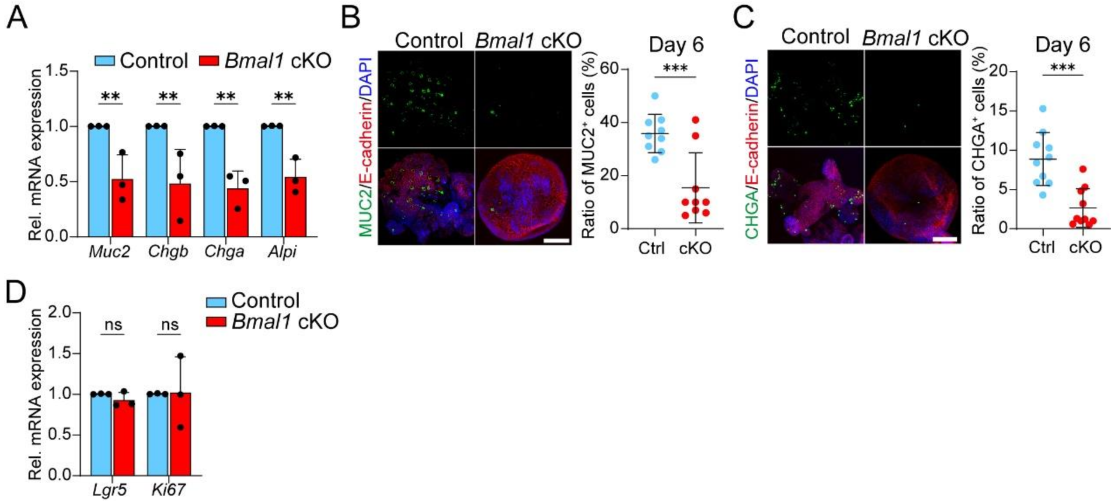

Response Figure 2. **Ablation of *Bmal1* suppresses differentiation but does not affect stemness and proliferation in vitro.** (**A**). RT-qPCR analysis of the mRNA expression of *Muc2*, *Chgb*, *Chga* and *Alpi* from control organoids and *Bmal1* cKO organoids on day six. n=3 independent experiments. (**B**, **C**) Immunofluorescence staining for MUC2 (**B**) and CHGA (**C**) and quantification of MUC2^{+} and CHGA^{+} cells (right) from control organoids and *Bmal1* cKO organoids. n=10 organoids from 3 independent experiments. Scale bar: 100μm. (**D**) RT-qPCR analysis for the mRNA expression of *Lgr5* and *Ki67* in control and *Bmal1* cKO organoids at day 3 starting at single cells. n=3 independent experiments. Data

are presented as mean ± SD with statistical analyses determined by two-tailed Student's t-test (**B**, **C**) and two-way ANOVA with Tukey's multiple comparisons test (**A**, **D**). **p < 0.01, n.s, no significance.

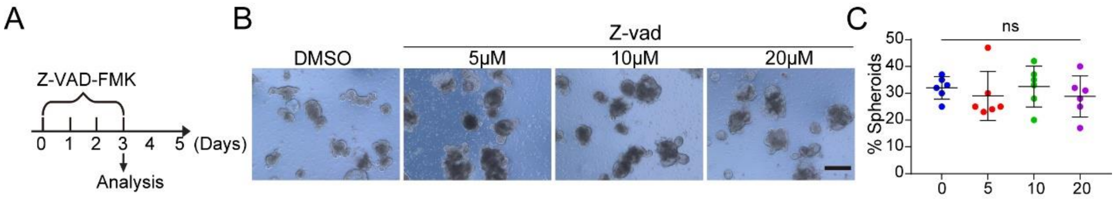

Response Figure 3 **Inhibition of apoptosis does not affect organoid budding.** (**A**) Schematic showing wild-type colonic organoids treated with Z-VAD-FMK. (**B**) Organoids derived from wild-type mice treated with Z-VAD-FMK. Images were taken on day three. Scale bar: 400μm. (**C**) Bubble formation rate was counted from six fields of view from three independent experiments. Data are presented as mean ± SD with statistical analyses determined by one-way ANOVA with Tukey's multiple comparisons test (**A**, **D**). n.s, no significance.

6. The data analysis in figure 5a-b is not convincing - the authors only test two samples over four timepoints in a day. There seems to be a lot of variance between replicates. Is this statistically significant? The testing of only two timepoints in figure 5e-f makes it hard to see if there is a rhythmic change. Further testing the bmal1 conditional knock out at only one timepoint does not test if apoptosis rhythms are Bmal1-dependent. The number of tests needs to be higher to make the conclusions.

Response: Thanks for your suggestions. We have collected additional samples at the indicated time point (n=3 for each group) for RNA sequencing and also detected cleaved-caspase3 protein expression levels in wild-type and *Bmal1* cKO mice across a broader time range. The colonic crypts were collected from wild-type mice sacrificed at ZT0, ZT6, ZT12 and ZT18 for RNA-sequencing and transcriptomes were analyzed by MetaCycle (De Los Santos *et al*, 2020; Wu *et al*, 2016). Heatmap showed that a portion of genes exhibited a rhythmic expression pattern **(Response Figure 4A, new Fig. 5A)**. Gene set variation analysis (GSVA) showed that the expression levels of the apoptosis-related genes exhibited rhythmic oscillations, with a higher level at ZT0-ZT6 and lower level at ZT12 **(Response Figure 4B, new Fig. 5B)**. The expression of the core apoptotic genes, such as *Bax* and *P53*, had a similar pattern **(Response Figure 4C, new Fig. 5C)**. Analysis of the public transcriptome data in the small intestinal epithelium also revealed that a portion of genes, especially apoptosis-related genes, exhibited a rhythmic expression pattern **(Response Figure 4D-F, new Fig. 5D-F)**. Consistently, the cleaved-caspase 3 signals had a rhythmic pattern with the highest level at ZT0 and lowest at ZT12 in the proximal small intestine and distal large intestine, and *Bmal1* cKO led to unchanged low cleaved-caspase 3 levels **(Response Figure 4G, new Fig. 5G)**. These results indicate that cell apoptosis exhibits a *Bmal1*-dependent circadian rhythm pattern in the intestinal epithelium. We have now updated the data in new **Figure 5**.

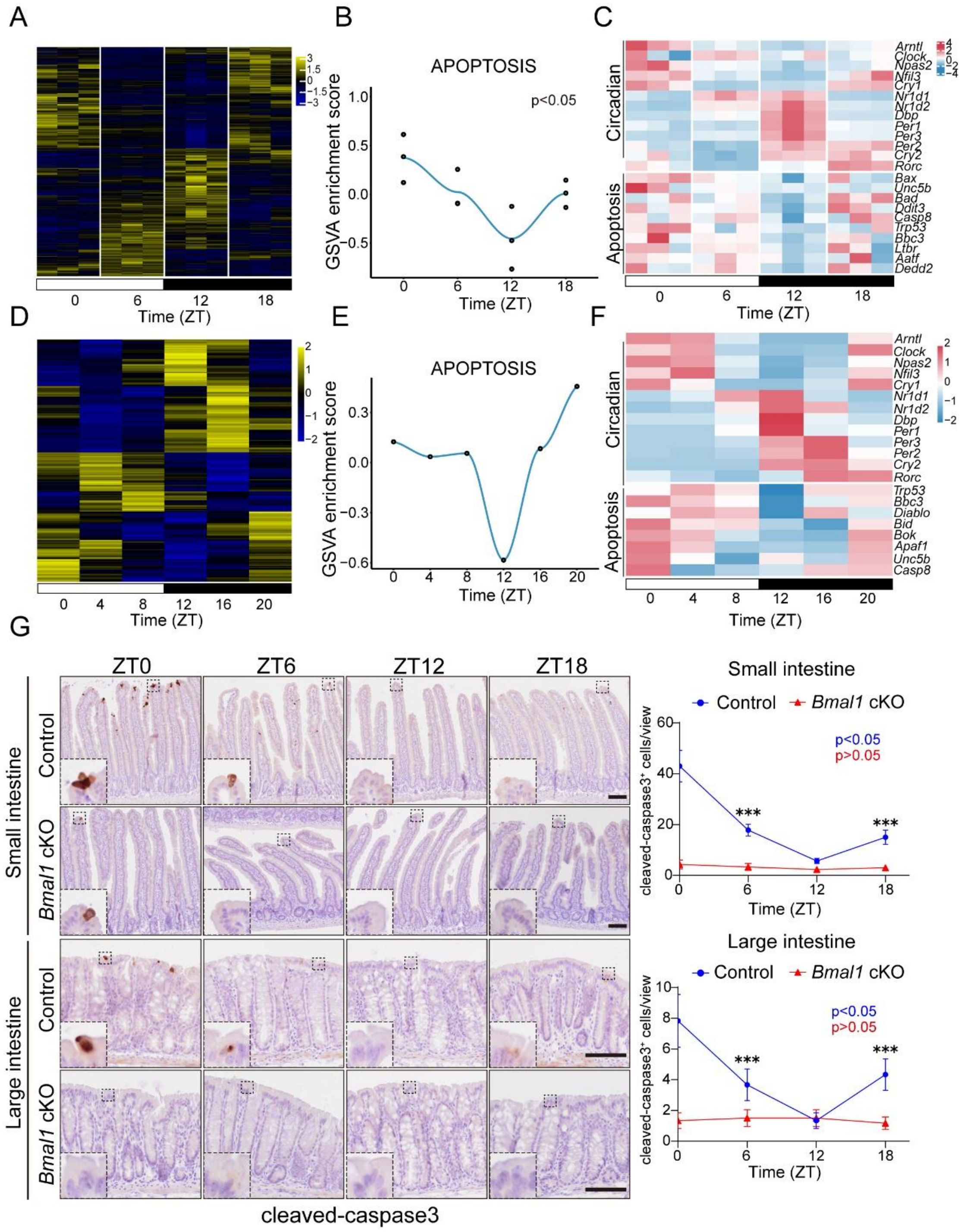

Response Figure 4 **Cell apoptosis occurs in a circadian rhythm-dependent manner in the intestinal epithelium.** (**A**) Heatmap showing gene expression ordered by MetaCycle phase to depict all significant circadian genes in colonic crypts from the wild-type mice. n=3 mice for each group. (**B**) GSVA score for apoptosis gene sets enriched in colonic crypts obtained from the wild-type mice at indicated time point. Individual points represent the enrichment score for each sample. n=3 mice for each group. (**C**) Heatmap showing the expression of circadian genes and apoptosis-related genes in the colonic crypts from wild-type mice at indicated time point. n=3 mice for each group. (**D**) Heatmap showing gene expression ordered by MetaCycle phase to depict all significant circadian genes in

small intestinal epithelium from the wild-type mice using the database GSE100339. (**E-F**) GSVA score for apoptosis gene sets enriched (**E**) and heatmap (**F**) showing the expression of circadian genes and apoptosis related genes in small intestinal epithelium from wild-type mice at indicated time point using the database GSE100339. (**G**) Immunohistochemical analysis of cleaved-caspase3 (left) and quantification (right) in proximal small intestine (top) and distal large (bottom) intestine sections from control mice and *Bmal1* cKO mice at ZT0, ZT6, ZT12 and ZT18. Scale bar: 100μm. n=6 mice for each group at indicated time point. Data are presented as mean ± SD. The rhythmicity of the oscillating pattern was measured by the JTK cycle through the MetaCycle R package. With the settings of Period=24 h and adj.p < 0.05, expression patterns were then defined as rhythmic. Two-way ANOVA with Tukey's multiple comparisons test was also used (**G**). ***$p < 0.001$.

7. There are some issues with the Reverb agonist SR9009 used in figure 6. First, the authors are assuming that the SR9009 works through Bmal1 because it inhibits Bmal1 when Reverb is agonized. This is not shown. The SR9009 is well known to have non-specific effects on Reverb itself (PMID: 31127047) and assuming it works through Bmal1 in line with the results of the study is not clear. The authors could test the Bmal1 conditional knock out with the SR9009 to show this. There are additional problems with the experimental design. Controls provided at ZT0 and ZT12 to compare with the only ZT8 control are not done. The effects of the chemical are also not straightforward because ZT0 is best then ZT12 but ZT8 in the middle of these two is worst. This is not a circadian rhythm which raises questions about the conclusions.

Response: In response to your comments, we first investigated the effects of SR9009 on *Bmal1* expression in organoids and observed a decreased *Bmal1* expression **(Response Figure 5A, new Fig. 6A)**. Cleaved-caspase 3 staining revealed that both SR9009-treated and *Bmal1* cKO organoids exhibited significantly reduced apoptotic cell populations compared to control organoids **(Response Figure 5B, new Fig. 6B)**. Furthermore, decreased BMAL1 and cleaved-caspase 3 levels were detected in SR9009-treated mice at ZT0 **(Response Figure 5C, new Fig. 6J)**. These findings demonstrate that SR9009 alleviates intestinal inflammation through suppressing of *Bmal1* expression and inhibition of apoptotic pathways.

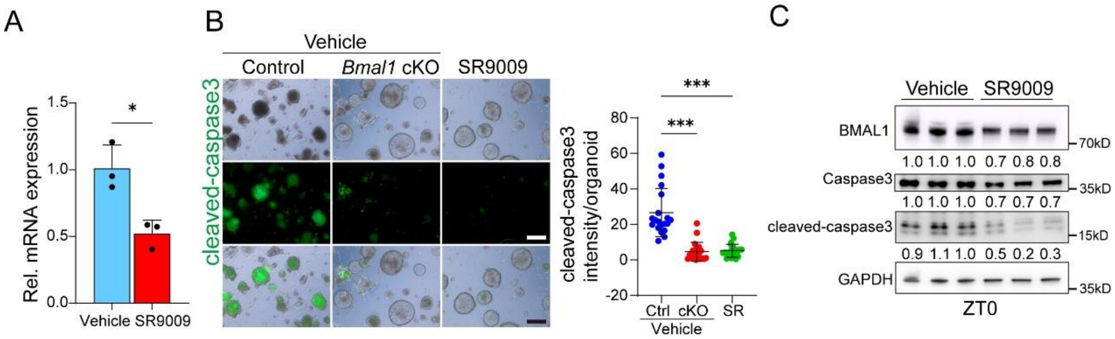

Response Figure 5 **SR9009 inhibits *Bmal1* expression and cell apoptosis.** (**A**) RT-qPCR analysis of the mRNA expression of *Bmal1* from control colonic organoids and

SR9009-treated organoids. n=3 independent experiment. (**B**) Representative brightfield (top), cleaved-caspase 3 staining (bottom) and intensity quantification (right) from control colonic organoids, *Bmal1* cKO organoids and SR9009 treated-colonic organoids. Scale bar: 400μm. n=10 organoids from three independent experiments for each group. (**C**) Western blotting for BMAL1, cleaved-caspase 3, caspase 3 in the colonic crypts of control and SR9009-treated mice after DSS treatment at ZT0. GAPDH was used as loading control. n=3 mice for each group. Data are presented as mean ± SD. The data were analyzed by two- tailed Student's t-test (**A**), one-way ANOVA with Tukey's multiple comparisons test (**B**). $*p < 0.05$, $***p < 0.001$.

Furthermore, we found some critical limitations in our previous experimental design. In the initial protocol, mice were allowed free access to DSS solution starting from ZT8 for five consecutive days, with SR9009 administered intraperitoneally at ZT0, ZT8, and ZT12 on day 3. Following DSS replacement with water at ZT8 on day 5, tissue collection was performed at ZT0, ZT8, and ZT12 on day 8. This design introduced a significant confounding factor, as the duration of DSS exposure varied among experimental groups, potentially compromising the interpretation of results due to differential modeling periods. In our new experimental design, wild-type mice were given DSS solution ad libitum for five days at ZT0, ZT8, and ZT12, respectively, and switched to water at ZT0, ZT8, and ZT12 on the fifth day to ensure that each group of mice consumed DSS drinking water for five days **(Response Figure 6A, new Fig. 6C)**. To ensure consistent treatment duration across all experimental groups, SR9009 administration was initiated on day 3 through intraperitoneal injections at ZT0, ZT8, and ZT12, maintaining this regimen for five consecutive days in DSS-treated mice. Tissue collection was subsequently performed at ZT0, ZT8, and ZT12 on day 8, thereby standardizing both the SR9009 treatment period and circadian sampling points across all experimental conditions. We consider this modified experimental design to be more scientifically rigorous, as it effectively controls for both treatment duration and circadian time variables, thereby minimizing potential confounding factors and enhancing the reliability of experimental outcomes.

Under these conditions, mice treated with SR9009 at ZT0 exhibited superior therapeutic outcomes, evidenced by less body weight loss, lower disease activity index, higher survival rates and longer colon lengths, compared to those treated at other time points, with ZT12 being the least effective **(Response Figure 6B-E; Response Figure 7A, new Fig. 6D-G; new Fig. EV5A)**. Histochemical staining showed that SR9009 treatment at ZT0 had the lowest histological scores **(Response Figure 6F, G, new Fig. 6H)**. Furthermore, fewer apoptotic cells and decreased BMAL1 and cleaved-caspase3 levels were detected in SR9009 treated mice at ZT0 **(Response Figure 6H, new Fig. 6I)**. Consistently, mice treated at ZT0 had significantly reduced infiltration of immune $CD45^+$ cells and lower expression levels of inflammatory protein IL-1β **(Response Figure 7B, C, new Fig. EV5B, C)**. Collectively, these results indicate that SR9009 treatment at ZT0 has the best beneficial efficacy in DSS-induced colitis. Our experiments demonstrate the rhythmic therapeutic effect of SR9009, which reduces *Bmal1* expression and further lowers apoptosis levels, providing a therapeutic benefit in colitis.

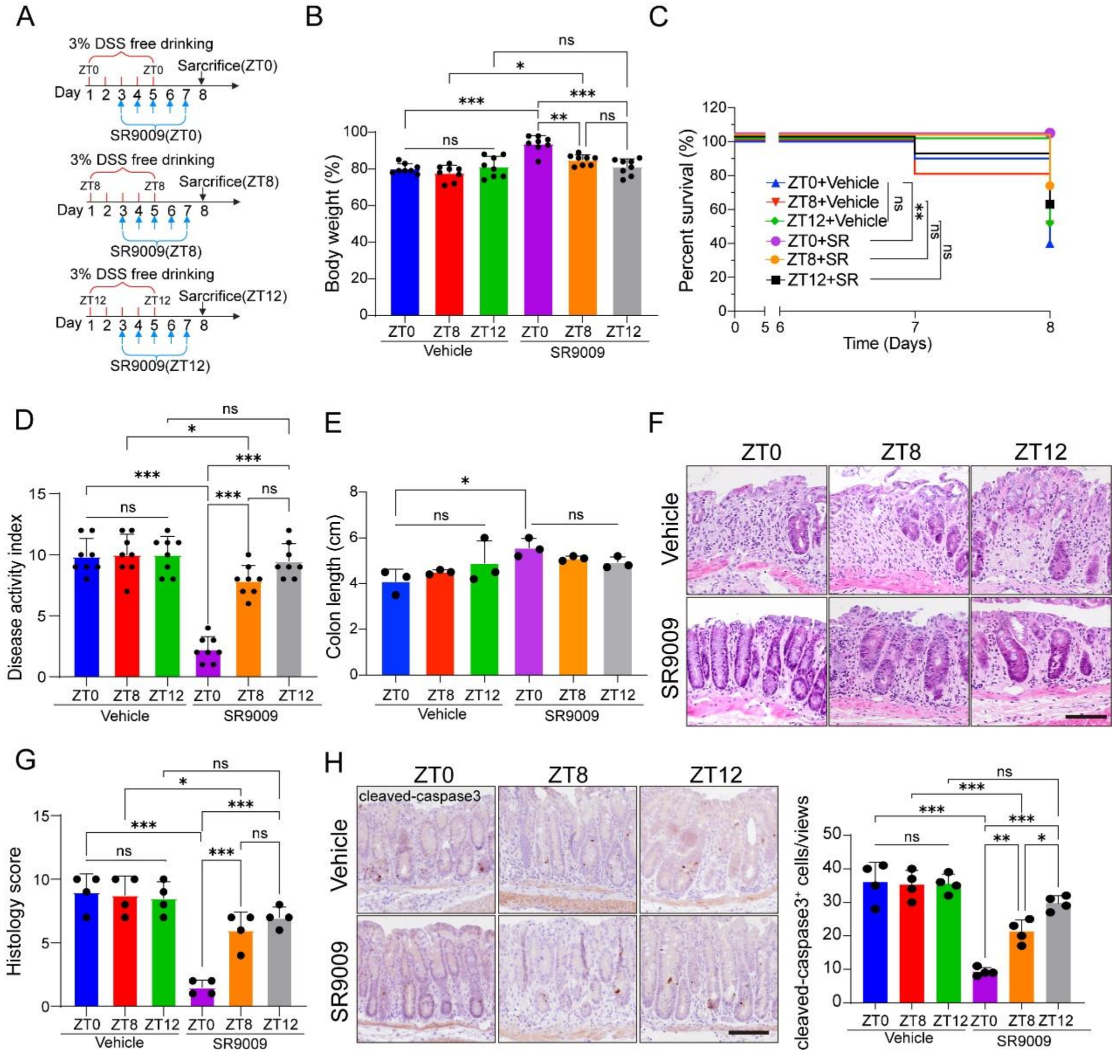

Response Figure 6 **Inhibition of *Bmal1* expression at ZT0 achieves the best efficacy in DSS-induced colitis.** (**A**) Schematic diagram showing SR9009 administration during colitis induction. Wild-type mice were given DSS solution ad libitum for five days at ZT0, ZT8, and ZT12, respectively, and switched to water at ZT0, ZT8, and ZT12 on the fifth day to ensure that each group of mice consumed DSS drinking water for five days. SR9009 (50mg/kg) was administered intraperitoneally once daily for 5 consecutive days at ZT0, ZT8, and ZT12, respectively, starting on the third day of DSS-induced colitis in mice. Colon tissues were subsequently collected at the day 8. Control group intraperitoneal injection of vehicle. (**B**-**D**) Body weight change (**B**), survival curve (**C**) and disease activity index (**D**) of wild-type mice after DSS treatment. SR9009 (50mg/kg) was administered intraperitoneally once daily for 5 consecutive days at ZT0, ZT8, and ZT12, respectively, starting on the third day of DSS-induced colitis in mice. Mice were injected intraperitoneally with the vehicle as a control group at ZT0, ZT8 and ZT12. n=8/10 mice for each group from two independent experiments. (**E**) Colon length quantification from six groups of mice. Colon tissues were collected at the day 8. n=3 mice for each group Scale bar: 100μm. (**F**, **G**) Representative H&E stanning (**F**) images of the distal colon sections, and histological scores (**G**) were obtained from six groups of mice. Colon tissues were

collected at the day 8. n=4 mice for each group Scale bar: 100μm. (**H**) Immunohistochemical analysis of cleaved-caspase3 (left) and quantification (right) in distal colon sections from six groups. Colon tissues were collected at the day 8. Scale bar: 100μm. n=4 mice for each group. Data are presented as mean ± SD. The data were analyzed by one-way ANOVA with Tukey's multiple comparisons test (**B**, **D**, **E**, **G**, **H**) and one-sided log-rank (**C**). *p < 0.05, **p < 0.01, ***p < 0.001.

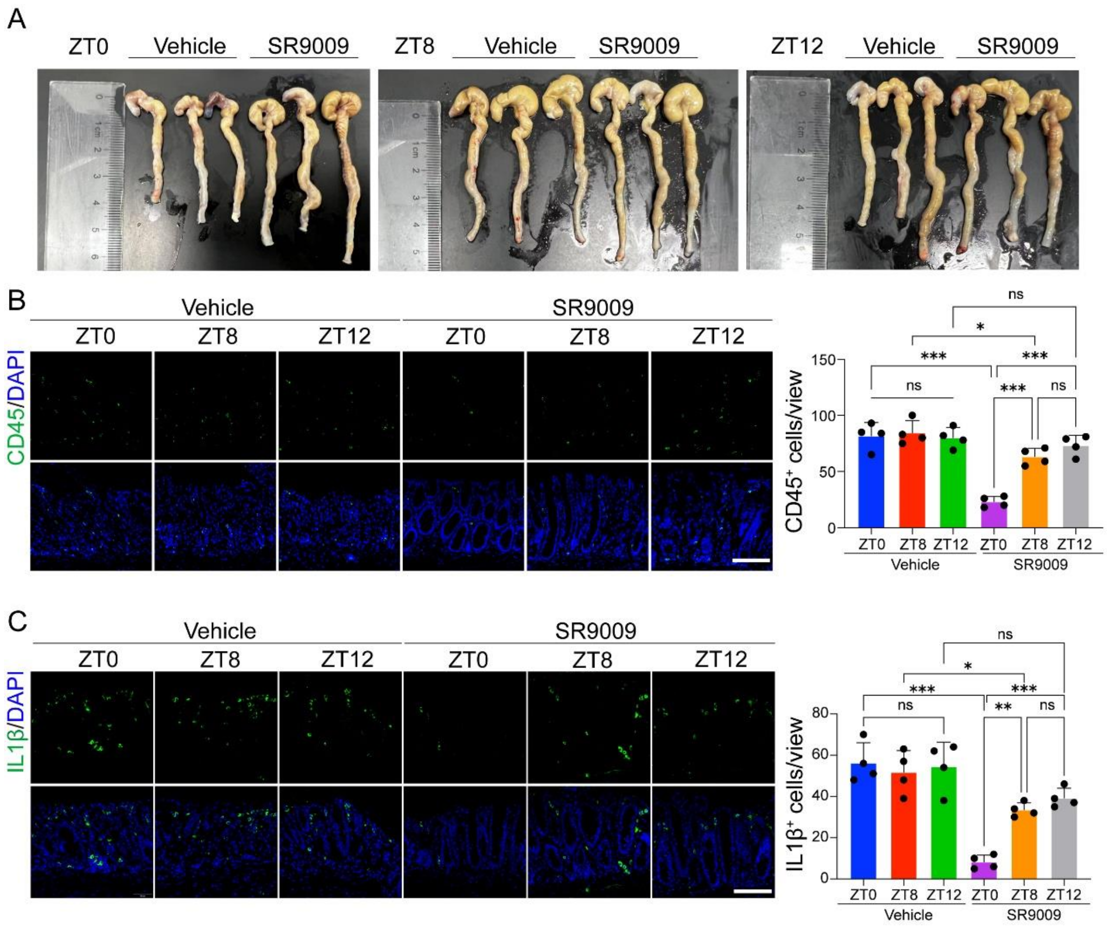

Response Figure 7. **Mice treated with SR9009 at ZT0 have less immune cell infiltration.** (**A**) Colon length from six groups of mice. (**B**, **C**). Immunofluorescence staining for CD45 (**C**) and IL1β (**D**) and quantification of CD45+ and IL1β+ cells (right) in the distal colon sections from four group of mice. n=4 mice for each group. Scale bar: 100μm. Data are presented as mean ± SD. The data were analyzed by one-way ANOVA with Tukey's multiple comparisons test (B, C). *p < 0.05, **p < 0.01, ***p < 0.001.

8. Statistical analysis in the methods needs to be improved.

"462 All experiments were conducted independently at least three times. Each experiment contained at least two biological replicates... Unless otherwise specified, statistical significance was calculated using a two tailed unpaired student's t-test for two groups. The statistical analysis was performed with GraphPad Prism 9.0 software, and p-values were showed as *p < 0.05, **p < 0.01, ***p < 0.001, n.s, no significance."

The groups should be compared by one or two way Anova not T-tests. Exact details and

p-values should be given in a supplementary table.

Response: Thank you for your suggestion. We have revised our analytical methods and implemented the recommended statistical corrections. The complete set of updated results, including all supporting details and precise *p*-values, are now presented in **Dataset EV6**.

9. In many figures the time of sample collection is not indicated.

Response: Sorry about it. We have now documented the exact collection time for all biological samples in our revised figure legends.

10. In the paper the vil-cre conditional knock out is labeled "Bmal1 KO". This should be changed since this wording is typically used in the field for the Bmal1 knock out, not a conditional knock out. This should be changed to something like "Bmal1 cKO" to match the literature in the field.

Response: Following your suggestion, we have systematically revised all instances of "*Bmal1* KO" to "*Bmal1* cKO" in the manuscript.

Referee #2:

In their study "Genetic disruption of the circadian gene Bmal1 in the intestinal epithelium alleviates colonic inflammation" Hue et al. are exploring the role of the circadian clock gene Bmal1 in regulation of DSS induced colitis. The study demonstrates a circadian rhythm regulated susceptibility to DSS induced colitis that appears to be regulated by Bmal1. The study presents interesting findings in the context of colitis, but a number of points need to be addressed for this manuscript to be suitable for publication.
Response: Thank you for your insightful comments. As addressed below, we have addressed all your concerns by adding additional data, optimizing our statistical analysis, increasing the figure size, etc. We hope these revisions adequately address your concerns.

1. On a general level the, the statistical analyses need to be revised. The authors are using repeated t-tests at several places in the manuscript when they should be using a one-way ANOVA. The authors have not included any information regarding how they have assessed the cycling pattern of genes of interest. During image analysis, the authors have not calculated an average for each individual and it appears that they present each evaluated tissue section, which in my opinion is not the correct representation of that kind of data. Following revision of the statistical analysis some of the results may change, and the authors then might have to change some of their claims and interpretations throughout the paper.
Response: We sincerely appreciate the valuable comments. We have changed some of our statistical analysis methods in the following point-by-point response. In fact, for statistical analysis of images, our previous approach involved counting multiple identical areas on paraffin sections from multiple mice for comprehensive analysis. In the revised manuscript, we have adjusted our statistical methods so that each data point represents an independent animal in relevant results. We also assessed the rhythmicity of the circadian gene cycle expression pattern in control and *Bmal1* cKO mice utilizing the JTK cycle through the MetaCycle R package (**Fig. EV1A and 5A, B, D, G**).

2. Furthermore, the methods description needs to be expanded to explain the experiments in more detail. For example, there is no information regarding how colonic epithelium was isolated.
Response: Thank you very much for your careful reading. We apologize for our lack of clarity. We explain the method in more detail in the following point-by-point responses and manuscript. We would like to clarify that the samples analyzed through RNA-seq, immunoblotting, and RT-qPCR were specifically isolated from the intestinal crypts rather than the pure intestinal epithelial cells. To increase accuracy, we have changed some of the “epithelium” we mentioned in the text to “crypts”.

Below you can find a point-by-point description of my specific comments.

3. Abstract and introduction: Change dextrose to dextran.
Response: We apologize for this. In the revised manuscript, we have systematically

corrected all instances of "dextrose" to **"dextran"** throughout the text **(page 2, line 28; page 5, line 96)**.

4. Results and Figures:
Page 8: The authors need to run tests to validate that the genes they are evaluating in Fig. S1 are indeed oscillating not just making a statement that they are oscillating. They also need to support their claim of loss of oscillation in Bmal1 KO mice by statistical comparisons between groups.
Response: Thank you for your comments. We have implemented the recommended approach to label rhythmic gene expression in both control and *Bmal*1 cKO mice. Specifically, we applied JTK_Cycle analysis for circadian rhythmic detection, annotated rhythmic (p < 0.05) vs. non-rhythmic genes in Fig. S1. We have described circadian analysis in the Methods section **(page 24, lines 492-495)**.

5. Figure 1:
Fig. 1E: What comparison does the statistics refer to? I do not agree with the use of repeated t-test for data that is linked in time. The correct test to use is a two-way ANOVA. This comment also applies to 1B and C.
Fig. 1F: Please show the dots in the bar graph as you do in Fig. 1D.
Fig. 1F and 1G: The pictures are too small. Please increase the size so that it is possible to see the structure of the tissue and the immune cells. I do not agree with the representation of the data in 1G. One individual animal should be represented by one data point, not one data point per evaluated section. This type of analysis will overestimate the difference between groups. Please use the same type of bar graph as you use in 1D. Since you are comparing more than 2 groups you should use a one-way ANOVA or a non-parametric test, not multiple t-tests.
Response: Thanks for your suggestions. In the revised manuscript, we have updated the statistical analyses as recommended: Fig. 1B, 1C and 1E are now analyzed with two-way ANOVA, while Fig. 1F and 1G have been analyzed using one-way ANOVA. Additionally, we have increased the size of the pictures in Fig. 1F and 1G for clarity, and each data point represents an individual animal in the quantification.

6. Figure 2:
Fig. 2B and C: A two-way ANOVA would be more appropriate than repeated t-tests.
Fig. 2D and F: Please show the dots in the bar graph as you do in 2E.
Fig. 2G: Same comment as for Fig. 1. There should be one dot per individual not one for each analyzed section and the pictures are too small. Please increase the size of the pictures,
Response: We have revised the statistical analysis in Fig. 2B and 2C to two-ANOVA. Additionally, we have improved the visualization of Fig. 2D and 2F by showing individual data points as dots within the bar graphs, and increased the size of the images in Fig. 2G.

7. Figure 3:
Fig. 3D: please show the individual dots.
Fig. 3E and F: The pictures are too small and change the data so that there is one dot per

individual mouse.

Response: we have shown the individual data dots in Fig.3D and increased the size of the images. In Fig.3E and 3F, each dot represents an individual animal.

8. Figure 4:

Fig. 4A: Pictures are too small and redo the analysis as suggested for the other images.

Fig. 4B: Show the individual data points.

What do the authors mean by bubble formation? What does % spheres refer to? To me it looks like the control spheroids are more differentiated as compared to the Bmal1 KO spheroids, as the epithelium is thicker in the control.

The authors need to add a control where they show the effect of 4-OHT on Cre- Bmal1fl/fl mice to confirm that the observed effect is related to loss of Bmal1 and not due to the 4-OHT treatment.

Fig. 4H: Same comment her as for the other analyzed images. One point per individual.

Response: We have carefully revised the figures to maximize their size while maintaining clarity, and each data point represents an individual animal in Fig. 4B and 4H.

Typically, control organoids exhibit a budding phenotype, whereas the *Bmal1* cKO organoids primarily display a vacuolar morphology. The sphere organoids were increased in the *Bmal1* cKO organoids.

We induced the knockout of *Bmal1* in organoids by adding 4-OHT for 24 hours. After passage on the third day, we observed and analyzed the relevant phenotypes on the sixth day **(Response Figure 8A, new Fig.4D)**. The toxicity of 4-OHT itself has little impact on organoids. We also used organoids from *Bmal1*$^{fl/fl}$ mice treated with the same amount of 4-OHT to avoid the toxicity of tamoxifen. We found that only organoids derived from *Villin-CreERT2; Bmal1*$^{fl/fl}$ mice showed a blebbing phenotype after 4-OHT treatment **(Response Figure 8A, new Fig.4E)**. Furthermore, we have previously assessed the possible toxicity of tamoxifen on mice and showed that it has no apparent effects in the dose range we used in the experiments (four consecutive daily doses of 20 mg/mL tamoxifen)(Liu *et al*, 2023).

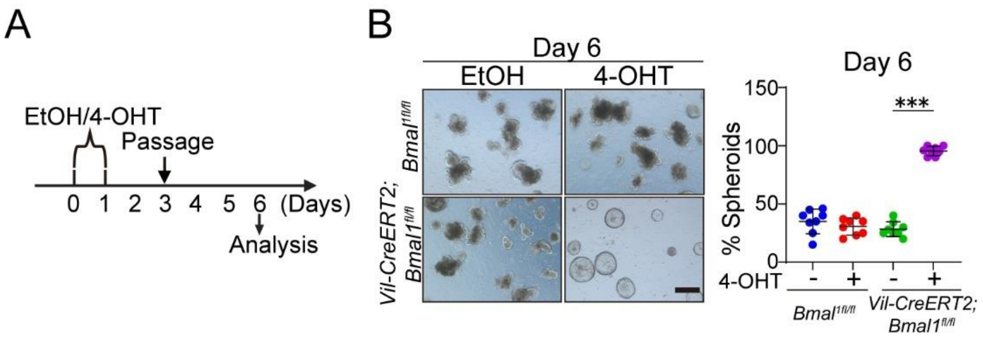

Response Figure 8 ***Bmal*1 cKO organoids exhibit bubble morphology not caused by 4-OHT.** (**A**) Schematic showing knockout of *Bmal1* gene in colonic organoids by adding 4-OHT for 24h, organoids were passaged on day 3 and collected or photographed on day 6 for subsequent analysis. (**B**) Organoids derived from the crypts of *Bmal1*$^{fl/fl}$ and *Villin-CreERT2; Bmal1*$^{fl/fl}$ mice were treated with EtOH or 4-OHT for 24 h. Images were

taken on day 6, and the bubble formation rate (right) were counted from 8 fields of view from three independent experiments. Scale bar: 400μm. Data are presented as mean ± SD. The data were analyzed by one-way ANOVA with Tukey's multiple comparisons test (**B**) ***p < 0.001.

9. Figure 5:

Fig. 5E and F: The pictures are too small to see the apoptotic cells. Please change the graph so that one point represents one individual.

Have the authors measured villus and crypt length in the Bmal1 KO mice? If apoptosis is reduced one would expect the villi and/or crypt length to be affected.

Response: We have increased the size of the pictures while maintaining clarity and ensuring that each data point explicitly represents an individual animal in Fig .5E and 5F.

We have measured crypt and villus lengths and observed an increase in villus length in the proximal small intestine and crypt length in the distal colon of *Bmal1* cKO mice **(Response Figure 9, new Fig. EV2B)**. We propose that these morphological changes may be due to the reduced apoptosis.

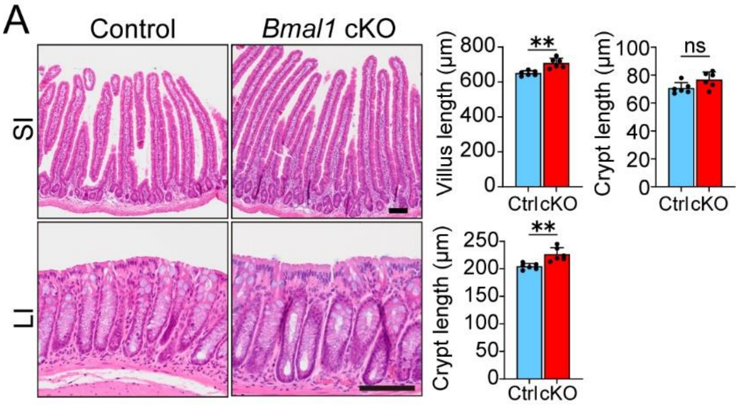

Response Figure 9 ***Bmal1* deficiency increased villus length in the proximal small intestine and enhanced crypt length in the distal colon.** Histological images and quantification of villus length (top, left) and crypt length of the proximal small intestine (top, right) and distal large intestine (bottom) from control and *Bmal1* cKO mice. n=6 mice for each group. Scale bar: 100μm. Data are presented as mean ± SD. The data were analyzed by two- tailed Student's t-test. **p < 0.01, n.s, no significance.

10. Figure 6:

Fig. 6E and F: The pictures are too small to see the stained cells. Same comment as previously, change the graph so that one individual is represented by one point.

I think it would help the readers of this manuscript with a schematic drawing of how these experiments were done. I first thought that mice were given DSS by gavage at the different time points in the presence of SR, and not that SR given at different time points to mice given DSS in the drinking water.

Response: We have increased the size of the pictures, changed the representation of the data to one point per animal and showed the individual values in the bar graphs. We have placed the experimental design scheme of DSS and SR9009 treatments **(new Fig. 6C)**.

11. Figure S1:

The authors need to evaluate the data in Fig. 1SA for oscillating patterns not just state that they are oscillating and that oscillations are being lost in the Bmal1 KO mice. https://academic.oup.com/bioinformatics/article/32/21/3351/2415176, https://academic.oup.com/bioinformatics/article/36/3/773/5544107

Response: In response to your comments, we have implemented the recommended approach to label rhythmic gene expression in both control and *Bmal*1 cKO mice. Specifically, we applied JTK_Cycle analysis for circadian rhythmicity detection, annotated rhythmic ($p < 0.05$) vs. non-rhythmic genes in relevant figures. Circadian analysis methods we have described in the Methods **(page 24, lines 492-495)**.

12. Figure S2:

The pictures are too small and the statistical representation of the data needs to be changed to one point per animal.

Response: We have increased the size of the pictures and changed the representation of the data to one point per animal.

13. Figure S3:

The pictures are too small in C and D. Please change the data in C to one point per individual. Is it correct that the scale bar in D is 200 µm? That would make the crypts approx. 600 µm long.

Fig. S3F: It is not clear to me what this figure represents.

Response: We have increased the panel sizes of Figures C and D, corrected the scale bar in Figure S3D from 200 µm to 100 µm, and revised the data representation in Figure C to display individual data points from a single biological replicate.

Fig. S3F is a Venn diagram illustrating the overlap between two datasets: (1) genes identified by RNA-seq as downregulated in *Bmal1* cKO mice under DSS-induced colitis conditions, and (2) genes identified by CUT&Tag-seq as BMAL1-binding targets in wild-type colon organoids. The overlapping region represents genes that are both bound by BMAL1 and transcriptionally downregulated upon *Bmal1* cKO during colitis. The overlapping genes were used for subsequent functional analyses to investigate BMAL1's role in colitis pathogenesis.

14. Figure S4:

Please show the individual values in the bar graphs and write out the number of individual experiments.

Response: We have increased the size of the pictures, changed the representation of the data to one point per animal, showed the individual values in the bar graphs and wrote out the number of individual experiments in relevant figure legends.

15. Figure S5:

Fig. S5C and D: Please increase the size of the pictures and change the data into one point per individual.

Response: We have increased the size of the pictures, changed the representation of the data

to one point per animal.

16. Figure S6:
The figure legend does not match the figure. In B, use a one-way ANOVA instead of repeated t-test.
Response: We have corrected the figure legend. In B, we have analyzed the data with one-way ANOVA.

Materials and methods:

17. Human UC samples:
Please include information regarding the human samples. Age, gender, disease activity and treatment status. How were the human tissues stained? Did you use the same method and antibody that was used for the mouse tissue?
Response: We have added the relevant clinical information for the patients in **Dataset EV4**. We have also detailed the human tissue IHC staining protocol in the Methods section **(page 21, lines 423-453)**, confirming that the same antibodies and staining methods were applied to both mouse and human samples.

DSS colitis:
Line 363. If you have paraffin embedded tissues, why did you freeze the tissues?
Response: Sorry about the negligence. Here we used fresh tissues rather than frozen tissues. The mistake is corrected now.

18. Intestinal crypt isolation and organoid culture:
For how long were the organoids cultured prior to analysis? How was the organoid diameter analyzed? Please expand the description of how organoids were grown for the experiments and how they were analyzed.
Response: We have now provided additional details in the Methods section **(page 20, lines 400-401)**. Specifically, to exclude the effect of non-epithelial cells, organoids were cultured and passaged for 2-3 generations before experiments. To activate Cre recombinase in organoids, 4-OHT was added to the culture medium for 24h, and organoids were then passaged on day 3 and observed or harvested on day 6 for subsequent analyses. To assess the organoid growth, organoids were digested with TrypLE into single cell culture for organoids diameter statistic and immunoblotting **(page 21, lines 404-406)**. The diameter was measured by ImageJ.

19. RNA isolation and qPCR:
How were the colonic crypts isolated? Purification by TRIZOL needs more details. Table S1 shows one housekeeping gene for control and one for colitis. Does that mean that the control and DSS data were normalized against different genes? If that was the case, explain why and how that is the correct way of doing this kind of analysis.
Response: Isolation of the colonic crypts was described in the Methods section. Total RNA was extracted and purified from colonic crypts using TRIZOL reagent (Thermo Fisher Scientific) according to the manufacturer's instructions **(page 21, lines 407-408)**. In our

original analysis, we used both GAPDH and Actin as housekeeping genes to normalize the expression of target genes. We found that normalization with either of the housekeeping genes yielded consistent results. To avoid ambiguity, we have re-ran the qPCR experiments and used the GAPDH for all qPCR experiments now.

20. Immunoblotting: How was the colonic epithelium isolated?
Response: The samples for RNA-seq, immunoblotting, and RT-qPCR analyses were isolated from the intestinal crypts rather than the intestinal epithelium. To increase accuracy, we have indicated “crypts” where it is applied in the text.

21. H&E, Alcian blue, IF and IHC:
Please provide information regarding the kit that was used for H&E staining and Alcian blue. The authors have used mouse anti Il1b, IFNg and F4/80 for the analysis. Since these proteins are produced by immune cells and the authors are quantifying the number of lamina propria immune cells they cells in the lamina propria they have to provide information regarding how they have separated the real signal from the signal from the anti-mouse secondary antibody binding to lamina propria B cells.
Response: As mentioned in the Methods, H&E staining and Alcian Blue staining were performed using a kit (Sangon Biotech). Relevant information has been provided in the reagent table.

We acknowledge the reviewer’s concern regarding the potential for non-specific binding of anti-mouse secondary antibodies to endogenous immunoglobulins in the lamina propria. To address this issue, we conducted immunofluorescence staining only using the secondary anti-mouse antibody under the same exposure conditions. As shown in **Response Figure 10**, no background signals were detected, confirming that the secondary antibody alone does not produce non-specific binding. When the primary anti-mouse antibody was included, a strong and specific signal was observed, further validating the specificity of our staining. The results demonstrate that the observed signals are specific to the target proteins (Il1β, IFNγ, and F4/80) and not due to non-specific binding of the secondary antibody.

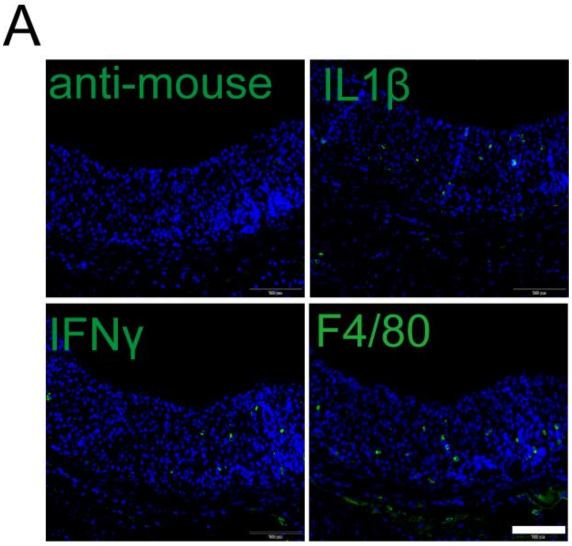

Response figure 10 **Mouse primary antibody is highly specific.** Serial paraffin sections

from the same mouse were stained with a single anti-mouse secondary antibody (the upper left) and other indicated mouse primary antibodies. All the signals were at the same exposure time.

22. PI staining of organoids: Was there no washing steps added to the staining protocol?
Response: We have added a detailed experimental procedure for PI staining **(page 23, lines 454-457)**.

Referee #3:

The disruption of circadian clocks has been linked to increased incidence of a range of human disorders. In this study, Hua et al. aims to investigate the functional relevance of the intestinal clock intestinal inflammation. They generated an intestinal epithelium-specific Bmal1 KO mice and examined severity of DSS-induced colitis (model for inflammatory bowel disease) in this animal. They observed that WT control mice in the active phase (ZT12) were resistant to DSS-induced colitis when compared to animals in the resting phase (ZT0). Furthermore, Bmal1 KO rendered intestinal epithelium more tolerant to DSS-induced acute colitis, as evidenced by less weight loss, more intact epithelial barrier structure and function, as well as less infiltration of inflammatory factors and immune cells compared to control mice. They then performed RNA-seq and immunostaining to provide support that this is due to the pro-apoptotic function of BMAL1. When BMAL1 level is low (ZT12) or when it is deleted (in the BMAL1 KO), this reduced cell apoptosis, leading to the resistant phenotype. Finally, they determined the therapeutic efficacy of the REV-ERBa agonist SR9009 against DSS-induced colitis, and found that it is most effective at DSS-induced colitis at ZT0 (although it is also effective at ZT12). They conclude that targeting BMAL1 function may be a potential approach to treat inflammation-related GI disorders.

Major comments:

1. Figure 6: I understand that SR9009 has previously been shown to be an agonist for REV-ERBa. Nonetheless, they should still show BMAL1 and cleaved-caspase3 level in their SR9009 treatment samples to support that the therapeutic outcomes they observed are mediated by reduced BMAL1 and apoptosis as they predicted. This is especially important given the prior published contradictory results based on global Bmal1 KO and the fact that they also saw an effect for their ZT12+SR sample (in addition to their ZT0+SR sample).
Response: Thank you for the insightful comments. To address your comments, we investigated the effects of SR9009 in vitro by treating organoids with the compound and observed a significant decrease of *Bmal1* expression **(new Fig. 6A)**. Cleaved-caspase 3 staining revealed that both SR9009-treated and *Bmal1* cKO organoids exhibited reduced apoptosis compared to control organoids **(new Fig. 6B)**. Furthermore, decreased BMAL1 and cleaved-caspase3 levels were detected in SR9009 treated mice at ZT0 **(new Fig. 6J)**. These findings demonstrate that SR9009 alleviates intestinal inflammation through suppressing of *Bmal1* expression and inhibition of apoptosis.

We have optimized the experiment design **(new Fig. 6C)**. In our latest experimental design, wild-type mice were given DSS solution ad libitum for five days at ZT0, ZT8, and ZT12, respectively, and switched to water at ZT0, ZT8, and ZT12 on the fifth day. To ensure consistent treatment duration in all the experimental groups, SR9009 administration was initiated on day 3 through intraperitoneal injections at ZT0, ZT8, and ZT12, maintaining this regimen for five consecutive days in DSS-treated mice. Tissues were collected subsequently at ZT0, ZT8, and ZT12 on day 8, thereby standardizing both the SR9009 treatment period and circadian sampling points. We think that this modified

experimental design is more scientifically rigorous, as it controls both treatment duration and circadian time variables, thereby minimizing potential confounding factors and enhancing the reliability of experimental outcomes.

Mice treated with SR9009 at ZT0 exhibited superior therapeutic outcomes, evidenced by less body weight loss, lower disease activity index, higher survival rates and longer colon lengths, compared to those treated at other time points, with ZT12 being the least effective **(new Fig. 6D-G, new Fig. EV5A)**. Histochemical staining showed that SR9009 treatment at ZT0 had the lowest histological scores **(new Fig. 6H)**. Furthermore, fewer apoptotic cells and decreased BMAL1 and cleaved-caspase3 levels were detected in SR9009 treated mice at ZT0 **(new Fig. 6I)**. Consistently, SR9009 treatment at ZT0 significantly reduced infiltration of immune $CD45^{+}$ cells and lower expression levels of inflammatory protein IL-1β **(Fig. EV5B, C)**. Collectively, these results support our conclusion that SR9009 treatment at ZT0 has the best beneficial efficacy in DSS-induced colitis.

2. Figure 5 F: The authors should provide data for ZT0 and ZT12 for both genotypes (Ctrl and KO) to highlight the loss of the rhythmicity in the KO. In addition, it will be less confusing they should use different color schemes to differentiate timepoints (ZT0 and ZT12) and genotypes (Control and KO).

Response: Thank you for your advice. We have collected the colonic crypts of wild-type mice for more time (ZT0, ZT6, ZT12, ZT18) for RNA sequencing and found that the apoptotic pathway showed rhythmicity. Meanwhile, cleaved-caspase 3 staining further demonstrated that this apoptotic rhythmicity in the intestinal epithelium was *Bmal1*-dependent (Fig. 5G).

3. The authors should provide quantification and statistics for all western blots and immunostaining. Only some are provided.

Response: We have provided quantifications and statistics for all immunoblots and immunostaining.

Minor comments:

1. I would suggest changing the first sentence in the abstract. It is awkward to suggest the clock regulates a disease. Perhaps just it to "Disruption of the circadian clock increases the risk (or incidence) of IBD.

Response: We sincerely appreciate your suggestions. Although some studies have shown that disruption of the circadian clock increased the risk (or incidence) of IBD, here we revealed a novel function of the circadian gene *Bmal1*. Inducible knockdown of *Bmal1* in mouse intestinal epithelium effectively alleviated DSS induced colitis, suggesting a more complex function of the biological clock in the regulation of IBD. We think it might be more appropriate to change ‘the circadian clock regulates disease’ to “Disruption of the circadian clock has been shown to associate with the development of inflammatory bowel disease (IBD)”.

2. Line 37: "injure" should be "injury".

Response: Sorry about it. We have corrected the "injure" into "injury".

3. Define abbreviations the first time you use it in text of figure. For example, line 137, H&E. In figure legends, define SI and LI (small intestine and large intestine).

Response: Thank you for pointing it out. We have spelled out the full names for the abbreviations.

4. Line 160: define what you mean by "better structure", or include citations that define better structure.

Response: We have modified the "better structure" to "more intact structure".

5. Line 189: Please elaborate why only the overlapping 495 genes are potential Bmal1-regulated genes? Why aren't all genes that were identified to be BMAL1-bound considered to be Bmal1-regulated genes?

Response: The statement is related to Fig. S3F, which is a Venn diagram illustrating the overlap between two datasets: (1) genes identified by RNA-seq as downregulated in *Bmal1* cKO mice under DSS-induced colitis conditions, and (2) genes identified by CUT&Tag-seq as BMAL1-binding targets in wild-type colon organoids. A total of 3,002 genes were identified as potential BMAL1 targets, but these genes may either be directly regulated by BMAL1 or simply bound by BMAL1 without transcriptional consequences. To refine the BMA1 targets, we overlapped these BMAL1-binding genes with the downregulated transcriptome (3110 genes) in *Bmal1* cKO mice under DSS-induced colitis conditions. This intersection allowed us to generate a list of BMAL1-regulated genes (495 genes), which were subsequently used for functional enrichment and pathway analyses to elucidate the potential mechanisms by which BMAL1 modulates colitis.

6. Line 205: indicate dead cells are stained by propidium iodide.

Response: Thank you for your advice. We have made the indication. **(page 11, lines 211-212)**.

References

Avila Cobos F, Alquicira-Hernandez J, Powell JE, Mestdagh P, De Preter K (2020) Benchmarking of cell type deconvolution pipelines for transcriptomics data. *Nat Commun* 11: 5650

De Los Santos H, Collins EJ, Mann C, Sagan AW, Jankowski MS, Bennett KP, Hurley JM (2020) ECHO: an application for detection and analysis of oscillators identifies metabolic regulation on genome-wide circadian output. *Bioinformatics* 36: 773-781

Hao KL, Zhai QC, Gu Y, Chen YQ, Wang YN, Liu R, Yan SP, Wang Y, Shi YF, Lei W *et al* (2023) Disturbance of suprachiasmatic nucleus function improves cardiac repair after myocardial infarction by IGF2-mediated macrophage transition. *Acta Pharmacol Sin* 44: 1612-1624

Lefta M, Campbell KS, Feng HZ, Jin JP, Esser KA (2012) Development of dilated cardiomyopathy in Bmal1-deficient mice. *Am J Physiol Heart Circ Physiol* 303: H475-485

Liu Y, Huang M, Wang X, Liu Z, Li S, Chen YG (2023) Segregation of the stemness program from the proliferation program in intestinal stem cells. *Stem Cell Reports* 18: 1196-1210

Paschos GK, Ibrahim S, Song WL, Kunieda T, Grant G, Reyes TM, Bradfield CA, Vaughan CH, Eiden M, Masoodi M *et al* (2012) Obesity in mice with adipocyte-specific deletion of clock component Arntl. *Nat Med* 18: 1768-1777

Rosselot AE, Park M, Kim M, Matsu-Ura T, Wu G, Flores DE, Subramanian KR, Lee S, Sundaram N, Broda TR *et al* (2022) Ontogeny and function of the circadian clock in intestinal organoids. *EMBO J* 41: e106973

Tsoucas D, Dong R, Chen H, Zhu Q, Guo G, Yuan GC (2019) Accurate estimation of cell-type composition from gene expression data. *Nat Commun* 10: 2975

Wu G, Anafi RC, Hughes ME, Kornacker K, Hogenesch JB (2016) MetaCycle: an integrated R package to evaluate periodicity in large scale data. *Bioinformatics* 32: 3351-3353

Yu F, Wang Z, Zhang T, Chen X, Xu H, Wang F, Guo L, Chen M, Liu K, Wu B (2021) Deficiency of intestinal Bmal1 prevents obesity induced by high-fat feeding. *Nat Commun* 12: 5323

Zhang Z, Zhai Q, Gu Y, Zhang T, Huang Z, Liu Z, Liu Y, Xu Y (2020) Impaired function of the suprachiasmatic nucleus rescues the loss of body temperature homeostasis caused by time-restricted feeding. *Sci Bull (Beijing)* 65: 1268-1280

Dear Dr. Chen,

Thank you for the submission of your revised manuscript to our editorial offices. I have now received the reports from the three referees that were asked to re-evaluate the study, you will find below. As you will see, the referees now support its publication in EMBO reports. However, referees #1 and #3 have remaining concerns and suggestions to improve the manuscript, I ask you to address in a final revised manuscript. Please also provide a final p-b-p-response regarding these points.

Moreover, I have these editorial requests:

- Please provide the abstract written in present tense and with no more than 175 words.

- We now use CRediT to specify the contributions of each author in the journal submission system. CRediT replaces the author contribution section. Please use the free text box to provide more detailed descriptions and do NOT provide your final manuscript text file with an author contributions section. See also our guide to authors:
https://www.embopress.org/page/journal/14693178/authorguide#authorshipguidelines

- Please order the manuscript sections like this, using these names:
Title page - Abstract - Keywords - Introduction - Results - Discussion - Methods - Data availability section - Acknowledgements (including the funding information) - Disclosure and Competing Interests Statement - References - Figure legends - Expanded View Figure legends

Thus, please fuse the funding section with the Acknowledgements.

- The Data availability section (DAS) is restricted for information on primary datasets produced in the study (e.g. RNA-seq, ChIP-seq, structural and array data) that are deposited in an public database and deposited source data. Thus, please remove all further text not related to externally deposited datasets from this section (i.e. 'The authors declare that all data supporting the findings of this study are available upon reasonable request. All data needed to evaluate the conclusions are present in the paper and/or the Supplementary Materials.').

- Please remove the mention of GSE100339, GSE3365 and GSE75214 form the DAS. These datasets deposited by other authors need to be mentioned as data references as part of the reference list, together with a citation to the related paper. Please do that. See:
https://www.embopress.org/page/journal/14693178/authorguide#datacitation

- Please remove the legends for the datasets from the main manuscript text file. Please add each legend as the first TAB to the respective excel files.

- Please check again that the number "n" for how many independent experiments were performed, their nature (biological versus technical replicates), the bars and error bars (e.g. SEM, SD) and the test used to calculate p-values is indicated in the respective figure legends. Please also check that all the p-values are explained in the legend, and that these fit to those shown in the figure. Please provide statistical testing where applicable. Please avoid the phrase 'independent experiment', but clearly state if these were biological or technical replicates. Please also indicate (e.g. with n.s.) if testing was performed, but the differences are not significant. In case n=2, please show the data as separate datapoints without error bars and statistics. See also:
http://www.embopress.org/page/journal/14693178/authorguide#statisticalanalysis

If n<5, please show single datapoints for diagrams. Moreover:
- Please note that the exact p values are not provided in the legends of figures 1B, C, D, E, F, G; 2B, C, D, E, F, G; 3D-F; 4A, B, E, F, G, H, J; 5G, 6A, B, D, E, F, G, H, I; EV1 A, EV2 B, EV3 B, D; EV4 A-C; EV5 B, C; EV6 A-C.
- Please indicate the statistical test used for data analysis in the legends of figures 3A, B, H.
- Please note that in figures 1B, 3D-F there is a mismatch between the annotated p values in the figure legend and the annotated p values in the figure file that should be corrected.
- Please note that the box plots need to be defined in terms of minima, maxima, centre, bounds of box and whiskers, and percentile in the legend of figure EV6A
- Please note that information related to n is missing in the legends of figures EV6 A, B

- Please add to each legend (main, EV and Appendix figures, where applicable) a 'Data Information' section (or name the provided section like this) explaining the statistics used or providing information regarding replicates and scales. See:

https://www.embopress.org/page/journal/14693178/authorguide#figureformat

- We noted repeating background patters in Fig. EV2F (see attached file). What is this? Please explain in the final p-b-p-

response.

- Thank you for providing the source data. Please upload the SD as one folder per figure, grouping together all files for all panels (labeled as such) for one figure.

In addition, I would need from you uploaded separately:
- a short, two-sentence summary of the manuscript (not more than 35 words).
- two to four short (!) bullet points highlighting the key findings of your study (two lines each).
- a schematic summary figure as separate file that provides a sketch of the major findings (not a data image) in jpeg or tiff format (with the exact width of 550 pixels and a height of not more than 400 pixels) that can be used as a visual synopsis on our website.

I look forward to seeing the final revised version of your manuscript when it is ready. Please let me know if you have questions regarding the revision.

Best,

Achim Breiling
Senior Editor
EMBO Reports

Referee #1:

The authors have addressed most of my questions and concerns.

There are two issues that were not addressed:

1. Question #4 about the RNA-seq data comparison with other studies was ignored. This needs to be considered before publication. The use of a Vil-cre cKO should be very similar to the authors Vil-creERT cKO, but the authors simply say it is irrelevant due to developmental effects, without performing any analysis or scientific basis to support this statement. Of note, the authors analysis of the cells tested in response table 1 indicates the cKO is completely depleted of stem cells.

2. Question #5 response raised a concern. The authors state that the paper (Rosselot et al, 2002, PMID 34704277) found that organoids have no rhythmic transcriptome. This is completely incorrect - the paper found thousands of genes that are rhythmic in organoids, and other papers in the field have used these as models of the clock as well. This needs to be corrected.

I support publication with these changes addressed.

Referee #2:

The authors have addressed all of my comments and I find the manuscript suitable for publication.

Referee #3:

The authors have adequately addressed my comments. I very much appreciate the addition of new data and data analysis to further support their conclusions.

They should make one minor edit: Figure 6C: "Sacrifice" is mis-spelled.

Thank you for the submission of your revised manuscript to our editorial offices. I have now received the reports from the three referees that were asked to re-evaluate the study, you will find below. As you will see, the referees now support its publication in EMBO reports. However, referees #1 and #3 have remaining concerns and suggestions to improve the manuscript, I ask you to address in a final revised manuscript. Please also provide a final p-b-p-response regarding these points.

Moreover, I have these editorial requests:

- Please provide the abstract written in present tense and with no more than 175 words.
Response: We have changed the tense of the abstract section to present tense and with no more than 175 words.

- We now use CRediT to specify the contributions of each author in the journal submission system. CRediT replaces the author contribution section. Please use the free text box to provide more detailed descriptions and do NOT provide your final manuscript text file with an author contributions section. See also our guide to authors: https://www.embopress.org/page/journal/14693178/authorguide#authorshipguidelines
Response: We have removed the detailed descriptions in our final manuscript and used CrediT to specify the contributions of each author.

- Please order the manuscript sections like this, using these names:
Title page - Abstract - Keywords - Introduction - Results - Discussion - Methods - Data availability section - Acknowledgements (including the funding information) - Disclosure and Competing Interests Statement - References - Figure legends - Expanded View Figure legends
Response: We have sorted the manuscripts according to the relevant requirements

Thus, please fuse the funding section with the Acknowledgements.
Response: We have ordered the manuscripts according to the relevant requirements and fused the funding section with the acknowledgements.

- The Data availability section (DAS) is restricted for information on primary datasets produced in the study (e.g. RNA-seq, ChIP-seq, structural and array data) that are deposited in an public database and deposited source data. Thus, please remove all further text not related to externally deposited datasets from this section (i.e. 'The authors declare that all data supporting the findings of this study are available upon reasonable request. All data needed to evaluate the conclusions are present in the paper and/or the Supplementary Materials.').

- Please remove the mention of GSE100339, GSE3365 and GSE75214 form the DAS. These datasets deposited by other authors need to be mentioned as data references as part of the reference list, together with a citation to the related paper. Please do that.

See:
https://www.embopress.org/page/journal/14693178/authorguide#datacitation
Response: We have removed further text from the Data Availability section and cited the public database and articles we used according to relevant requirements.

- Please remove the legends for the datasets from the main manuscript text file. Please add each legend as the first TAB to the respective excel files.
Response: We have made changes as required.

- Please check again that the number "n" for how many independent experiments were performed, their nature (biological versus technical replicates), the bars and error bars (e.g. SEM, SD) and the test used to calculate p-values is indicated in the respective figure legends. Please also check that all the p-values are explained in the legend, and that these fit to those shown in the figure. Please provide statistical testing where applicable. Please avoid the phrase 'independent experiment', but clearly state if these were biological or technical replicates. Please also indicate (e.g. with n.s.) if testing was performed, but the differences are not significant. In case n=2, please show the data as separate datapoints without error bars and statistics. See also:
http://www.embopress.org/page/journal/14693178/authorguide#statisticalanalysis

If n<5, please show single datapoints for diagrams. Moreover:
- Please note that the exact p values are not provided in the legends of figures 1B, C, D, E, F, G; 2B, C, D, E, F, G; 3D-F; 4A, B, E, F, G, H, J; 5G, 6A, B, D, E, F, G, H, I; EV1 A, EV2 B, EV3 B, D; EV4 A-C; EV5 B, C; EV6 A-C.
- Please indicate the statistical test used for data analysis in the legends of figures 3A, B, H.
- Please note that in figures 1B, 3D-F there is a mismatch between the annotated p values in the figure legend and the annotated p values in the figure file that should be corrected.
- Please note that the box plots need to be defined in terms of minima, maxima, centre, bounds of box and whiskers, and percentile in the legend of figure EV6A
- Please note that information related to n is missing in the legends of figures EV6 A, B

- Please add to each legend (main, EV and Appendix figures, where applicable) a 'Data Information' section (or name the provided section like this) explaining the statistics used or providing information regarding replicates and scales. See:

https://www.embopress.org/page/journal/14693178/authorguide#figureformat
Response: Thank you for your insightful suggestion. We have carefully checked the numbers “n” and p values for all our data. The exact p-values are displayed in the figures and the number “n” are stated in the figure legends. All statistical methods are explained in the relevant figure legends.

- We noted repeating background patters in Fig. EV2F (see attached file). What is this? Please explain in the final p-b-p-response.

Response: We are very sorry that we did not understand what you meant. We have carefully checked our source data. The pictures are from different mice. We have uploaded the source data in the system for reference.

- Thank you for providing the source data. Please upload the SD as one folder per figure, grouping together all files for all panels (labeled as such) for one figure.

Response: We have made changes as required.

In addition, I would need from you uploaded separately:
- a short, two-sentence summary of the manuscript (not more than 35 words).
- two to four short (!) bullet points highlighting the key findings of your study (two lines each).
- a schematic summary figure as separate file that provides a sketch of the major findings (not a data image) in jpeg or tiff format (with the exact width of 550 pixels and a height of not more than 400 pixels) that can be used as a visual synopsis on our website.

Response: We appreciate your insightful comments.We have uploaded a short summary, 2-4 highlights and a schematic summary figure as required.

I look forward to seeing the final revised version of your manuscript when it is ready. Please let me know if you have questions regarding the revision.

Best,

Achim Breiling
Senior Editor
EMBO Reports

Referee #1:

The authors have addressed most of my questions and concerns.

There are two issues that were not addressed:

1. Question #4 about the RNA-seq data comparison with other studies was ignored. This needs to be considered before publication. The use of a Vil-cre cKO should be very similar to the authors Vil-creERT cKO, but the authors simply say it is irrelevant

due to developmental effects, without performing any analysis or scientific basis to support this statement. Of note, the authors analysis of the cells tested in response table 1 indicates the cKO is completely depleted of stem cells.

Response: We have reviewed the two recommended studies (PMID: 36241650 and 38918576). PMID: 36241650 provides microbiota sequencing data and metabolite data but does not provide relevant transcriptomic data. Unfortunately, our analysis of the transcriptome data from PMID 38918576 did not reveal oscillatory expression patterns similar to those observed in our study. In this study, mice were sacrificed in the second day of darkness at the indicated circadian time to obtain colonic tissues for RNA-seq analysis. It contains not only crypts but also stromal layers and mesenchymal cells. In contrast, we housed mice under a 12 h light/12 h dark cycle and colonic crypts were harvested at the indicated circadian times. We also analyzed public transcriptome data (Wang *et al*, 2017) from the small intestinal epithelium of wild-type mice housed under a 12 h light/12 h dark cycle, which also revealed that the expression of a portion of genes, especially apoptosis-related genes, exhibited a rhythmic pattern **(Fig. 5D-F)**. We speculate that this dissimilarity may result from variations in cellular composition and differences in light-dark sampling conditions.

Several studies have shown that Cre mice and CreER mice may have different phenotypes (Liu *et al*, 2023; Qi *et al*, 2017). The phenotypic differences may be due to the difference in the timing of knockout, such as the difference between the developmental stage and the adult stage. It has been reported that deletion of *Bmal1* in Villin-Cre mice during the embryonic stage affects normal intestinal functions, such as rhythmicity of microbiota disruption, immune cell recruitment (Heddes et al, 2022). and is more susceptible to DSS-induced colitis (Jochum et al, 2023; Niu et al, 2024). However, we used CreER mice to exclude these potential effects of Cre constitutive knockout mice. Inactivation of *Bmal1* in the intestinal epithelium of adult mice had no significant effect on intestinal homeostasis but was more tolerant to DSS. These results indicate that the continuous loss of *Bmal1* during intestinal development and conditional loss under adult homeostasis do have different effects. We have discussed this issue in the discussion section **(page 16, lines 301-308)**.

We performed the DWLS deconvolution (Avila Cobos *et al*, 2020; Tsoucas *et al*, 2019) to quantify cell proportions in our RNA-seq data. We found that in the samples isolated from control mice, the proportions of six epithelial cell types (EEC, Enterocyte, Goblet_cell, Stem_cell, TA_cell, and Tuft_cell) reached 80.86% and 93.17%, respectively, while in the cKO samples, the proportions were both around 99% of epithelial cells (see the following **Response table 1)** . We found that cKO mice exhibit a higher proportion of stem cells (around 15%) compared to control mice (around 0%), suggesting reduced tissue damage.

Cell ratio	Control 1#	Control 2#	cKO 1#	cKO 2#
EEC	3.84%	2.97%	0.50%	0.46%
Enterocyte	28.23%	37.13%	47.04%	41.55%
Goblet_cell	12.64%	19.38%	9.68%	14.14%
Stem_cell	0.00%	0.00%	15.56%	18.66%
TA_cell	35.19%	32.69%	27.14%	24.42%
Tuft_cell	0.96%	1.00%	0.07%	0.14%
**Total**	80.86%	93.17%	99.99%	99.37%
B_cell	6.29%	0.00%	0.01%	0.63%
Macrophage	4.86%	6.26%	0.00%	0.00%
myoFibroblast	0.23%	0.00%	0.00%	0.00%

Response table 1. **DWLS deconvolution to quantify cell proportions in control and *Bmal1* cKO mice at day 7 following DSS treatment**

2. Question #5 response raised a concern. The authors state that the paper (Rosselot et al, 2002, PMID 34704277) found that organoids have no rhythmic transcriptome. This is completely incorrect - the paper found thousands of genes that are rhythmic in organoids, and other papers in the field have used these as models of the clock as well. This needs to be corrected.

Response: We apologize for this mistake. In this study, the authors demonstrate that differentiation of human induced pluripotent stem cells into multicellular human intestinal organoids exhibit transient circadian rhythms but fail to sustain long-term rhythmicity. In contrast, approximately 3-10% of transcripts display circadian oscillations in intestinal organoids isolated from mice and human tissues. We have corrected it in the text **(page 16, lines 314-315)**.

I support publication with these changes addressed.

Referee #2:

The authors have addressed all of my comments and I find the manuscript suitable for publication.

Referee #3:

The authors have adequately addressed my comments. I very much appreciate the addition of new data and data analysis to further support their conclusions.

They should make one minor edit: Figure 6C: "Sacrifice" is mis-spelled.
Response: We appreciate your insightful comments. We have corrected it.

References

Avila Cobos F, Alquicira-Hernandez J, Powell JE, Mestdagh P, De Preter K (2020) Benchmarking of cell type deconvolution pipelines for transcriptomics data. *Nat Commun* 11: 5650

Heddes M, Altaha B, Niu Y, Reitmeier S, Kleigrewe K, Haller D, Kiessling S (2022) The intestinal clock drives the microbiome to maintain gastrointestinal homeostasis. *Nat Commun* 13: 6068

Jochum SB, Engen PA, Shaikh M, Naqib A, Wilber S, Raeisi S, Zhang L, Song S, Sanzo G, Chouhan V *et al* (2023) Colonic Epithelial Circadian Disruption Worsens Dextran Sulfate Sodium-Induced Colitis. *Inflamm Bowel Dis* 29: 444-457

Liu L, Wang Y, Yu S, Liu H, Li Y, Hua S, Chen YG (2023) Transforming Growth Factor Beta Promotes Inflammation and Tumorigenesis in Smad4-Deficient Intestinal Epithelium in a YAP-Dependent Manner. *Adv Sci (Weinh)* 10: e2300708

Niu Y, Heddes M, Altaha B, Birkner M, Kleigrewe K, Meng C, Haller D, Kiessling S (2024) Targeting the intestinal circadian clock by meal timing ameliorates gastrointestinal inflammation. *Cell Mol Immunol* 21: 842-855

Qi Z, Li Y, Zhao B, Xu C, Liu Y, Li H, Zhang B, Wang X, Yang X, Xie W *et al* (2017) BMP restricts stemness of intestinal Lgr5(+) stem cells by directly suppressing their signature genes. *Nat Commun* 8: 13824

Tsoucas D, Dong R, Chen H, Zhu Q, Guo G, Yuan GC (2019) Accurate estimation of cell-type composition from gene expression data. *Nat Commun* 10: 2975

Wang Y, Kuang Z, Yu X, Ruhn KA, Kubo M, Hooper LV (2017) The intestinal microbiota regulates body composition through NFIL3 and the circadian clock. *Science* 357: 912-916

Dr. Ye-Guang Chen
Tsinghua University
School of Life Sciences
Qinghua Yuan
Beijing, Beijing 100084
China

Dear Dr. Chen,

Thank you for the submission of your further revised manuscript to our editorial offices. I now went through this and your final p-b-p-response, and consider the remaining concerns of referee #1 as adequately addressed.

I am thus very pleased to accept your manuscript for publication in the next available issue of EMBO reports. Thank you for your contribution to our journal.

Your manuscript will be processed for publication by EMBO Press. It will be copy edited and you will receive page proofs prior to publication. Please note that you will be contacted by Springer Nature Author Services to complete licensing and payment information.

You may qualify for financial assistance for your publication charges - either via a Springer Nature fully open access agreement or an EMBO initiative. Check your eligibility: https://www.embopress.org/page/journal/14693178/authorguide#chargesguide

Should you be planning a Press Release on your article, please get in contact with embo_production@springernature.com as early as possible in order to coordinate publication and release dates.

If you have any questions, please do not hesitate to contact the Editorial Office. Thank you for your contribution to EMBO Reports.

Yours sincerely,

Achim Breiling
Senior Editor
EMBO Reports

>>> Please note that it is EMBO Reports policy for the transcript of the editorial process (containing referee reports and your response letter) to be published as an online supplement to each paper. If you do NOT want this, you will need to inform the Editorial Office via email immediately. More information is available here: https://www.embopress.org/transparent-process#Review_Process

EMBO Press Author Checklist

Corresponding Author Name: Ye-Guang Chen	
Journal Submitted to: EMBO Reports	
Manuscript Number: EMBOR-2024-60336V3	

USEFUL LINKS FOR COMPLETING THIS FORM
The EMBO Journal - Author Guidelines
EMBO Reports - Author Guidelines
Molecular Systems Biology - Author Guidelines
EMBO Molecular Medicine - Author Guidelines

Reporting Checklist for Life Science Articles (updated January
This checklist is adapted from Materials Design Analysis Reporting (MDAR) Checklist for Authors. MDAR establishes a minimum set of requirements in transparent reporting in the life sciences (see Statement of Task: 10.31222/osf.io/9sm4x). Please follow the journal's guidelines in preparing your
Please note that a copy of this checklist will be published alongside your article.

Abridged guidelines for figures
1. Data
The data shown in figures should satisfy the following conditions:
- the data were obtained and processed according to the field's best practice and are presented to reflect the results of the experiments in an accurate and unbiased manner.
- ideally, figure panels should include only measurements that are directly comparable to each other and obtained with the same assay.
- plots include clearly labeled error bars for independent experiments and sample sizes. Unless justified, error bars should not be shown for technical
- if n<5, the individual data points from each experiment should be plotted. Any statistical test employed should be justified.
- Source Data should be included to report the data underlying figures according to the guidelines set out in the authorship guidelines on Data

2. Captions
Each figure caption should contain the following information, for each panel where they are relevant:
- a specification of the experimental system investigated (eg cell line, species name).
- the assay(s) and method(s) used to carry out the reported observations and measurements.
- an explicit mention of the biological and chemical entity(ies) that are being measured.
- an explicit mention of the biological and chemical entity(ies) that are altered/varied/perturbed in a controlled manner.
- the exact sample size (n) for each experimental group/condition, given as a number, not a range;
- a description of the sample collection allowing the reader to understand whether the samples represent technical or biological replicates (including how many animals, litters, cultures, etc.).
- a statement of how many times the experiment shown was independently replicated in the laboratory.
- definitions of statistical methods and measures:
  - common tests, such as t-test (please specify whether paired vs. unpaired), simple χ2 tests, Wilcoxon and Mann-Whitney tests, can be unambiguously identified by name only, but more complex techniques should be described in the methods section;
  - are tests one-sided or two-sided?
  - are there adjustments for multiple comparisons?
  - exact statistical test results, e.g., P values = x but not P values < x;
  - definition of 'center values' as median or average;
  - definition of error bars as s.d. or s.e.m.

Please complete ALL of the questions below.
Select "Not Applicable" only when the requested information is not relevant for your study.

Materials

Newly Created Materials	Information included in the manuscript?	In which section is the information available? (Reagents and Tools Table, Materials and Methods, Figures, Data Availability Section)
New materials and reagents need to be available; do any restrictions apply?	Not Applicable	

Antibodies	Information included in the manuscript?	In which section is the information available? (Reagents and Tools Table, Materials and Methods, Figures, Data Availability Section)
For **antibodies** provide the following information:<br>- Commercial antibodies: RRID (if possible) or supplier name, catalogue number and or/clone number<br>- Non-commercial: RRID or citation	Yes	Methods and Reagents and Tools Table

DNA and RNA sequences	Information included in the manuscript?	In which section is the information available? (Reagents and Tools Table, Materials and Methods, Figures, Data Availability Section)
**Short novel DNA or RNA including primers, probes**: provide the sequences.	Yes	Table EV1

Cell materials	Information included in the manuscript?	In which section is the information available? (Reagents and Tools Table, Materials and Methods, Figures, Data Availability Section)
**Cell lines:** Provide species information, strain. Provide accession number in repository **OR** supplier name, catalog number, clone number, and/**OR** RRID.	Not Applicable	
**Primary cultures:** Provide species, strain, sex of origin, genetic modification status.	Not Applicable	
Report if the cell lines were recently **authenticated** (e.g., by STR profiling) and tested for mycoplasma contamination.	Not Applicable	

Experimental animals	Information included in the manuscript?	In which section is the information available? (Reagents and Tools Table, Materials and Methods, Figures, Data Availability Section)
**Laboratory animals or Model organisms:** Provide species, strain, sex, age, genetic modification status. Provide accession number in repository **OR** supplier name, catalog number, clone number, **OR** RRID.	Yes	Methods
**Animal observed in or captured from the field:** Provide species, sex, and age where possible.	Yes	Methods
Please detail **housing and husbandry conditions**.	Yes	Methods

Plants and microbes	Information included in the manuscript?	In which section is the information available? (Reagents and Tools Table, Materials and Methods, Figures, Data Availability Section)
**Plants:** provide species and strain, ecotype and cultivar where relevant, unique accession number if available, and source (including location for collected wild specimens).	Not Applicable	
**Microbes:** provide species and strain, unique accession number if available, and source.	Not Applicable	

Human research participants	Information included in the manuscript?	In which section is the information available? (Reagents and Tools Table, Materials and Methods, Figures, Data Availability Section)
If collected and within the bounds of privacy constraints report on age, sex and gender or ethnicity for all study participants.	Yes	Table EV

Core facilities	Information included in the manuscript?	In which section is the information available? (Reagents and Tools Table, Materials and Methods, Figures, Data Availability Section)
If your work benefited from core facilities, was their service mentioned in the acknowledgments section?	Yes	Acknowledgments

Design

Study protocol	Information included in the manuscript?	In which section is the information available? (Reagents and Tools Table, Materials and Methods, Figures, Data Availability Section)
If study protocol has been **pre-registered, provide DOI in the manuscript**. For clinical trials, provide the trial registration number **OR** cite DOI.	Not Applicable	
Report the **clinical trial registration number** (at ClinicalTrials.gov or equivalent), where applicable.	Yes	Methods

Laboratory protocol	Information included in the manuscript?	In which section is the information available? (Reagents and Tools Table, Materials and Methods, Figures, Data Availability Section)
Provide DOI OR other citation details if **external detailed step-by-step protocols** are available.	Yes	Methods

Experimental study design and statistics	Information included in the manuscript?	In which section is the information available? (Reagents and Tools Table, Materials and Methods, Figures, Data Availability Section)
Include a statement about **sample size** estimate even if no statistical methods were used.	Yes	Methods and Figure legends
Were any steps taken to minimize the effects of subjective bias when allocating animals/samples to treatment (e.g. **randomization procedure**)? If yes, have they been described?	Yes	Methods
Include a statement about **blinding** even if no blinding was done.	Yes	Methods
Describe **inclusion/exclusion criteria** if samples or animals were excluded from the analysis. Were the criteria pre-established?<br><br>If sample or data points were omitted from analysis, report if this was due to attrition or intentional exclusion and provide justification.	Not Applicable	
For every figure, are **statistical tests** justified as appropriate? Do the data meet the assumptions of the tests (e.g., normal distribution)? Describe any methods used to assess it. Is there an estimate of variation within each group of data? Is the variance similar between the groups that are being statistically compared?	Yes	Methods and Figure legends

Sample definition and in-laboratory replication	Information included in the manuscript?	In which section is the information available? (Reagents and Tools Table, Materials and Methods, Figures, Data Availability Section)
In the figure legends: state number of times the experiment was **replicated** in laboratory.	Yes	Figure legends
In the figure legends: define whether data describe **technical or biological replicates**.	Yes	Figure legends

Ethics

Ethics	Information included in the manuscript?	In which section is the information available? (Reagents and Tools Table, Materials and Methods, Figures, Data Availability Section)
Studies involving **human participants**: State details of **authority granting ethics approval** (IRB or equivalent committee(s), provide reference number for approval.	Yes	Methods
Studies involving **human participants**: Include a statement confirming that **informed consent** was obtained from all subjects and that the experiments conformed to the principles set out in the WMA Declaration of Helsinki and the Department of Health and Human Services Belmont Report.	Yes	Methods
Studies involving **human participants:** For publication of **patient photos**, include a statement confirming that consent to publish was obtained.	Not Applicable	
Studies involving experimental **animals**: State details of **authority granting ethics approval** (IRB or equivalent committee(s), provide reference number for approval. Include a statement of compliance with ethical regulations.	Yes	Methods
Studies involving **specimen and field samples:** State if relevant **permits** obtained, provide details of authority approving study; if none were required, explain why.	Not Applicable	

Dual Use Research of Concern (DURC)	Information included in the manuscript?	In which section is the information available? (Reagents and Tools Table, Materials and Methods, Figures, Data Availability Section)
Could your study fall under dual use research restrictions? Please check biosecurity documents and list of **select agents and toxins** (CDC): https://www.selectagents.gov/sat/list.htm	Not Applicable	
If you used a select agent, is the security level of the lab appropriate and reported in the manuscript?	Not Applicable	
If a study is subject to dual use research of concern regulations, is the name of the **authority granting approval and reference number** for the regulatory approval provided in the manuscript?	Not Applicable	

Reporting

The MDAR framework recommends adoption of discipline-specific guidelines, established and endorsed through community initiatives. Journals have their own policy about requiring specific guidelines and recommendations to complement MDAR.

Adherence to community standards	Information included in the manuscript?	In which section is the information available? (Reagents and Tools Table, Materials and Methods, Figures, Data Availability Section)
State if relevant guidelines or checklists (e.g., **ICMJE, MIBBI, ARRIVE, PRISMA**) have been followed or provided.	Not Applicable	
For **tumor marker prognostic studies,** we recommend that you follow the **REMARK** reporting guidelines (see link list at top right). See author guidelines, under 'Reporting Guidelines'. Please confirm you have followed these guidelines.	Not Applicable	
For **phase II and III randomized controlled trials**, please refer to the **CONSORT** flow diagram (see link list at top right) and submit the CONSORT checklist (see link list at top right) with your submission. See author guidelines, under 'Reporting Guidelines'. Please confirm you have submitted this list.	Not Applicable	

Data Availability

Data availability	Information included in the manuscript?	In which section is the information available? (Reagents and Tools Table, Materials and Methods, Figures, Data Availability Section)
Have **primary datasets** been deposited according to the journal's guidelines (see 'Data Deposition' section) and the respective accession numbers provided in the Data Availability Section?	Yes	Methods
Were **human clinical and genomic datasets** deposited in a public access-controlled repository in accordance to ethical obligations to the patients and to the applicable consent agreement?	Not Applicable	
Are **computational models** that are central and integral to a study available without restrictions in a machine-readable form? Were the relevant accession numbers or links provided?	Not Applicable	
If publicly available data were reused, provide the respective **data citations in the reference list**.	Yes	MEthods
